# Supplementary material for: Symptom appraisal, help-seeking and perceived barriers to healthcare seeking in Uganda: an exploratory study among women with potential symptoms of breast and cervical cancer
Source: BMJ Open. 2021 Feb 5;11(2):e041365. doi: 10.1136/bmjopen-2020-041365 (PMC7925866; doi:10.1136/bmjopen-2020-041365)
Supplement: Supplementary data [file bmjopen-2020-041365supp001.pdf]

In-depth Interview Guide

Understanding symptoms appraisal, help seeking and perceived barriers to healthcare seeking among women with potential symptoms of breast and cervical cancer in Uganda

Introduction

Thank you for accepting to participate in this study. The study is about understanding the way women with symptoms that could be due to breast or cervical cancer explain their symptoms and seek help for the them. In particular, we would like to understand the things that you consider when you are want to explain such symptoms as breast pain, swelling in the breast and discharge from the genitals o a woman. We also need to understand from you the factors that influence the way you seek for help.

To achieve our objective, we shall conduct an interview with you based on questions in our study guide. This interview will take about 45 minutes to one and half hours. Please keep in mind that there are no right or wrong answers to these questions. If I raise an issue or ask a question you don't want to talk about, just say so and we will move on to something else. Plase be free to ask any questions you will like to ask to help you undertand our interview. If you have any questions about the study, you can ask them below starting he interview or afterwards.

Note that you have been invited to participate in this interview because you have indicated that you have had some symptoms including pain and welling in the breasts, and discharge from your genitals during our earlier study. We are very grateful to have participate in this segment of the study.

Guiding questions

A: Detection and appraisal of bodily changes

1. **Thank you for agreeing to be interviewed today. I would like to start by asking you some questions around the changes/symptoms you mention related to your … (here relate to specific symptom mentioned e.g. breast lump, vaginal discharge).**
   Could you tell me more about this change?
   Probes:
   - What was the first thing that you noticed?
   - When did you notice the symptom? Use calendar prompt to assist, based on important landmarks e.g. Christmas, Easter, public holidays etc.
   - Has the symptom changed since you first noticed it? If yes, how has this changed?

2. **Could you describe how you felt when you noticed this symptom (refer to change/symptom e.g. finding a breast lump, vaginal bleeding)?**
   Probe:
   - Emotional response to symptom? E.g. Afraid, anxious, embarrassed, not concerned, denial, concerned that it was serious
   - Why did you feel that way?

Mwaka AD, *et al. BMJ Open* 2021; 11:e041365. doi: 10.1136/bmjopen-2020-041365

3. **When you first noticed the symptom(s), what did you think was causing the symptom? What made you think that?**
   Probe:
   - Did you think it meant you had any particular illness? If so what illness and what made you think this way?
   - At any point did you think it is/was an infection? Why did you think this?
   - Did you think it was something minor/nothing to worry about? If so, what made you think this?
   - Were you unsure about what it meant? If so what made you unsure?

4. **Since then, have you had any other thoughts about what it might be? (if changed thoughts of what the symptoms could be caused by then, probe reasons for change. E.g. what made you think that?)**

5. **Have you had a symptom like this before? If yes Probe when? What did you do? What was the outcome?**

6. **Do you know of anyone who had a similar symptom? [say the symptom]**

   If Yes, what was the cause of their symptom? What did they do about it? What was the outcome?

7. **Have you ever heard about this symptom/change anywhere? If yes where?**
   Probe: at the clinic, discussed with my family/friends, pamphlets, social media/ posters taxis or trains/ radio/television? What information did each of these sources provide about such a symptom?

8. **Did you discuss this current change/symptom with anyone?**
   Probe:
   If Yes
   - Whom?
   - What did this person/s say? Was it helpful?
   - Did it influence your views on the change/symptom? If Yes how?
   - Why did you discuss with that person (name/relation)?

   If No
   - Why did you not discuss with anyone? Probes feelings of anxiety, not worried about symptom etc.

9. **How do you think people in your community view someone with this symptom (refer to symptoms participant named)**
   Probe:
   - Is there any stigma attached to having this symptom?
   - Are people supportive/empathetic with people with these kinds of symptoms in your community?

- Is this symptom generally discussed by family and friends?

10. Has this symptom affected your life in anyway?

Probe:
If yes, how has it affected your life?

11. Have you tried to manage the symptoms yourself? Did you try anything to help relieve / resolve the symptoms?

B: Help-seeking behaviour and estimated dates of symptoms

12. Have you gone to have this change/symptom (name symptom) checked by anyone?
If YES
Probes:

- Who did you visit?
- Why did you choose to go there? Could you tell me **what** made you decide to seek care? Was there anything in particular that made you decide to seek assistance?
- When did you go to have it checked? *Use calendar prompt*
- Can you tell me about your experience seeking advice or assistance from …

Have you had the symptom checked by anyone else? (Repeat prompts above)

- Have you visited a traditional healer? If visited traditional healer, was it after or before visiting the health facilities and why before or after (rationale)? What did the traditional healer say about the symptom? *Note participant might discuss this in earlier response to question 12.*

- Have you visited a health care facility or health care provider? If yes when did you visit health care provider? Probe only if person waited to see health care provider - Is there anything that made you wait before getting checked by a health care provider?

Note participant might discuss this in earlier response to question 12.

If NO (woman has not gone to seek care)

- Have you thought about getting checked?
- Do you intend to seek care? If so, from whom? When?
- Are there any reasons for not seeking care from a healthcare provider?
- Is there anything that has stopped you from seeking care?
  e.g. lack of pain, past history of this symptom, belief that it might go away, think/thought it is/was minor, partner, family or friends
- Access - Are there any problems getting to the clinic?
  - Is the cost of getting to a clinic or health care provider a problem?

- If person working is it a problem to take time off work?
- If person looking after children is it a problem to get to the clinic because you are looking after children

Is there anything that would make you get checked by a healthcare provider?

We are now coming to the end of our discussion. Can I take a minute to pause to see if there are any questions I might have missed?

13. Do you have any other comments, or questions that you would like to ask?

Thank you for participating in this study. Your views are very important to us.

Mwaka AD, *et al. BMJ Open* 2021; 11:e041365. doi: 10.1136/bmjopen-2020-041365
