# Supplementary material for: Symptom appraisal, help-seeking and perceived barriers to healthcare seeking in Uganda: an exploratory study among women with potential symptoms of breast and cervical cancer
Source: BMJ Open. 2021 Feb 5;11(2):e041365. doi: 10.1136/bmjopen-2020-041365 (PMC7925866; doi:10.1136/bmjopen-2020-041365)
Supplement: Author's manuscript [file bmjopen-2020-041365.draft_revisions.pdf]

BMJ Open

BMJ Open is committed to open peer review. As part of this commitment we make the peer review history of every article we publish publicly available.

When an article is published we post the peer reviewers' comments and the authors' responses online. We also post the versions of the paper that were used during peer review. These are the versions that the peer review comments apply to.

The versions of the paper that follow are the versions that were submitted during the peer review process. They are not the versions of record or the final published versions. They should not be cited or distributed as the published version of this manuscript.

BMJ Open is an open access journal and the full, final, typeset and author-corrected version of record of the manuscript is available on our site with no access controls, subscription charges or pay-per-view fees (http://bmjopen.bmj.com).

If you have any questions on BMJ Open's open peer review process please email info.bmjopen@bmj.com

BMJ Open

Symptom appraisal, help seeking and perceived barriers to healthcare seeking in Uganda: An exploratory study among women with potential symptoms of breast and cervical cancer

Journal:	*BMJ Open*
Manuscript ID	bmjopen-2020-041365
Article Type:	Original research
Date Submitted by the Author:	09-Jun-2020

Complete List of Authors:	Mwaka, Amos; Makerere University, Medicine<br>Walter, Fiona; University of Cambridge, Dept of Public Health and Primary Care<br>Scott, Suzanne; Kings College London Dental Institute, Unit of Social & Behavioural Sciences<br>Harries, Jane; University of Cape Town Faculty of Health Sciences, Women's Health Research Unit<br>Wabinga, Henry; Makerere University, Medicine<br>Moodley, Jennifer; University of Cape TownUniversity of Cape Town, Faculty of Health Sciences;

Keywords:	Breast tumours < ONCOLOGY, Gynaecological oncology < ONCOLOGY, PRIMARY CARE

SCHOLARONE™ Manuscripts

I, the Submitting Author has the right to grant and does grant on behalf of all authors of the Work (as defined in the below author licence), an exclusive licence and/or a non-exclusive licence for contributions from authors who are: i) UK Crown employees; ii) where BMJ has agreed a CC-BY licence shall apply, and/or iii) in accordance with the terms applicable for US Federal Government officers or employees acting as part of their official duties; on a worldwide, perpetual, irrevocable, royalty-free basis to BMJ Publishing Group Ltd ("BMJ") its licensees and where the relevant Journal is co-owned by BMJ to the co-owners of the Journal, to publish the Work in this journal and any other BMJ products and to exploit all rights, as set out in our licence.

The Submitting Author accepts and understands that any supply made under these terms is made by BMJ to the Submitting Author unless you are acting as an employee on behalf of your employer or a postgraduate student of an affiliated institution which is paying any applicable article publishing charge ("APC") for Open Access articles. Where the Submitting Author wishes to make the Work available on an Open Access basis (and intends to pay the relevant APC), the terms of reuse of such Open Access shall be governed by a Creative Commons licence – details of these licences and which Creative Commons licence will apply to this Work are set out in our licence referred to above.

Other than as permitted in any relevant BMJ Author's Self Archiving Policies, I confirm this Work has not been accepted for publication elsewhere, is not being considered for publication elsewhere and does not duplicate material already published. I confirm all authors consent to publication of this Work and authorise the granting of this licence.

For peer review only

Symptom appraisal, help seeking and perceived barriers to healthcare seeking in Uganda: An exploratory study among women with potential symptoms of breast and cervical cancer

Amos Deogratius Mwaka[1], Fiona M. Walter[2]*, Suzanne E. Scott[3], Jane Harries[4], Henry Wabinga[5], Jennifer Moodley[4, 6, 7]

Authors' affiliations

1. Department of Medicine, School of Medicine, College of Health Sciences, Makerere University. P.O Box 7072, Kampala, Uganda.
2. The Primary Care Unit, Department of Public Health & Primary Care, University of Cambridge, UK.
3. Faculty of Dentistry, Oral & Craniofacial Sciences, King's College London, UK.
4. Women's Health Research Unit, School of Public Health and Family Medicine, Faculty of Health Sciences, University of Cape Town; Anzio Road. Observatory, 7925, Cape, Town, South Africa.
5. Department of Pathology, School of Biomedical Sciences, College of Health Sciences, Makerere University. P.O Box 7072, Kampala, Uganda.
6. Cancer Research Initiative, Faculty of Health Sciences, University of Cape Town; Anzio Road, Observatory, 7925, Cape, Town, South Africa.
7. SAMRC Gynaecology Cancer Research Centre, Faculty of Health Sciences, University of Cape Town, Anzio Road. Observatory, 7925, Cape Town, South Africa. ORCID 0000-0002-9398-5202

Emails:

Amos Deogratius Mwaka: mgratius@gmail.com

Fiona M. Walter: fmw22@medschl.cam.ac.uk

Suzanne Scott: suzanne.scott@kcl.ac.uk

Jane Harries: jane.harries@uct.ac.za

Henry Wabinga: hwabinga@gmail.com

Jennifer Moodley: jennifer.moodley@uct.ac.za

*Corresponding author: Fiona M. Walter. The Primary Care Unit, Department of Public Health & Primary Care, University of Cambridge, UK. Email: fmw22@medschl.cam.ac.uk

Word count: 7, 598.

For peer review only

Abstract

Objective: We assessed the process of recognising abnormal bodily changes, interpretations and attributions, and help-seeking behaviour among community-based Ugandan women with possible symptoms of breast and cervical cancer, in order to inform health interventions aiming to promote timely detection and diagnosis of cancer.

Design: Qualitative in-depth interviews.

Setting: Rural and urban communities in Uganda

Participants: Women who participated in the African Women Awareness of CANcer (AWACAN) cross-sectional survey who disclosed potential breast and cervical cancer symptoms were eligible; recruitment was purposive. Interviews were conducted in women's homes, lasted between 40 and 90 minutes, were audio-recorded, transcribed verbatim, and translated to English. Thematic analysis was used to identify themes and subthemes, underpinned by the conceptual framework of the Model of Pathways to Treatment.

Results: 23 women were interviewed: 10 had potential symptoms of breast cancer and 13 of cervical cancer. Themes regarding symptom appraisal and help-seeking included the; (i) detection and interpretation of abnormal bodily sensations; (ii) lay consultations regarding bodily changes; (iii) iterative process of inferring and attributing illnesses to the bodily changes; (iv) restricted disclosure of symptoms to lay people due to concerns about privacy and fear of stigmatization; (v) help-seeking from multiple sources including both traditional and biomedical health practitioners, and (vi) multiple perceived barriers to help-seeking including long waiting times, lack of medicines, absenteeism of healthcare professionals, and lack of money for transport and medical bills.

Conclusion: Women with potential symptoms of breast and cervical cancer undergo complex processes of symptom interpretation, attributing symptoms or inferring illness, and lay consultations before undertaking help-seeking and management. Increasing community

For peer review only

understanding of breast and cervical cancer symptoms, and tackling perceived barriers to health-seeking, could lead to prompt and appropriate symptom appraisal and help-seeking, and contribute to improving cancer outcomes.

Key words: Breast cancer, cervical cancer, Symptoms appraisal and attributions, help-seeking

Strengths and limitations of this study

- To our knowledge, this is the first study set in Uganda to explore the process of recognition of abnormal bodily changes, interpretations and attributions, and help-seeking behaviour among community-based women with possible symptoms of breast or cervical cancer.
- The qualitative nature of the inquiry and strong theoretical underpinning with the Model of Pathways to Treatment enabled in-depth exploration of the research question.
- Conducting interviews by female research assistants in the home of the participants allowed for freedom of expression and in-depth responses by the majority of participants.
- Self-report of symptoms and help-seeking is subject to social desirability bias especially when participants consider that their actual perceptions, beliefs and conducts could attract unfavourable comments from the research team or other people.

For peer review only - http://bmjopen.bmj.com/site/about/guidelines.xhtml

Background

In low- and middle-income countries (LMICs), including most sub Saharan African (SSA) countries, breast and cervical cancers are the most common cancers among women. The incidence of cervical cancer in Eastern and Southern Africa are 40.1 and 43.1 per 100, 000 women respectively, while that of breast cancer are 29.9 and 46.2 per 100, 000 women respectively (Bray et al., 2018). In these regions, patients with breast and cervical cancer are often diagnosed in advanced stages (Mabula et al., 2012, Pace et al., 2015, Mwaka et al., 2016). In Uganda and Ghana, 66% of cervical cancer patients were diagnosed in advanced stages (Mwaka et al., 2016, Dunyo et al., 2018). In Rwanda, 76% of breast cancer patients were diagnosed in advanced stages (Pace et al., 2015). A recent registry-based study in SSA showed that more than half (64.9%) of patients with breast cancer are diagnosed in advanced stages and experience low survival (Joko-Fru et al., 2019). Advanced stage cancer at diagnosis is associated with poorer survival (Wabinga et al., 2003, Gondos et al., 2004, Gondos et al., 2005, Sankaranarayanan et al., 2011). In SSA, breast cancer overall relative survival at one year and five years were 86.1% (84.4–87.6), and 59.0% (56.3–61.6) respectively. Relative survival varied between registries but ranged from 21.6% (8.2 – 39.8) at three years in Malawi to 84.5% (70.6 – 93.5) in Namibia (Joko-Fru et al., 2019).

Poor recognition of cancer symptoms by both patients and primary healthcare professionals may contribute to the advanced stage at diagnosis and poor survival of breast and cervical cancer in SSA (van Schalkwyk et al., 2008, Mwaka et al., 2015b, Kohler et al., 2017, Mwaka et al., 2013). Presentation at the healthcare facilities and eventual diagnosis of cancer is often long. In Ethiopia and Nigeria, 36% and 81.6% of patients with breast cancer spent more than 90 days with symptoms before seeking care with a healthcare professionals (Gebremariam et al., 2019, Ibrahim and Oludara, 2012). In Ghana, use of traditional and complementary

For peer review only - http://bmjopen.bmj.com/site/about/guidelines.xhtml

medicines were associated with prolonged time to help seeking (Brinton et al., 2017, Asoogo and Duma, 2015b). Several patient factors including individual, socio-cultural and health system factors interact to influence the patients' pathway to treatment for their cancer symptoms (Walter et al., 2012, Scott et al., 2013). In general, patients' awareness of cancer symptoms has been shown to be associated with cancer survival (Niksic et al., 2016). Similarly, in South Africa, breast cancer patients with higher education attainment and with greater awareness and knowledge of breast cancer symptoms were statistically significantly more likely to be diagnosed with early stage disease (Joffe et al., 2018). In addition, self-management, use of traditional medicines and perceived multiple barriers to medical care have been associated with longer time to healthcare seeking for breast and cervical cancer symptoms in SSA (Were and Buziba, 2001, Kazaura et al., 2007, Birhanu et al., 2012, Pace et al., 2015). In the USA, cancer fatalism among African Americans and Hispanics was associated with advanced stage at diagnosis, inadequate uptake of treatment, and less prompt healthcare seeking for cancer symptoms (Powe, 1995, Beeken et al., 2011). However, most of these studies have involved hypothetical help-seeking or retrospective accounts of those already diagnosed with cancer (Mwaka et al., 2015b, Asoogo and Duma, 2015a, Kohler et al., 2017). Hypothetical help-seeking reported by people without real symptoms may not be similar to real help-seeking decisions because of somatic and emotional experiences. On the other hand retrospective recall of symptoms and decision making processes could be enhanced or minimized because of the cancer diagnosis (Drews and Greeland, 1990, Kopec and Esdaile, 1990). In addition, help-seeking accounts of patients already diagnosed with cancers are likely to be influenced by their cancer diagnoses and multiple interactions with healthcare professionals. This may not be the case in symptomatic women without a cancer diagnosis and in the community.

For peer review only - http://bmjopen.bmj.com/site/about/guidelines.xhtml

Understanding factors influencing appraisal and help-seeking decisions for women with possible symptoms of breast and cervical cancer is a critical step to underpin the development of interventions to promote prompt recognition of symptoms and healthcare help-seeking. In this study set in Uganda, we sought to understand the processes of symptom recognition, appraisal and attribution, and perceived barriers to healthcare seeking among community women with recent experiences of possible symptoms of breast and cervical cancer, regardless of whether they had sought help or been diagnosed with cancer, in order to inform the design of interventions to promote prompt symptom appraisal and healthcare help-seeking for women in SSA.

Methods

Design

A qualitative in-depth interview study.

Study setting

The study was conducted across two sites in northern Uganda: an urban site in Gulu district and a rural site in Nwoya district. Most people in northern Uganda earn their livelihood from subsistence agricultural practices; there are low levels of education and chronic intergenerational poverty in the region (Kate Bird, 2010). Although the level of poverty has decreased since the return of peace in 2006, the region still remains the poorest in Uganda. Greater proportions of households in the region are larger in size and less likely to have two meals a day than in the rest of Uganda (MoFPED, 2014). HIV infection has consistently remained higher in this region compared to other parts of Uganda (MOH, 2006, Patel et al., 2014).

For peer review only - http://bmjopen.bmj.com/site/about/guidelines.xhtml

Study population and recruitment

This interview study was part of a larger study involving a cross-sectional survey using the AWACAN tool (Moodley et al., 2019). Eligibility criteria for this study included: women aged 18 years and over, self-reported experience of symptoms suggestive of possible breast or cervical cancer within the past three months, who had participated in the survey, spoke English or the Luo/Acholi language, and were willing to provide written consent to participate in an interview. Participants also needed to report two or more possible symptoms of breast or cervical cancer, but not have had a breast or cervical cancer diagnoses.

In the urban site, 41 potentially eligible women were identified via the survey, but 15 did not meet inclusion criteria. 14 women were purposively selected to include a range of symptoms and age. In the rural site, 25 AWACAN survey participants reported symptoms suggestive of breast or cervical cancer, but 11 did not meet inclusion criteria. All the remaining 14 were considered for interviews. Interviews were conducted during October 2018 in the urban site and January - February 2019 in the rural site; they continued until data saturation was reached in both localities (Francis et al., 2010, Guest et al., 2006).

Data collection

Two female research assistants conducted in-depth interviews in both sites. One research assistant led the interviewing process while the other operated the digital audio-recorder and took field notes, especially of non-verbal communication and events during interviews. It was necessary for the research assistants to work in pairs to ensure their personal security as they travelled in the villages. Data collection was mainly conducted in the women's homes, usually in quiet rooms without others present, and occasionally under trees in the family compounds. The research assistants used piloted interview guides, underpinned by the concepts of the Model of Pathways to Treatment (Walter et al., 2012, Scott et al., 2013). The interview guide

For peer review only - http://bmjopen.bmj.com/site/about/guidelines.xhtml

included how bodily changes were first recognised, what happened immediately after detection of a symptom, who were consulted about the symptoms, and the steps undertaken to seek help (see Supplementary File 1). Socio demographic data including age, marital status, number of biological children, highest educational attainment, and HIV status were also collected. Each interview lasted between 45 and 90 minutes, and all interviews were audio-recorded.

Data analysis

Audio-recordings were transcribed verbatim by the research assistants. ADM read transcripts and conducted preliminary analyses during data collection in order to determine data saturation. Before full analyses, ADM reviewed ten transcripts (five from each site) against the recordings to ensure appropriate details were captured and transcripts labelled appropriately. ADM read three transcripts in detail, developed codes, and shared the codebook with the wider study team. Codes were redefined and refined as necessary. Atlas.ti version 6.1 was used to support data analysis. Coded data segments from all transcripts were aggregated and retrieved for detailed analyses. Themes and subthemes were inductively developed from the data while focussing on the research objective and the concepts in the Model of Pathways to Treatment.

Patient and Public Involvement

Patients were not involved in the planning and implementation of this study. However, the public was involved through the district and community leaders. The district health officers and district women representatives on the district councils of the two study districts were involved in the study. These local leaders were part of our Project Advisory Committee (PAC) which we formed to provide advice and guidance to the study team to ensure that the study is conducted in a culturally acceptable manner, and the findings are relevant to the local context in order to inform local and national health policies on targeted interventions to promote cancer

For peer review only

For peer review only - http://bmjopen.bmj.com/site/about/guidelines.xhtml

awareness and early detection. The study objectives and tools were discussed with these stakeholders. They provided guidance on how our study could refined to best fit the local context. We therefore refined the study tools to capture aspects of women's lives that relate to information seeking and access, healthcare seeking, and decision making in the families. In addition, the local leaders will be involved in disseminations of study findings. Their presence during dissemination is expected to make the population more confident in the results. The involvement of the local decision makers potentially increases the chance that this study findings could be acceptable and put into use.

Results

Characteristics of the participants

23 women were interviewed: 10 had possible symptoms of breast cancer and 13 had possible symptoms of cervical cancer. Two self-reported being HIV positive and on antiretroviral therapies. Overall, the median age was 31 years (range: 18 – 66 years; urban site 27 years (range: 22 – 66 years), rural site 41 years (range: 18 – 64 years)), (see **Table 1).**

For peer review only

Table 1: Characteristics of respondents and reported symptoms

	**Study site**		Age bracket (Years)	**Marital status**	**Education attainment**	**Occupation**	**Symptoms**	**HIV status**
**Participants**	Urban	Rural						
P1	X		31 - 50	Widow	Primary 5	Unemployed	Pain and lump in breast	Negative
P2	X		31 - 50	Married	Primary 4	Business/trader	Pain and lumps in both breasts	Positive
P3	X		51 - 70	Widow	Primary 6	Business/trader	Pain and lump in breast	Negative
P4	X		18 - 30	Separated	Primary 3	Waitress in restaurant	SVD*, Vaginal bleeding between periods, Persistent LAP*	Negative
P5	X		18 - 30	Married	Senior 4	Business/trader	Pain and lump in breast	Negative
P6	X		18 - 30	Married	Senior 4	Business/trader	Vaginal bleeding during/after sex, Persistent LAP	Negative
P7	X		18 - 30	Married	Tertiary education	Business/trader	Pain and lump in breast	Negative
P8	X		18 - 30	Married	Senior 3	Business/trader	SVD, Persistent LAP, Pain and discomfort during sex	Negative
P9	X		31 - 50	Widow	Primary 3	Business/trader	SVD, Persistent LAP	Negative
P10	X		18 - 30	Married	Primary 7	Unemployed	Lumps in armpits and both breasts	Negative
P11	X		51 - 70	Married	Primary 6	Unemployed	Pain and lumps in both breasts	Unknown
P12		X	51 - 70	Married	No formal education	Unemployed	Lumps in armpits and both breasts	Negative
P13		X	31 - 50	Married	Primary 2	Unemployed	SVD, Persistent LAP, Pain and discomfort during sex	Positive
P14		X	18 - 30	Married	Primary 5	Unemployed	Pain in nipple, lump in breast	Negative
P15		X	31 - 50	Married	Primary 2	Unemployed	Lump and pain in breast, dimpling of breast skin like orange peel	Negative
P16	X		18 - 30	Separated	Primary 6	Unemployed	SVD, Persistent LAP	Negative
P17		X	31 - 50	Married	Primary 6	Business/trader	Persistent LAP, discomfort during sex	Negative

For peer review only - http://bmjopen.bmj.com/site/about/guidelines.xhtml

P18		X	31 - 50	Married	No formal education	Unemployed	Persistent LAP, discomfort during sex	Negative
P19		X	51 - 70	Married	Primary 1	Unemployed	SVD, Persistent LAP, Pain and discomfort during sex	Negative
P20		X	18 - 30	Married	Primary 3	Unemployed	SVD, Vaginal bleeding between periods, Persistent LAP	Negative
P21		X	31 - 50	Married	Primary 3	Unemployed	SVD, Vaginal bleeding between periods, Persistent LAP	Negative
P22		X	18 - 30	Married	Primary 7	Unemployed	Persistent LAP, discomfort during sex	Negative
P23	X		18 - 30	Married	Tertiary education	Accountant	SVD, Persistent LAP, Itching in the vagina	Negative

*LAP = Lower abdominal pain, SVD = Smelly vaginal discharge; Pn refers to respondents number as arranged in the analysis software.

For peer review only

Main themes

Themes regarding symptom appraisal and help-seeking intervals included the; (i) detection and interpretations of abnormal bodily sensations; (ii) lay consultations regarding bodily changes; (iii) iterative process of inferring and attributing illnesses to the bodily changes; (iv) nondisclosure of symptoms to lay people due to concerns about privacy and fear of stigmatization; (v) help-seeking from multiple sources including both traditional and biomedical health practitioners, and (vi) multiple perceived barriers to biomedical care. Supporting quotations are presented followed by participant characteristics (study ID, urban/rural setting, age, and symptoms of cancer type).

(i) Detection and interpretation of abnormal bodily sensations

Detection and initial interpretation of bodily changes as normal or abnormal punctuated participants' thoughts and course of actions regarding their symptoms. The circumstances when bodily changes were first realised were critical to initial interpretations.

Expected bodily changes within a person's lifespan

The age of the woman was critical in deciding whether a certain bodily change could be due to an illness or normal changes. Young women could readily associate bodily changes with menstrual cycles and possibilities of pregnancies, while older women were more concerned with menopausal changes and age-related ailments.

> "That problem, I thought it was my state of menopause that I reached and I stopped giving birth. I thought that way because I wasn't conceiving or giving birth anymore", (**P19_Rural_xx yrs_Cervical**).

For peer review only - http://bmjopen.bmj.com/site/about/guidelines.xhtml

Participants who had ever breastfed or been pregnant initially perceived their breast symptoms to be due to usual bodily changes, and paid little attention until symptoms persisted or became painful

> "When this lump came up, I thought that probably it is as usual when it would happen when I'm breast feeding. But now I'm not even breast feeding, but the lump on my right armpit is still there and painful", (**P12_Rural_xxyrs_Breast**).

Chronology with recent events

Bodily changes were interpreted based on circumstances around their onset. For example, a participant who started to experience abnormal bodily changes following a medical procedure or surgery would infer the changes to the procedure.

> "I thought that was an effect from the contraceptive I had inserted in my arm. Before I started using that implant, those symptoms were not there; it was not happening", (**P5 _Urban_xxyrs_Breast**).

Some participants interpreted their bodily changes in respect to their medical histories including post-abortion complications and effect of birth trauma following deliveries.

> "I don't really know [. . .]. I also had a miscarriage with him but my uterus wasn't "cleaned". The doctor said after such an incident one has to undergo "cleaning" of the uterus in order to be healthy once again. So that is why I feel it was that problem causing

For peer review only - http://bmjopen.bmj.com/site/about/guidelines.xhtml

the symptoms [. . .]. I feel that probably there are some remaining dirt in my womb", (**P18_Rural_xxyrs_Cervical**).

Poor personal hygiene, crowded living environment and risk of infections

Living in crowded unhygienic dwellings especially rented units influenced the initial interpretations of bodily changes experienced. Women considered personal hygiene as critical determinants of gynaecologic symptoms. Women who thought their personal hygiene was compromised were quick to think of hygiene-related causes or infections, especially if they did not have a sexual partner who they could suspect for transmitting a sexually transmitted disease. This view was predominantly held among younger urban women with gynaecologic symptoms.

For peer review only

"I first thought it could be candida . . . because it also causes itching in the vagina. I thought of the fact that there are many tenants here, so the issue of sharing the bathroom could be the problem; you could easily get it", (**P8_Urban_xxyrs_Cervical**).

"May be poor hygiene, because from school you bathe in a crowded place. You bathe where everybody shares things; you share the buckets. So I think poor hygiene was one of the factors, yeah", (**P23_Urban_xxyrs_Cervical**).

(ii) Lay consultations regarding bodily changes

Several factors and concerns influenced women to share their symptoms with other people, including husbands, siblings, trusted friends and sometimes parents. Participants engaged in lay consultations for various reasons.

For peer review only - http://bmjopen.bmj.com/site/about/guidelines.xhtml

Seeking financial and practical support

Most participants shared their symptoms with significant others to solicit financial and practical support. These women needed to reach the healthcare facilities for care, but lacked money or means of transport.

> "I thought that being my husband, if I told him he would take me to the health facility. He told me that he would have taken me . . . but he doesn't have the money. I didn't share it with anyone else because nobody else could help me with the money", (**P17_Rural_xxyrs_Cervical**).

Learn and benefit from experiences of others

Many participants shared their symptoms with other people either because they trusted them or wanted to know more from them to help them decide the course of actions for their symptoms. Some of the confidants were healthcare professionals related to the participants or had had similar symptoms in the past and or were considered to know more about the symptoms.

> "I tried to tell my Mother in-law, and also my sister in-law who had recently returned home, then later I told my husband. I shared with her because I felt she was a mature person and could be having some ideas about it. So she just responded by saying; that definitely needed me to go to the hospital and was not to be tampered with [. . .]. I chose her because I felt she was close to me and we were relating so well", (**P14_Rural_xxyrs_Breast**).

> "They looked to be more informed that is why I also went to them. One of them told me that she also experienced those symptoms when she was pregnant but now it's not

For peer review only - http://bmjopen.bmj.com/site/about/guidelines.xhtml

> there. That she went to the health facility and was treated, so she was saying that if I also go and get treatment I will feel better", (**P8_Urban_xxyrs_Cervical**).

Social accountability and avoidance of suspicion from family

A few participants shared with family, especially when the woman found it difficult to accomplish her roles in the family because of her illness.

> "The reason as to why I decided to tell them is because when I have the pain sometimes I need to lie down for a while to rest and calm the pain. But if you don't tell them (in-laws) they may think you are just a lazy woman who doesn't want to do work, so I had to tell them so that they can be aware", (**P7_Urban_xxyrs_Breast**).

A few participants shared their gynaecologic symptoms with their husbands not just to get financial support to seek care, but rather because they wanted to avoid their husbands suspecting them of engaging in sexual intercourse with other men.

> "I decided to share with him (husband) because I didn't want him to suspect me of having cheated on him and got the infection. So I told him that I had never cheated on him or had these kind of symptoms before; so it was him who had infected me with it", (**P20_Rural_xxyrs_Cervical**).

(iii) Nondisclosure of symptoms to lay people

While most participants shared their symptoms, most were keen not to share with people they did not trust.

For peer review only - http://bmjopen.bmj.com/site/about/guidelines.xhtml

Privacy concerns

Majority of participants considered symptoms of breast and cervical symptoms as private and not appropriate to share with just anyone.

> "By the way sharing your personal issues anyhow with anyone makes you become a fool. For me, once you are mature enough, don't go out sharing your issues anyhow", (**P18_Rural_xxyrs_Cervical**).

Stigma and gossiping

Some participants who did not share their symptoms were concerned about gossiping and becoming objects of public discussions.

> "Some people like rumour mongering. For example, they can say such a person said this, they could now be having HIV/AIDS. They can talk; that you know, this person said they are in this state and that is already rumour mongering done. So they will be circulating your name. It is because I fear lies of people [. . .]. That is why it is very hard to share with people any secrets, because once you do, news go all over. This is why I no longer like sharing anyhow", (**P19_Rural_xxyrs_Cervical**).

Self-perceived stigma could be an issue among symptomatic women, especially those with cervical symptoms.

For peer review only - http://bmjopen.bmj.com/site/about/guidelines.xhtml

"As for other people, if you start to share your issues with them, they may judge you without assessing; they will end up airing your issues to people with other funny added lies", (**P13_Rural_xxyrs_Cervical**).

Low expectations from lay consultations

Some participants had low expectations from lay consultations and therefore preferred formal healthcare seeking as the only and best response to illness symptoms.

"I felt I should go straight to the hospital so that the doctor himself examines me; to me sharing with someone else here wouldn't yield much because they may mention something that will never be real compared with what the doctors will examine and find out", (**P9_Urban_xxyrs_Cervical**).

(iv) Symptoms attributions: inferring illness

Participants attributed their symptoms to various disorders including infections and cancers. Symptom attributions were guided by the nature of onset, evolution and persistence of symptoms, participants' own previous experiences, family history of illnesses, response to attempted treatments, sanctioning/disapprovals from lay consultations, and messages from the radio or healthcare professionals.

Symptoms could be due to cancer

Gynaecologic symptoms that were unfamiliar and which persisted were often attributed to cervical cancer. These women expressed a lot of worries as they were inferring illness, mostly related to death and orphanhood.

For peer review only - http://bmjopen.bmj.com/site/about/guidelines.xhtml

"I have been asking myself what this could be! It kept me worried a lot. It was worrying me; I used to say that the symptoms I'm experiencing could be cancer and if it is, then that would be too bad. That is what was moving in my mind [. . .]. I just felt that cancer disease is one that can definitely lead to things such as that. I thought so because cancer is a very bad disease, it is really a bad disease . . . it hurries you and you will be short lived compared to other diseases", (**P9_Urban_xxyrs_Cervical**).

Participants who ever screened for cervical cancer considered cancer as possible explanation for their symptoms but were unsure as they had tested negative on earlier screening tests.

"I have been worried about these symptoms and signs. I first thought it was cancer, but I went for cancer screening two years ago, so am still waiting for one more year to go and test again. I thought it could be cancer because cancer is the most dangerous infection, so people fear it most", (**P6_Urban_xxyrs_Cervical**).

"I also thought about cancer but I had already tested and it was negative so they had told me to test again after three months - oh no! They said three years. I gave birth in 2013, so I tested in 2014. They had told me to go back in the previous year, 2018, but I haven't gone back again . . . I thought that probably it is the one starting. Because I heard that when you start experiencing smelly vaginal discharges that could be a sign of cancer. All I thought about was that it could be syphilis, if not, then cancer", (**P17_Rural_xxyrs_Cervical**).

Severity and persistence of symptoms as well as messages from healthcare professionals informed and or reinforced participants' attributions of symptoms to possible cervical cancer.

For peer review only - http://bmjopen.bmj.com/site/about/guidelines.xhtml

"[My] lower back/abdominal pain made me think it was cervical cancer causing these symptoms. Because the lower abdominal pain was too much. But also I heard from the health facility. They were educating those who had gone to test for that cancer. The healthcare provider said that you can get this cancer from a man who has had sex with a woman who has the virus then he can transmit it to you through sex", (**P16_Rural_xxyrs_Cervical**).

Persistence of bodily changes beyond ordinary expectations and severity of symptoms caused participants to review their attributions, often considering more serious illnesses including cancers.

"I thought probably it could be cancer because of the much pain I was experiencing on my breast, or that it was just normal pain resulting from the back pain I sometimes experience. But then sometimes I wonder why one side of my breast has a lump and the other doesn't; so all these leave me in wonder of what could be the problem", (**P1_Urban_xxyrs_Breast**).

Participants who were aware of breast cancer symptoms immediately attributed the changes experienced in their breasts to possible breast cancer. Most reported that they commonly learn about symptoms of illness from radio programs.

"The reason as to why I thought it was breast cancer is because I always listen to the radio and these doctors always talk about cancer and how cancer can start and also the

For peer review only - http://bmjopen.bmj.com/site/about/guidelines.xhtml

signs and symptoms of cancer on people. So they tell you when you see such signs, please go to hospital", **(P2_Urban_xxyrs_Breast)**.

Persistent and relentless pain not responding to commonly used medications or home-based remedies often led participants to think of more serious illnesses including cancers.

"I thought it could be cancerous because of the pain. The pain used not to cool before I went to the hospital; so I thought it was the toughest disease, cancer [. . .]. At least HIV/AIDS, it is easy to get drugs for it, but this cancer has no drug, till you die and get buried; and that is its medicine", (**P11_Urban_xxyrs_Breast**).

Sexually transmitted infections and other gynaecologic infections

Participants who had symptoms of vaginal itching and discharge readily attributed the cause to candidiasis, believed to be transmitted both through sexual intercourse and poor personal hygiene. Some attributed their bodily changes to sexually transmitted infections, and blamed symptoms on their husbands who were often considered promiscuous.

"I thought that it could be some sexually transmitted infection. I was thinking that it could be gonorrhoea because if it were syphilis, I would have known it because it comes with a lot of pain, and I don't have the smelly vaginal discharge", (**P6_Urban_xxyrs_Cervical**).

"I thought that probably my husband cheated on me with an infected person because I had never experienced these symptoms. […] ever since he was away for four months from October to February without coming home even one single day, then when he

For peer review only - http://bmjopen.bmj.com/site/about/guidelines.xhtml

comes back after sometimes, I start experiencing these symptoms! So I had every reasons to be suspicious", (**P17_Rural_xxyrs_Cervical**).

(v) Help-seeking for symptoms

Women sought help from both biomedical and traditional health practitioners including faith-based healers. Participants chose a particular help-seeking route depending on perceived causes, knowledge of previous similar illnesses that responded to a particular treatment approach, advice from social networks, and messages from the media especially local radio health programs.

Healthcare help-seeking

Most participants sought care in healthcare facilities because they needed tests to confirm the causes of their health problems; they felt these would direct them to the appropriate remedies.

"I went to the doctor because I know he is the only person who can help with my condition; they have testing kits and they can confirm without doubts. They have machines that test and confirm the truth. Those things that are just guessed are not helpful at all", (**P9_Urban_xxyrs_Cervical**).

Most sought care in healthcare facilities whenever symptoms worsened and or caused worries, or when home-based remedies did not lead to improvement in symptoms. Persistent pain was a very common trigger for healthcare seeking. The media, especially radio talk shows, encouraged a significant proportion of participants to seek care at health facilities.

For peer review only - http://bmjopen.bmj.com/site/about/guidelines.xhtml

“For me the first signs I saw was the swelling of my breast and I warmed water and started massaging on the swollen part but it didn’t help, so I decided to go to hospital and told the doctor. Another reason as to why I visited the hospital with the symptoms of my sickness is because of the constant teaching and reminders by doctors over the radio during their radio talk shows that whenever you have any signs and symptoms which is unusual, you should go to hospital. […] I believe if you go to the hospital early enough with any sickness the doctors will find the name of the sickness and cure you if possible”, (**P2_Urban_xxyrs_Breast**).

“I went to the clinic, bought medicine and took it. But there was no improvement that’s why I decided to go to the hospital. It was in March this year (2018) that I went to the clinic; but this pain started after giving birth. What made me go to the hospital was because I was worried. I was worried that I could be having a sexually transmitted disease or cervical cancer”, (**P6_Urban_xxyrs_Cervical**).

Advice from healthcare professionals when women go for antenatal and postnatal care, and immunization for their children motivated them to seek care whenever they experienced symptoms of illnesses.

“They say it; doctors always talk when we take our children for immunization. Those responsible for the hygiene and sanitation of the hospital once you take your children for immunization or antenatal care, they often tell us that once we get to see any funny signs of any sickness, we need to rush to the nearest health facility early enough. Otherwise the symptoms will worsen, because if you feel pain in your body, you need

For peer review only - http://bmjopen.bmj.com/site/about/guidelines.xhtml

> to go to the hospital. So I took that up and rushed to the hospital because I was also feeling that in my body", (**P21_Rural_xxyrs_Cervical**).

Participants were also motivated to seek biomedical care for concerns regarding the welfare of their children. They would want to remain alive and nurture their children their own way.

> "The reason why I decided to go to the hospital that time when I was feeling the symptoms of my lower abdomen and the waist, is because I decided that I should stay healthy . . . to care for my children. I should surely remain healthy and keep them", (**P13_Rural_xxyrs_Cervical**).

Perceived inconveniences to others and personal discomfort due to symptoms was another factor that triggered healthcare seeking among participants. Symptoms of cervical cancer including smelly vaginal discharge and excessive vaginal bleeding can be very inconveniencing and restrictive to the movement of a woman.

> "I went because I was not comfortable among my fellow girls; you could see others are happy and for me I have my hidden problems, so I had to go and solve it with the doctor", (**P23_Urban_xxyrs_Cervical**).

Traditional and complementary medicines

A few participants sought care from traditional health practitioners including herbalists and faith-based healers who used prayers, sometimes combined with herbal preparations to treat different ailments. Some participants thought traditional and complementary medicines (T&CMs) could be useful while others contended that T&CMs are fake, and that the

For peer review only

For peer review only - http://bmjopen.bmj.com/site/about/guidelines.xhtml

practitioners are dishonest. Most participants thought that T&CMs do not work. They said the medicines are given based on guess work, as there are no tests done to identify cause of illness. These negative views about T&CMs were held by participants from both urban and rural sites, and with both breast and cervical symptoms.

> "I don't even think about it because some usually walk past here hawking herbal drugs claiming they have what treats ulcers, blood pressure, cancer and many more diseases, but I always tell them I don't have money to buy and that I don't use herbs. There is nothing that can attract me in buying local herbs. My opinion is that traditional herbs nowadays don't work or not effective", (**P1_Urban_xxyrs_Breast**).

> "With traditional medicines, you can be given just anything that will not even help you. So I will never make a commitment to use traditional medicine. They may even just go and pluck the leaves of black jack then they brand it that it is medicine for such and such a disease and yet in actual sense, it will not be the cure for that disease. So most often I stick to my decision of going to the hospital because their medicines will have been tested", (**P9_Urban_xxyrs_Cervical**).

> "I haven't got any medicine from any traditional healer and I personally don't want. To tell you, these traditional medicines even if you are given in regards to these symptoms in the stomach, they will not work . . . So I really wonder whether the traditional medicines really treat those diseases", (**P18_Rural_xxyrs_Cervical**).

A few of the participants who admitted to using T&CMs testified that T&CMs, the ones they used, did not work.

For peer review only - http://bmjopen.bmj.com/site/about/guidelines.xhtml

> "I don't support that, and . . . I will stop T&CM use because I personally tried and it failed to work; it didn't help me with anything. It is true . . . I was instructed to go get the traditional medicine . . . I first went to that other T&CM practitioner, then I went to the nurse", (**P10_Urban_xxyrs_Breast**).

> "I tried taking traditional medicine in vain, and when I tried to use that without success, then my husband told me that its better I try to go to the hospital instead so that it is the doctor to feed me with the right information. I used to dig out the roots then I would drink the juice. No improvement was realized", (**P19_Rural_xxyrs_Cervical**).

On the other hand, a substantial minority of participants who had ever used a T&CM reported that they are effective and can be used alongside modern medicine. They felt that when the cause of an illness is due to malevolent spirits or breaching cultural norms, then traditional health practitioners are helpful. Participants from the rural site were more likely to report use of T&CM than those from the urban site.

> "When I used to pass sticky stool and also felt pain in the lower abdomen, the thing that helped me the most was one traditional medicine . . . it was called *tusua* (not real name). That is a type of tree . . . I would dig out the roots then pound it until fine, then I boil for long, then I filter it into the cup, and afterwards when it cools I take it, then it would end up the stickiness of my stool immediately. I also used to have tiny itchy rashes in between my thighs. […] I used to break the leaves of *okala* tree (not real name). You know that tree has some whitish sap, so I would harvest that and place on the spot of

For peer review only - http://bmjopen.bmj.com/site/about/guidelines.xhtml

the blisters and the sap from *okala* ended up curing, till to date there are no rashes", (**P13_Rural_xxyrs_Cervical**).

(vi) Perceived barriers to healthcare help-seeking

Participants discussed several barriers to prompt healthcare help-seeking. These included health system challenges including lack of medicines, long waiting time to see healthcare professionals, lack of money, and competing social obligations.

Lack of medicines in the public health facilities

Most participants who contemplated healthcare help-seeking for their symptoms lamented the sorry state of the public healthcare facilities. They reported that there were often no medicines available.

"I just get discouraged with the thought of going there and being told to buy the recommended drugs from outside", (**P3_Urban_xxyrs_Breast**).

"They kept telling me that the drugs they need to inject me with is out of stock! I went back two times and each time the same story, so I never went back again", (**P4_Urban_xxyrs_Cervical**).

Long waiting times

Due to the long queues and extended waiting times to see the healthcare professionals, especially in the public health facilities, participants often said they would consider alternative

For peer review only - http://bmjopen.bmj.com/site/about/guidelines.xhtml

avenues to healthcare help-seeking. This view was more common among participants with breast symptoms especially from the urban site.

> "The main referral hospital also has its own problems; you can take like two days while still on the line to see the doctor. It is equivalent to someone coming all the way from Sudan and going back; that is the line to see the doctor. Whenever you are almost at the front of the line, the doctor leaves. So when you combine all the waiting with hunger and ulcers, you end up giving up", (**P3_Urban_xxyrs_Breast**).

> "I'm just planning to go in this coming February. [The] hospital is usually very crowded; if you go there you can spend a long time without getting treatment", (**P16_Rural_xxyrs_Cervical**).

Absence of healthcare professionals at the healthcare facilities

Participants from both urban and rural sites reported visiting healthcare facilities several times but not finding the appropriate healthcare professionals to address their concerns.

> "As you know these government hospitals, services are not like for other private hospitals. So the experience I had was - the first time I went to the hospital, they recommended that I first have a scan done, but the person who was supposed to do it wasn't around so they told me to go back the next day, which I did and they told me the person supposed to do it was still not there so I should go back on Monday, (the following week)", (**P1_Urban_xxyrs_Breast**).

For peer review only - http://bmjopen.bmj.com/site/about/guidelines.xhtml

> "I went and the healthcare provider picked the referral note and put it in the file where they had written my complaints. Then I was told that the doctor isn't there and that I should just go back on Friday, when the doctor will be around and also the ultra-scan machine will also be working, so that they can also do an ultra-scan the next Friday", (**P22_Rural_xxyrs_Cervical**).

Lack of money for healthcare help-seeking

Most participants reported financial constraints to seeking healthcare in the private facilities where there would be no crowding, and medicines available, and described unaffordable fees for consultations, tests and treatments.

> "There is really pain, but the main reason why I didn't go is the lack of finances that opens every door", (**P15_Rural_xxyrs_Breast**).

Participants reported that patients sometimes had to sell whatever they have to get some money to seek care in the private health facilities where they expect reasonable care.

> "There is nothing else, because for the three times I visited [the private] hospital, and two times in the government hospital I have been selling goats of which they are now finished. There is nothing more to sell, and yet I have not yet received any improvement", (**P15_Rural_xxyrs_Breast**).

Rural participants also mentioned competing demands for the little money they got from selling off their belongings to support their medical expenses.

For peer review only - http://bmjopen.bmj.com/site/about/guidelines.xhtml

"So when I get money I will seek for help in the health facility but for now it's tricky because I have grown up children and grandchildren who are under my care. So getting the money to go to the health facility is a problem. And as you also know *Lexam* (not real name) hospital, all the services there require money. […] I plan to go to *Lexam* hospital because *Takila* regional referral hospital (not real name) is quite far away, *Lexam* is nearer to our home here", (**P12_Rural_xxyrs_Breast**).

"It is the issue of lack of money that prevented me from going. […] I am struggling so that I raise some money to buy books for my children and enrol them in school then I will think of going to the hospital. I do want to seek help but the means to access that is what is still defeating me. I need, because I also love my body. It is only the issue of money and if I had it today, I would go straight to the hospital", (**P21_Rural_xxyrs_Cervical**).

Transport money to the public health facilities was another challenge for women, especially those from the rural areas.

"I'm still looking for money to go with to the hospital. I will go when I get the money. The money is for transport. It is 12,000= (USD 3.3) one way, so I need 24,000= (USD 6.6) for transport on a boda boda (*hired motor cycle*)", (**P20_Rural_xxyrs_Cervical**).

For peer review only - http://bmjopen.bmj.com/site/about/guidelines.xhtml

Discussions

Main findings

This study is one of the first community-based inquiries into symptom recognition, interpretation, appraisal, illness attribution, and consequent help-seeking among women with possible breast and cervical cancer symptoms in urban and rural Uganda. We found that women undergo a complex process of sense-making and help-seeking for their symptoms. The detection and interpretation of abnormal bodily sensations were interpreted based on expected bodily changes within a person's lifespan, perceived poor personal hygiene, and crowded living environments with risk of infections. Upon realising abnormal bodily changes, most participants engaged in extensive lay consultations with relatives, friends and acquaintances mainly because they wanted to solicit financial and practical support, learn and benefit from experiences of others regarding the symptoms, and for social accountability and avoidance of suspicion from husbands/families especially when symptoms were disabling and restricting participations in their daily chores. On the other hand, a substantial minority of participants did not disclose their symptoms to lay colleagues mainly because of low expectations of support, concerns with privacy, avoidance of possibility of gossiping, and concern with stigmatization from the community. Medical pluralism was common, with participants often seeking help for symptoms from both biomedical healthcare facilities and traditional and complementary medicine providers including faith-based healers, despite many being sceptical about the effectiveness of traditional and complementary medicines.

Our findings regarding recognition of abnormal bodily changes, interpretation and symptom attributions, as well as disclosure/nondisclosure of symptoms to lay people, are similar to findings from studies in other SSA countries such as Malawi and Ghana among breast cancer patients (Kohler et al., 2017, Bonsu and Ncama, 2019). The patients appraised their symptoms

For peer review only - http://bmjopen.bmj.com/site/about/guidelines.xhtml

within the context of their life events and situations, and were guided by expected bodily changes, family history of cancer and suggestions from lay consultations. The attribution of symptoms changed over time depending on characteristics of the symptoms, including their nature, evolution, response/nonresponse to common medications, and advice from lay people. Understanding the life context of people could inform the content, delivery and timing of health interventions to promote prompt and appropriate symptom recognition and help-seeking.

Our findings suggest that Ugandan women with vaginal symptoms often first attribute their symptoms to gynaecologic infections such as candidiasis and sexually transmitted infections. Only if symptoms persisted or did not respond to medications did women sometime attribute their symptoms to cancer. Similar symptoms for cancers and infectious diseases can offer great challenges to women especially if the illnesses have different pathways to care and treatment. In our earlier studies among women with an established diagnosis of cervical cancer in a hospital in the same region, most women attributed their vaginal symptoms of bleeding and discharge to cervical cancer and sexually transmitted infections. Most of these women sought care after long time periods and presented with advanced stage cancer (Mwaka et al., 2016). In contrast, we found that women with breast symptoms who had heard of breast cancer mainly from radios and healthcare professionals during earlier visits to health facilities quickly attributed their symptoms to breast cancer and sought care accordingly. Similar findings were reported in an earlier Ugandan study about symptoms of cervical cancer (Mwaka et al., 2015b). This concurs with evidence from the UK, showing that awareness and knowledge of cancer symptoms are key in shaping people's attributions of symptoms and often prompt healthcare help-seeking (Simon et al., 2010). We also found that pain, persistence of symptoms, or symptoms interfering with daily routines could trigger decisions to seek help. Mild and intermittent symptoms were either ignored or interpreted as normal bodily changes, for

For peer review only - http://bmjopen.bmj.com/site/about/guidelines.xhtml

example, due to the menopause or pregnancy, concurring with findings from a Malawi study of women with breast cancer (Kohler et al., 2017).

Women with potential breast and cervical cancer symptoms often engaged in lay consultations with their husbands, relatives and friends regarding their symptoms. These lay consultations were found to not only influence symptom attributions and illness inference, but also women's' help-seeking intentions and behaviour. Participants consulted with other people to obtain financial and practical support to seek help, as well as to gain insights into the causes and best effective remedies for their illnesses. Sanctioning of initial symptom interpretations by significant others has been shown in other studies set in the UK and Australia to influence help-seeking decisions among people with possible cancer symptoms or recently diagnosed with cancer (Macleod et al., 2009, Emery et al., 2013). To avoid misleading advice when symptomatic women seek help from peers and significant others, the community as a whole need to be aware of cancer risk factors, symptoms and treatment. Therefore, public awareness campaigns to improve general awareness is important so that when women share their symptoms with other lay people, they may get appropriate help.

We found surprising negativity about traditional and complementary medicines, although some continued to consult traditional healers. This contrasts with earlier Ugandan qualitative findings from asymptomatic men and women, revealing that traditional and complementary medicines occupied a reasonable place in their lives mainly because the medicines were believed to work as they worked for their grandparents and ancestors, and could be useful to fill the void created by multiple barriers to biomedical care (Mwaka et al., 2015a). The participants in this study perceived the research assistants as healthcare providers; indeed, one

For peer review only - http://bmjopen.bmj.com/site/about/guidelines.xhtml

was a nurse. Therefore, it is possible that the reported low opinion towards use of T&CM could be due to social desirability bias.

Participants in this study reported several individual, interpersonal, sociocultural and health system structural barriers that delayed or prevented them from seeking healthcare. In public health facilities these barriers included lack of medicines, long waiting times and queues, and absenteeism among healthcare professionals. At a personal level, women commonly reported lack of money for transport and medical bills as their main barrier, especially in private healthcare facilities where patients pay directly for care. Most women also reported competing priorities and obligations that would hinder them from seeking healthcare. These obligations not only competed for their time but also for financial resources which were often diverted to school fees and other priorities instead of transport to health facilities and medical bills. Similar challenges to breast and cervical cancer healthcare help-seeking have been reported in other studies in SSA (Kohler et al., 2017, Mwaka et al., 2015a, Mwaka et al., 2013). Insights into the perceived barriers to, and contextual factors that influence healthcare seeking from perspectives of the women can potentially inform targeted interventions to promote prompt healthcare seeking. Imposing health programs on women without clearly understanding their perspectives and contextual factors may not have the desired impact and would be an inefficient use of resources. Health planners and policy makers therefore need to make research findings to inform their decisions.

Limitations

Our study has some limitations. The study was preceded by a cross-sectional survey in which women were asked about potential breast and cervical cancer symptoms. It is possible that this could have influenced how women perceived and interpreted their symptoms.

For peer review only - http://bmjopen.bmj.com/site/about/guidelines.xhtml

Second, conducting interviews in women's homes may have influenced how some participants responded particularly in terms of the influence of family and social networks. However, we ensured that there were no non-participants at the interview venues.

Third, self-report of symptoms and help-seeking is subject to social desirability bias especially when participants may consider that their perceptions, beliefs and conduct could attract unfavourable comments from the research team or other people. However, as the research participants were assured of confidentiality, we contend that our findings accurately represent the perceptions and help-seeking behaviour of the symptomatic women included in this study.

Conclusion

Symptom attributions and consequent help-seeking may be influenced by information from radios or healthcare professionals, previous experiences will similar illnesses, approval or sanctioning by lay colleagues, and the onset, nature, persistence and severity of their symptoms as well as their response to medications. We found several interrelated sociocultural and structural barriers to healthcare help-seeking. In order to promote timely help-seeking and early detection of symptomatic breast and cervical cancer, targeted public health messages and campaigns to increase knowledge of women on breast and cervical cancer symptoms and services available need to be coupled with economic empowerment of women and improvement in access to health systems.

Acknowledgement

The authors wish to appreciate the research assistants and field coordinator for their thoroughness in conducting interviews in the midst of challenges including impassable roads and traversing difficult terrains on motorcycles. We are equally indebted to the research participants who freely discussed their ideas on the research questions, thereby providing us

For peer review only - http://bmjopen.bmj.com/site/about/guidelines.xhtml

with detailed information. We also thank the local leaders for their cooperation and participation in mobilizations and guidance for security and safety of research assistants during field visits.

Contributors Study concept and design: JM, FMW and ADM; acquisition of data: ADM and HW; analysis of data: ADM, JH and SS; interpretation of data: all authors; drafting of the manuscript: ADM; critical revision of the manuscript for important intellectual content: all authors; study supervision: JM, FMW, ADM and HW. Final approval of the submitted version of the manuscript and where to submit: all authors.

Funding Research reported in this article was jointly supported by the Cancer Association of South Africa (CANSA), the University of Cape Town and the South African Medical Research Council with funds received from the South African National Department of Health, GlaxoSmithKline (GSK) Africa Non-Communicable Disease Open Lab (via a supporting grant Project Number: 023), the United Kingdom Medical Research Council, MRC (via the Newton Fund). GSK provided in-kind scientific and statistical support as part of capacity strengthening. The funders had no role in study design, data collection and analysis, preparation of the manuscript, decision to publish and where to publish. FMW is Director and SES is co-investigator of the multi-institutional CanTest Collaborative, which is funded by Cancer Research UK (C8640/A23385).

Competing Interests The authors declare that they have no competing interests.

Patient consent Not applicable.

For peer review only - http://bmjopen.bmj.com/site/about/guidelines.xhtml

Ethical approval This study protocol was approved by the Lacor Hospital Institutional Research Ethics Committee (**LHIREC 027/11/2016**). The protocol was thereafter registered and cleared by the Uganda National Council for Science and Technology (UNCST), registration number **HS60ES**. Additional permission to enter into the study communities was obtained from the local leaders at the district and sub-county levels. Written informed consents were obtained form study participants before interviews. Participants received a token of 25,000= (USD 6.8) for light snacks and soft drinks during interviews.

For peer review only

Disclaimers None.

Data sharing statement No additional data are available. Study dataset can be made available to third party following reasonable request to corresponding author.

For peer review only - http://bmjopen.bmj.com/site/about/guidelines.xhtml

References

ASOOGO, C. & DUMA, S. E. 2015a. Factors contributing to late breast cancer presentation for health care amongst women in Kumasi, Ghana. *Curationis,* 38**,** 1287.

ASOOGO, C. & DUMA, S. E. 2015b. Factors contributing to late breast cancer presentation for health care amongst women in Kumasi, Ghana. *Curationis,* 38**,** 1-7.

BEEKEN, R. J., SIMON, A. E., VON WAGNER, C., WHITAKER, K. L. & WARDLE, J. 2011. Cancer fatalism: deterring early presentation and increasing social inequalities? *Cancer Epidemiology Biomarkers & Prevention,* 20**,** 2127-2131.

BIRHANU, Z., ABDISSA, A., BELACHEW, T., DERIBEW, A., SEGNI, H., TSU, V., MULHOLLAND, K. & RUSSELL, F. M. 2012. Health seeking behavior for cervical cancer in Ethiopia: a qualitative study. *Int J Equity Health,* 11**,** 83.

BONSU, A. B. & NCAMA, B. P. 2019. Recognizing and appraising symptoms of breast cancer as a reason for delayed presentation in Ghanaian women: A qualitative study. *PLOS ONE,* 14**,** e0208773.

BRAY, F., FERLAY, J., SOERJOMATARAM, I., SIEGEL, R. L., TORRE, L. A. & JEMAL, A. 2018. Global cancer statistics 2018: GLOBOCAN estimates of incidence and mortality worldwide for 36 cancers in 185 countries. *CA Cancer J Clin.*

BRINTON, L., FIGUEROA, J., ADJEI, E., ANSONG, D., BIRITWUM, R., EDUSEI, L., NYARKO, K. M., WIAFE, S., YARNEY, J., ADDAI, B. W., AWUAH, B., CLEGG-LAMPTEY, J. N., ADJEI, A., DEDEY, F., VANDERPUYE, V., OKYNE, V., OTI, N. O., TAY, E., ADU, A., KENU, A., EKPEDZOR, O., AITPILLAH, F., OPPONG, J., OSEI-BONSU, E., TITILOYE, N., BOAKYE, I., ARHIN, B., ASSIMAH, E., KA-CHUNGU, S., FREMPONG, M., ABAIDOO, E. B., MENSAH, B. N., AMANAMA, S., AGYUAPONG, P., BOATENG-ANSONG, D., AGYEI, T., OPOKU, R., GYIMAH, K. O., HEWITT, S., PALAKAL, M., THISTLE, J., NEWMAN, L.,

For peer review only - http://bmjopen.bmj.com/site/about/guidelines.xhtml

BROTZMAN, M., NIWA, S., SINGH, U. & ON BEHALF OF THE GHANA BREAST HEALTH STUDY, T. 2017. Factors contributing to delays in diagnosis of breast cancers in Ghana, West Africa. *Breast Cancer Research and Treatment,* 162**,** 105-114.

DREWS, C. D. & GREELAND, S. 1990. The Impact of Differential Recall on the Results of Case-Control Studies. *International Journal of Epidemiology,* 19**,** 1107-1112.

DUNYO, P., EFFAH, K. & UDOFIA, E. A. 2018. Factors associated with late presentation of cervical cancer cases at a district hospital: a retrospective study. *BMC Public Health,* 18**,** 1156.

EMERY, J. D., WALTER, F. M., GRAY, V., SINCLAIR, C., HOWTING, D., BULSARA, M., BULSARA, C., WEBSTER, A., AURET, K., SAUNDERS, C., NOWAK, A. & HOLMAN, C. D. 2013. Diagnosing cancer in the bush: a mixed-methods study of symptom appraisal and help-seeking behaviour in people with cancer from rural Western Australia. *Fam Pract*.

FRANCIS, J. J., JOHNSTON, M., ROBERTSON, C., GLIDEWELL, L., ENTWISTLE, V., ECCLES, M. P. & GRIMSHAW, J. M. 2010. What is an adequate sample size? Operationalising data saturation for theory-based interview studies. *Psychol Health,* 25**,** 1229-45.

GEBREMARIAM, A., ADDISSIE, A., WORKU, A., ASSEFA, M., PACE, L. E., KANTELHARDT, E. J. & JEMAL, A. 2019. Time intervals experienced between first symptom recognition and pathologic diagnosis of breast cancer in Addis Ababa, Ethiopia: a cross-sectional study. *BMJ Open,* 9**,** e032228.

GONDOS, A., BRENNER, H., WABINGA, H. & PARKIN, D. M. 2005. Cancer survival in Kampala, Uganda. *Br J Cancer,* 92**,** 1808-1812.

For peer review only - http://bmjopen.bmj.com/site/about/guidelines.xhtml

GONDOS, A., CHOKUNONGA, E., BRENNER, H., PARKIN, D. M., SANKILA, R., BOROK, M. Z., CHIRENJE, Z. M., NYAKABAU, A. M. & BASSETT, M. T. 2004. Cancer survival in a southern African urban population. *Int J Cancer,* 112**,** 860-4.

GUEST, G., BUNCE, A. & JOHNSON, L. 2006. How Many Interviews Are Enough?: An Experiment with Data Saturation and Variability. *Field Methods,* 18**,** 59-82.

IBRAHIM, N. A. & OLUDARA, M. A. 2012. Socio-demographic factors and reasons associated with delay in breast cancer presentation: A study in Nigerian women. *The Breast,* 21**,** 416-418.

JOFFE, M., AYENI, O., NORRIS, S. A., MCCORMACK, V. A., RUFF, P., DAS, I., NEUGUT, A. I., JACOBSON, J. S. & CUBASCH, H. 2018. Barriers to early presentation of breast cancer among women in Soweto, South Africa. *PLOS ONE,* 13**,** e0192071.

JOKO-FRU, W. Y., MIRANDA-FILHO, A., SOERJOMATARAM, I., EGUE, M., AKELE-AKPO, M.-T., N'DA, G., ASSEFA, M., BUZIBA, N., KORIR, A., KAMATE, B., TRAORE, C., MANRAJ, S., LORENZONI, C., CARRILHO, C., HANSEN, R., FINESSE, A., SOMDYALA, N., WABINGA, H., CHINGONZOH, T., BOROK, M., CHOKUNONGA, E., LIU, B., KANTELHARDT, E., MCGALE, P. & PARKIN, D. M. 2019. Breast cancer survival in sub-Saharan Africa by age, stage at diagnosis and human development index: A population-based registry study. *International journal of cancer*.

KAZAURA, M. R., KOMBE, D., YUMA, S., MTIRO, H. & MLAWA, G. 2007. Health seeking behavior among cancer patients attending Ocean Road Cancer Institute, Tanzania. *East Afr J Public Health,* 4**,** 19-22.

KOHLER, R. E., GOPAL, S., MILLER, A. R., LEE, C. N., REEVE, B. B., WEINER, B. J. & WHEELER, S. B. 2017. A framework for improving early detection of breast cancer in

For peer review only - http://bmjopen.bmj.com/site/about/guidelines.xhtml

sub-Saharan Africa: A qualitative study of help-seeking behaviors among Malawian women. *Patient education and counseling,* 100**,** 167-173.

KOPEC, J. A. & ESDAILE, J. M. 1990. Bias in case-control studies. A review. *Journal of epidemiology and community health,* 44**,** 179-186.

MABULA, J. B., MCHEMBE, M. D., CHALYA, P. L., GIITI, G., CHANDIKA, A. B., RAMBAU, P. F., MASALU, N. & GILYOMA, J. M. 2012. Stage at diagnosis, clinicopathological and treatment patterns of breast cancer at Bugando Medical Centre in north-western Tanzania. *Tanzania Journal of Health Research,* 14.

MACLEOD, U., MITCHELL, E. D., BURGESS, C., MACDONALD, S. & RAMIREZ, A. J. 2009. Risk factors for delayed presentation and referral of symptomatic cancer: evidence for common cancers. *Br J Cancer,* 101 Suppl 2**,** S92-S101.

MOFPED 2014. POVERTY STATUS REPORT 2014. Structural change and poverty reduction in Uganda. *Available at: https://www.tralac.org/images/docs/6878/uganda-poverty-status-report-november-2014.pdf,* Accessed on 2nd June 2020.

MOH 2006. Ministry of Health (MOH) [Uganda] and ORC Macro. 2006. Uganda HIV/AIDS Sero-behavioural Survey 2004-2005. Calverton, Maryland, USA: Ministry of Health and ORC Macro. *Available at: https://dhsprogram.com/pubs/pdf/AIS2/AIS2.pdf. Accessed 2nd June 2020.*

MOODLEY, J., SCOTT, S. E., MWAKA, A. D. & CONSTANT, D. 2019. Development and validation of the African Women Awareness of CANcer (AWACAN) tool for breast and cervical cancer. 14**,** e0220545.

MWAKA, A. D., GARIMOI, C. O., WERE, E. M., ROLAND, M., WABINGA, H. & LYRATZOPOULOS, G. 2016. Social, demographic and healthcare factors associated with stage at diagnosis of cervical cancer: cross-sectional study in a tertiary hospital in Northern Uganda. *BMJ Open,* 6.

For peer review only - http://bmjopen.bmj.com/site/about/guidelines.xhtml

MWAKA, A. D., OKELLO, E. S. & ORACH, C. G. 2015a. Barriers to biomedical care and use of traditional medicines for treatment of cervical cancer: an exploratory qualitative study in northern Uganda. *Eur J Cancer Care (Engl),* 24**,** 503-513.

MWAKA, A. D., OKELLO, E. S., WABINGA, H. & WALTER, F. M. 2015b. Symptomatic presentation with cervical cancer in Uganda: a qualitative study assessing the pathways to diagnosis in a low-income country. *BMC Womens Health,* 15**,** 15.

MWAKA, A. D., WABINGA, H. R. & MAYANJA-KIZZA, H. 2013. Mind the gaps: a qualitative study of perceptions of healthcare professionals on challenges and proposed remedies for cervical cancer help-seeking in post conflict northern Uganda. *BMC Fam Pract,* 14**,** 193.

NIKSIC, M., RACHET, B., DUFFY, S. W., QUARESMA, M., MØLLER, H. & FORBES, L. J. L. 2016. Is cancer survival associated with cancer symptom awareness and barriers to seeking medical help in England? An ecological study. *British Journal of Cancer,* 115**,** 876-886.

PACE, L. E., MPUNGA, T., HATEGEKIMANA, V., DUSENGIMANA, J.-M. V., HABINEZA, H., BIGIRIMANA, J. B., MUTUMBIRA, C., MPANUMUSINGO, E., NGIRUWERA, J. P., TAPELA, N., AMOROSO, C., SHULMAN, L. N. & KEATING, N. L. 2015. Delays in Breast Cancer Presentation and Diagnosis at Two Rural Cancer Referral Centers in Rwanda. *Oncologist,* 20**,** 780-788.

PATEL, S., SCHECHTER, M. T., SEWANKAMBO, N. K., ATIM, S., KIWANUKA, N. & SPITTAL, P. M. 2014. Lost in Transition: HIV Prevalence and Correlates of Infection among Young People Living in Post-Emergency Phase Transit Camps in Gulu District, Northern Uganda. *PLoS One,* 9**,** e89786.

POWE, B. D. 1995. Fatalism among elderly African Americans: Effects on colorectal cancer screening. *Cancer Nurs,* 18**,** 385-392.

For peer review only - http://bmjopen.bmj.com/site/about/guidelines.xhtml

SANKARANARAYANAN, R., SWAMINATHAN, R. & LUCAS, E. 2011. *Cancer survival in Africa, Asia, the Caribbean and Central America*, International Agency for Research on Cancer Lyon.

SCOTT, S. E., WALTER, F. M., WEBSTER, A., SUTTON, S. & EMERY, J. 2013. The model of pathways to treatment: conceptualization and integration with existing theory. *Br J Health Psychol,* 18**,** 45-65.

SIMON, A., WALLER, J., ROBB, K. & WARDLE, J. 2010. Patient Delay in Presentation of Possible Cancer Symptoms: The Contribution of Knowledge and Attitudes in a Population Sample from the United Kingdom. *Cancer Epidemiol Biomarkers Prev,* 19.

VAN SCHALKWYK, S. L., MAREE, J. E. & WRIGHT, S. C. 2008. Cervical cancer: the route from signs and symptoms to treatment in South Africa. *Reprod Health Matters,* 16**,** 9-17.

WABINGA, H., RAMANAKUMAR, A. V., BANURA, C., LUWAGA, A., NAMBOOZE, S. & PARKIN, D. M. 2003. Survival of cervix cancer patients in Kampala, Uganda: 1995-1997. *Br J Cancer,* 89**,** 65-9.

WALTER, F., WEBSTER, A., SCOTT, S. & EMERY, J. 2012. The Andersen Model of Total Patient Delay: a systematic review of its application in cancer diagnosis. *J Health Serv Res Policy,* 17.

WERE, E. O. & BUZIBA, N. G. 2001. Presentation and health care seeking behaviour of patients with cervical cancer seen at Moi Teaching and Referral Hospital, Eldoret, Kenya. *East Afr Med J,* 78**,** 55-59.

For peer review only - http://bmjopen.bmj.com/site/about/guidelines.xhtml

In-depth Interview Guide

Understanding symptoms appraisal, help seeking and perceived barriers to healthcare seeking among women with potential symptoms of breast and cervical cancer in Uganda

Introduction

Thank you for accepting to participate in this study. The study is about understanding the way women with symptoms that could be due to breast or cervical cancer explain their symptoms and seek help for the them. In particular, we would like to understand the things that you consider when you are want to explain such symptoms as breast pain, swelling in the breast and discharge from the genitals o a woman. We also need to understand from you the factors that influence the way you seek for help.

To achieve our objective, we shall conduct an interview with you based on questions in our study guide. This interview will take about 45 minutes to one and half hours. Please keep in mind that there are no right or wrong answers to these questions. If I raise an issue or ask a question you don't want to talk about, just say so and we will move on to something else. Plase be free to ask any questions you will like to ask to help you undertand our interview. If you have any questions about the study, you can ask them below starting he interview or afterwards.

Note that you have been invited to participate in this interview because you have indicated that you have had some symptoms including pain and welling in the breasts, and discharge from your genitals during our earlier study. We are very grateful to have participate in this segment of the study.

Guiding questions

A: Detection and appraisal of bodily changes

1. **Thank you for agreeing to be interviewed today. I would like to start by asking you some questions around the changes/symptoms you mention related to your … (here relate to specific symptom mentioned e.g. breast lump, vaginal discharge).**
   Could you tell me more about this change?
   Probes:
   - What was the first thing that you noticed?
   - When did you notice the symptom? Use calendar prompt to assist, based on important landmarks e.g. Christmas, Easter, public holidays etc.
   - Has the symptom changed since you first noticed it? If yes, how has this changed?

2. **Could you describe how you felt when you noticed this symptom (refer to change/symptom e.g. finding a breast lump, vaginal bleeding)?**
   Probe:
   - Emotional response to symptom? E.g. Afraid, anxious, embarrassed, not concerned, denial, concerned that it was serious
   - Why did you feel that way?

For peer review only - http://bmjopen.bmj.com/site/about/guidelines.xhtml

3. **When you first noticed the symptom(s), what did you think was causing the symptom? What made you think that?**
   Probe:
   - Did you think it meant you had any particular illness? If so what illness and what made you think this way?
   - At any point did you think it is/was an infection? Why did you think this?
   - Did you think it was something minor/nothing to worry about? If so, what made you think this?
   - Were you unsure about what it meant? If so what made you unsure?

4. **Since then, have you had any other thoughts about what it might be? (if changed thoughts of what the symptoms could be caused by then, probe reasons for change. E.g. what made you think that?)**

5. **Have you had a symptom like this before? If yes Probe when? What did you do? What was the outcome?**

6. **Do you know of anyone who had a similar symptom? [say the symptom]**

   If Yes, what was the cause of their symptom? What did they do about it? What was the outcome?

7. **Have you ever heard about this symptom/change anywhere? If yes where?**
   Probe: at the clinic, discussed with my family/friends, pamphlets, social media/ posters taxis or trains/ radio/television? What information did each of these sources provide about such a symptom?

8. **Did you discuss this current change/symptom with anyone?**
   Probe:
   If Yes
   - Whom?
   - What did this person/s say? Was it helpful?
   - Did it influence your views on the change/symptom? If Yes how?
   - Why did you discuss with that person (name/relation)?

   If No
   - Why did you not discuss with anyone? Probes feelings of anxiety, not worried about symptom etc.

9. **How do you think people in your community view someone with this symptom (refer to symptoms participant named)**
   Probe:
   - Is there any stigma attached to having this symptom?
   - Are people supportive/empathetic with people with these kinds of symptoms in your community?

For peer review only - http://bmjopen.bmj.com/site/about/guidelines.xhtml

- Is this symptom generally discussed by family and friends?

10. Has this symptom affected your life in anyway?

Probe:
If yes, how has it affected your life?

11. Have you tried to manage the symptoms yourself? Did you try anything to help relieve / resolve the symptoms?

B: Help-seeking behaviour and estimated dates of symptoms

12. Have you gone to have this change/symptom (name symptom) checked by anyone?
If YES
Probes:

- Who did you visit?
- Why did you choose to go there? Could you tell me **what** made you decide to seek care? Was there anything in particular that made you decide to seek assistance?
- When did you go to have it checked? *Use calendar prompt*
- Can you tell me about your experience seeking advice or assistance from …

Have you had the symptom checked by anyone else? (Repeat prompts above)

- Have you visited a traditional healer? If visited traditional healer, was it after or before visiting the health facilities and why before or after (rationale)? What did the traditional healer say about the symptom? *Note participant might discuss this in earlier response to question 12.*

- Have you visited a health care facility or health care provider? If yes when did you visit health care provider? Probe only if person waited to see health care provider - Is there anything that made you wait before getting checked by a health care provider?

Note participant might discuss this in earlier response to question 12.

If NO (woman has not gone to seek care)

- Have you thought about getting checked?
- Do you intend to seek care? If so, from whom? When?
- Are there any reasons for not seeking care from a healthcare provider?
- Is there anything that has stopped you from seeking care?
  e.g. lack of pain, past history of this symptom, belief that it might go away, think/thought it is/was minor, partner, family or friends
- Access - Are there any problems getting to the clinic?
  - Is the cost of getting to a clinic or health care provider a problem?

For peer review only - http://bmjopen.bmj.com/site/about/guidelines.xhtml

- If person working is it a problem to take time off work?
- If person looking after children is it a problem to get to the clinic because you are looking after children

Is there anything that would make you get checked by a healthcare provider?

We are now coming to the end of our discussion. Can I take a minute to pause to see if there are any questions I might have missed?

13. Do you have any other comments, or questions that you would like to ask?

For peer review only

Thank you for participating in this study. Your views are very important to us.

For peer review only - http://bmjopen.bmj.com/site/about/guidelines.xhtml

Checklist: **Symptom appraisal, help seeking and perceived barriers to healthcare seeking in Uganda: An exploratory study among women with potential symptoms of breast and cervical cancer**

		Reporting Item	Page Number
**Title**			
	#1	Concise description of the nature and topic of the study identifying the study as qualitative or indicating the approach (e.g. ethnography, grounded theory) or data collection methods (e.g. interview, focus group) is recommended	1
**1Abstract**			
	#2	Summary of the key elements of the study using the abstract format of the intended publication; typically includes background, purpose, methods, results and conclusions	2
**Introduction**			
Problem formulation	#3	Description and significance of the problem / phenomenon studied: review of relevant theory and empirical work; problem statement	4 - 5
Purpose or research question	#4	Purpose of the study and specific objectives or questions	6
**Methods**			
Qualitative approach and research paradigm	#5	Qualitative approach (e.g. ethnography, grounded theory, case study, phenomenology, narrative research) and guiding theory if appropriate; identifying the research paradigm (e.g. postpositivist, constructivist / interpretivist) is also recommended; rationale. The rationale should briefly discuss the justification for choosing that theory, approach, method or technique rather than other options available; the assumptions and limitations implicit in those choices and how those choices influence study conclusions and transferability. As appropriate the rationale for several items might be discussed together.	6

For peer review only

Researcher characteristics and reflexivity	#6	Researchers' characteristics that may influence the research, including personal attributes, qualifications / experience, relationship with participants, assumptions and / or presuppositions; potential or actual interaction between researchers' characteristics and the research questions, approach, methods, results and / or transferability	N/A
Context	#7	Setting / site and salient contextual factors; rationale	6
Sampling strategy	#8	How and why research participants, documents, or events were selected; criteria for deciding when no further sampling was necessary (e.g. sampling saturation); rationale	7
Ethical issues pertaining to human subjects	#9	Documentation of approval by an appropriate ethics review board and participant consent, or explanation for lack thereof; other confidentiality and data security issues	35
Data collection methods	#10	Types of data collected; details of data collection procedures including (as appropriate) start and stop dates of data collection and analysis, iterative process, triangulation of sources / methods, and modification of procedures in response to evolving study findings; rationale	7
Data collection instruments and technologies	#11	Description of instruments (e.g. interview guides, questionnaires) and devices (e.g. audio recorders) used for data collection; if / how the instruments(s) changed over the course of the study	7
Units of study	#12	Number and relevant characteristics of participants, documents, or events included in the study; level of participation (could be reported in results)	7
Data processing	#13	Methods for processing data prior to and during analysis, including transcription, data entry, data management and security, verification of data integrity, data coding, and anonymisation / deidentification of excerpts	8
Data analysis	#14	Process by which inferences, themes, etc. were identified and developed, including the	8

For peer review only

		researchers involved in data analysis; usually references a specific paradigm or approach; rationale	
Techniques to enhance trustworthiness	#15	Techniques to enhance trustworthiness and credibility of data analysis (e.g. member checking, audit trail, triangulation); rationale	8
**Results/findings**			
Syntheses and interpretation	#16	Main findings (e.g. interpretations, inferences, and themes); might include development of a theory or model, or integration with prior research or theory	9
Links to empirical data	#17	Evidence (e.g. quotes, field notes, text excerpts, photographs) to substantiate analytic findings	9 - 28
**Discussion**			
Integration with prior work, implications, transferability and contribution(s) to the field	#18	Short summary of main findings; explanation of how findings and conclusions connect to, support, elaborate on, or challenge conclusions of earlier scholarship; discussion of scope of application / generalizability; identification of unique contributions(s) to scholarship in a discipline or field	29 - 32
Limitations	#19	Trustworthiness and limitations of findings	32 - 33
**Other**			
Conflicts of interest	#20	Potential sources of influence of perceived influence on study conduct and conclusions; how these were managed	34
Funding	#21	Sources of funding and other support; role of funders in data collection, interpretation and reporting	34

For peer review only

BMJ Open

Symptom appraisal, help seeking and perceived barriers to healthcare seeking in Uganda: An exploratory study among women with potential symptoms of breast and cervical cancer

Journal:	*BMJ Open*
Manuscript ID	bmjopen-2020-041365.R1
Article Type:	Original research
Date Submitted by the Author:	01-Dec-2020
Complete List of Authors:	Mwaka, Amos; Makerere University College of Health Sciences, Medicine<br>Walter, Fiona; Cambridge University, Dept of Public Health and Primary Care; University of Melbourne VCCC<br>Scott, Suzanne; King's College London, Unit of Social & Behavioural Sciences<br>Harries, Jane; University of Cape Town Faculty of Health Sciences, Women's Health Research Unit<br>Wabinga, Henry; Makerere University College of Health Sciences, Medicine<br>Moodley, Jennifer; University of Cape Town Faculty of Health Sciences, Faculty of Health Sciences
**Primary Subject Heading**:	Global health
Secondary Subject Heading:	Oncology
Keywords:	Breast tumours < ONCOLOGY, Gynaecological oncology < ONCOLOGY, PRIMARY CARE

SCHOLARONE™
Manuscripts

I, the Submitting Author has the right to grant and does grant on behalf of all authors of the Work (as defined in the below author licence), an exclusive licence and/or a non-exclusive licence for contributions from authors who are: i) UK Crown employees; ii) where BMJ has agreed a CC-BY licence shall apply, and/or iii) in accordance with the terms applicable for US Federal Government officers or employees acting as part of their official duties; on a worldwide, perpetual, irrevocable, royalty-free basis to BMJ Publishing Group Ltd ("BMJ") its licensees and where the relevant Journal is co-owned by BMJ to the co-owners of the Journal, to publish the Work in this journal and any other BMJ products and to exploit all rights, as set out in our licence.

The Submitting Author accepts and understands that any supply made under these terms is made by BMJ to the Submitting Author unless you are acting as an employee on behalf of your employer or a postgraduate student of an affiliated institution which is paying any applicable article publishing charge ("APC") for Open Access articles. Where the Submitting Author wishes to make the Work available on an Open Access basis (and intends to pay the relevant APC), the terms of reuse of such Open Access shall be governed by a Creative Commons licence – details of these licences and which Creative Commons licence will apply to this Work are set out in our licence referred to above.

Other than as permitted in any relevant BMJ Author's Self Archiving Policies, I confirm this Work has not been accepted for publication elsewhere, is not being considered for publication elsewhere and does not duplicate material already published. I confirm all authors consent to publication of this Work and authorise the granting of this licence.

For peer review only

Symptom appraisal, help seeking and perceived barriers to healthcare seeking in Uganda: An exploratory study among women with potential symptoms of breast and cervical cancer

Amos Deogratius Mwaka[1], Fiona M. Walter[2]*, Suzanne E. Scott[3], Jane Harries[4], Henry Wabinga[5], Jennifer Moodley[4, 6, 7]

Authors' affiliations

1. Department of Medicine, School of Medicine, College of Health Sciences, Makerere University. P.O Box 7072, Kampala, Uganda.
2. The Primary Care Unit, Department of Public Health & Primary Care, University of Cambridge, UK.
3. Faculty of Dentistry, Oral & Craniofacial Sciences, King's College London, UK.
4. Women's Health Research Unit, School of Public Health and Family Medicine, Faculty of Health Sciences, University of Cape Town; Anzio Road. Observatory, 7925, Cape, Town, South Africa.
5. Department of Pathology, School of Biomedical Sciences, College of Health Sciences, Makerere University. P.O Box 7072, Kampala, Uganda.
6. Cancer Research Initiative, Faculty of Health Sciences, University of Cape Town; Anzio Road, Observatory, 7925, Cape, Town, South Africa.
7. SAMRC Gynaecology Cancer Research Centre, Faculty of Health Sciences, University of Cape Town, Anzio Road. Observatory, 7925, Cape Town, South Africa. ORCID 0000-0002-9398-5202

Emails:

Amos Deogratius Mwaka: mgratius@gmail.com

Fiona M. Walter: fmw22@medschl.cam.ac.uk

Suzanne Scott: suzanne.scott@kcl.ac.uk

Jane Harries: jane.harries@uct.ac.za

Henry Wabinga: hwabinga@gmail.com

Jennifer Moodley: jennifer.moodley@uct.ac.za

*Corresponding author: Fiona M. Walter. The Primary Care Unit, Department of Public Health & Primary Care, University of Cambridge, UK. Email: fmw22@medschl.cam.ac.uk

Word count: 7, 598.

For peer review only

Abstract

Objective: We assessed the process of recognising abnormal bodily changes, interpretations and attributions, and help-seeking behaviour among community-based Ugandan women with possible symptoms of breast and cervical cancer, in order to inform health interventions aiming to promote timely detection and diagnosis of cancer.

Design: Qualitative in-depth interviews.

Setting: Rural and urban communities in Uganda

Participants: Women who participated in the African Women Awareness of CANcer (AWACAN) cross-sectional survey who disclosed potential breast and cervical cancer symptoms were eligible; recruitment was purposive. Interviews were conducted in women's homes, lasted between 40 and 90 minutes, were audio-recorded, transcribed verbatim, and translated to English. Thematic analysis was used to identify themes and subthemes, underpinned by the conceptual framework of the Model of Pathways to Treatment.

Results: 23 women were interviewed: 10 had potential symptoms of breast cancer and 13 of cervical cancer. Themes regarding symptom appraisal and help-seeking included the; (i) detection and interpretation of abnormal bodily sensations; (ii) lay consultations regarding bodily changes; (iii) iterative process of inferring and attributing illnesses to the bodily changes; (iv) restricted disclosure of symptoms to lay people due to concerns about privacy and fear of stigmatization; (v) help-seeking from multiple sources including both traditional and biomedical health practitioners, and (vi) multiple perceived barriers to help-seeking including long waiting times, lack of medicines, absenteeism of healthcare professionals, and lack of money for transport and medical bills.

Conclusion: Women with potential symptoms of breast and cervical cancer undergo complex processes of symptom interpretation, attributing symptoms or inferring illness, and lay consultations before undertaking help-seeking and management. Increasing community

For peer review only - http://bmjopen.bmj.com/site/about/guidelines.xhtml

understanding of breast and cervical cancer symptoms, and tackling perceived barriers to health-seeking, could lead to prompt and appropriate symptom appraisal and help-seeking, and contribute to improving cancer outcomes.

Key words: Breast cancer, cervical cancer, Symptoms appraisal and attributions, help-seeking

Strengths and limitations of this study

- To our knowledge, this is the first study set in Uganda to explore the process of recognition of abnormal bodily changes, interpretations and attributions, and help-seeking behaviour among community-based women with possible symptoms of breast or cervical cancer.
- The qualitative nature of the inquiry and strong theoretical underpinning with the Model of Pathways to Treatment enabled in-depth exploration of the research question.
- Conducting interviews by female research assistants in the home of the participants allowed for freedom of expression and in-depth responses by the majority of participants.
- Self-report of symptoms and help-seeking is subject to social desirability bias especially when participants consider that their actual perceptions, beliefs and conducts could attract unfavourable comments from the research team or other people.

For peer review only - http://bmjopen.bmj.com/site/about/guidelines.xhtml

Background

In low- and middle-income countries (LMICs), including most sub Saharan African (SSA) countries, breast and cervical cancers are the most common cancers among women. The incidence of cervical cancer in Eastern and Southern Africa are 40.1 and 43.1 per 100, 000 women respectively, while that of breast cancer are 29.9 and 46.2 per 100, 000 women respectively.(1) In these regions, patients with breast and cervical cancer are often diagnosed in advanced stages.(2-4) In Uganda and Ghana, 66% of cervical cancer patients were diagnosed in advanced stages.(4, 5) In Rwanda, 76% of breast cancer patients were diagnosed in advanced stages.(3) A recent registry-based study in SSA showed that more than half (64.9%) of patients with breast cancer are diagnosed in advanced stages and experience low survival.(6) Advanced stage cancer at diagnosis is associated with poorer survival.(7-10) In SSA, breast cancer overall relative survival at one year and five years were 86.1% (84.4–87.6), and 59.0% (56.3–61.6) respectively. Relative survival varied between registries but ranged from 21.6% (8.2 – 39.8) at three years in Malawi to 84.5% (70.6 – 93.5) in Namibia.(6)

Poor recognition of cancer symptoms by both patients and primary healthcare professionals may contribute to the advanced stage at diagnosis and poor survival of breast and cervical cancer in SSA.(11-14) Presentation at the healthcare facilities and eventual diagnosis of cancer is often long. In Ethiopia and Nigeria, 36% and 81.6% of patients with breast cancer spent more than 90 days with symptoms before seeking care with a healthcare professionals.(15, 16) In Ghana, use of traditional and complementary medicines were associated with prolonged time to help seeking.(17, 18) Several patient factors including individual, socio-cultural and health system factors interact to influence the patients' pathway to treatment for their cancer symptoms.(19, 20) In general, patients' awareness of cancer symptoms has been shown to be associated with cancer survival.(21) Similarly, in South Africa, breast cancer patients with

For peer review only

higher education attainment and with greater awareness and knowledge of breast cancer symptoms were statistically significantly more likely to be diagnosed with early stage disease.(22) In addition, self-management, use of traditional medicines and perceived multiple barriers to medical care have been associated with longer time to healthcare seeking for breast and cervical cancer symptoms in SSA.(3, 23-25) In the USA, cancer fatalism among African Americans and Hispanics was associated with advanced stage at diagnosis, inadequate uptake of treatment, and less prompt healthcare seeking for cancer symptoms.(26, 27) However, most of these studies have involved hypothetical help-seeking or retrospective accounts of those already diagnosed with cancer.(12, 13, 28) Hypothetical help-seeking reported by people without real symptoms may not be similar to real help-seeking decisions because of somatic and emotional experiences. On the other hand retrospective recall of symptoms and decision making processes could be enhanced or minimized because of the cancer diagnosis.(29, 30) In addition, help-seeking accounts of patients already diagnosed with cancers are likely to be influenced by their cancer diagnoses and multiple interactions with healthcare professionals. This may not be the case in symptomatic women without a cancer diagnosis and in the community.

Understanding factors influencing appraisal and help-seeking decisions for women with possible symptoms of breast and cervical cancer is a critical step to underpin the development of interventions to promote prompt recognition of symptoms and healthcare help-seeking. In this study set in Uganda, we sought to understand the processes of symptom recognition, appraisal and attribution, and perceived barriers to healthcare seeking among community women with recent experiences of possible symptoms of breast and cervical cancer, regardless of whether they had sought help or been diagnosed with cancer, in order to inform the design

For peer review only - http://bmjopen.bmj.com/site/about/guidelines.xhtml

of interventions to promote prompt symptom appraisal and healthcare help-seeking for women in SSA.

Methods

Design

A qualitative in-depth interview study.

Study setting

The study was conducted across two sites in northern Uganda: an urban site in Gulu district and a rural site in Nwoya district. Most people in northern Uganda earn their livelihood from subsistence agricultural practices; there are low levels of education and chronic intergenerational poverty in the region. Although the level of poverty has decreased since the return of peace in 2006, the region still remains the poorest in Uganda. Greater proportions of households in the region are larger in size and less likely to have two meals a day than in the rest of Uganda.(31) HIV infection has consistently remained higher in this region compared to other parts of Uganda.(32, 33)

Study population and recruitment

This interview study was part of a larger study involving a cross-sectional survey using the AWACAN tool.(34, 35) Eligibility criteria for this study included: women aged 18 years and over, self-reported experience of symptoms suggestive of possible breast or cervical cancer within the past three months, who had participated in the survey, spoke English or the Luo/Acholi language, and were willing to provide written consent to participate in an interview. Participants also needed to report two or more possible symptoms of breast or cervical cancer, but not have had a breast or cervical cancer diagnoses.

For peer review only - http://bmjopen.bmj.com/site/about/guidelines.xhtml

In the urban site, 41 potentially eligible women were identified via the survey, but 15 did not meet inclusion criteria. 14 women were purposively selected to include a range of symptoms and age. In the rural site, 25 AWACAN survey participants reported symptoms suggestive of breast or cervical cancer, but 11 did not meet inclusion criteria. All the remaining 14 were considered for interviews. Interviews were conducted during October 2018 in the urban site and January - February 2019 in the rural site; they continued until data saturation was reached in both localities.(36, 37)

Data collection

Two female research assistants conducted in-depth interviews in both sites. One research assistant led the interviewing process while the other operated the digital audio-recorder and took field notes, especially of non-verbal communication and events during interviews. It was necessary for the research assistants to work in pairs to ensure their personal security as they travelled in the villages. Data collection was mainly conducted in the women's homes, usually in quiet rooms without others present, and occasionally under trees in the family compounds. The research assistants used piloted interview guides, underpinned by the concepts of the Model of Pathways to Treatment.(19, 20) The interview guide included how bodily changes were first recognised, what happened immediately after detection of a symptom, who were consulted about the symptoms, and the steps undertaken to seek help (see Supplementary File 1). Socio demographic data including age, marital status, number of biological children, highest educational attainment, and HIV status were also collected. Each interview lasted between 45 and 90 minutes, and all interviews were audio-recorded.

Data analysis

For peer review only - http://bmjopen.bmj.com/site/about/guidelines.xhtml

For peer review only

Audio-recordings were transcribed verbatim by the research assistants. ADM read transcripts and conducted preliminary analyses during data collection in order to determine data saturation. Before full analyses, ADM reviewed ten transcripts (five from each site) against the recordings to ensure appropriate details were captured and transcripts labelled appropriately. ADM read three transcripts in detail, developed codes, and shared the codebook with the wider study team. Codes were redefined and refined as necessary. Atlas.ti version 6.1 was used to support data analysis. Coded data segments from all transcripts were aggregated and retrieved for detailed analyses. Themes and subthemes were inductively developed from the data while focussing on the research objective and the concepts in the Model of Pathways to Treatment.

Patient and Public Involvement

Patients were not involved in the planning and implementation of this study. However, the public was involved through the district and community leaders. The district health officers and district women representatives on the district councils of the two study districts were involved in the study. These local leaders were part of our Project Advisory Committee (PAC) which we formed to provide advice and guidance to the study team to ensure that the study is conducted in a culturally acceptable manner, and the findings are relevant to the local context in order to inform local and national health policies on targeted interventions to promote cancer awareness and early detection. The study objectives and tools were discussed with these stakeholders. They provided guidance on how our study could refined to best fit the local context. We therefore refined the study tools to capture aspects of women's lives that relate to information seeking and access, healthcare seeking, and decision making in the families. In addition, the local leaders will be involved in disseminations of study findings. Their presence during dissemination is expected to make the population more confident in the results. The

For peer review only - http://bmjopen.bmj.com/site/about/guidelines.xhtml

involvement of the local decision makers potentially increases the chance that this study findings could be acceptable and put into use.

Results

Characteristics of the participants

23 women were interviewed: 10 had possible symptoms of breast cancer and 13 had possible symptoms of cervical cancer. Two self-reported being HIV positive and on antiretroviral therapies. Overall, the median age was 31 years (range: 18 – 66 years; urban site 27 years (range: 22 – 66 years), rural site 41 years (range: 18 – 64 years)), (see **Table 1).**

For peer review only - http://bmjopen.bmj.com/site/about/guidelines.xhtml

Table 1: Characteristics of respondents and reported symptoms

	Study site		Age group (Years)	Marital status	Education attainment	Occupation	Symptoms	HIV status
**Participants**	Urban	Rural						
P1	X		31 - 50	Widow	Primary 5	Unemployed	Pain and lump in breast	Negative
P2	X		31 - 50	Married	Primary 4	Business/trader	Pain and lumps in both breasts	Positive
P3	X		51 - 70	Widow	Primary 6	Business/trader	Pain and lump in breast	Negative
P4	X		18 - 30	Separated	Primary 3	Waitress in restaurant	SVD*, Vaginal bleeding between periods, Persistent LAP*	Negative
P5	X		18 - 30	Married	Senior 4	Business/trader	Pain and lump in breast	Negative
P6	X		18 - 30	Married	Senior 4	Business/trader	Vaginal bleeding during/after sex, Persistent LAP	Negative
P7	X		18 - 30	Married	Tertiary education	Business/trader	Pain and lump in breast	Negative
P8	X		18 - 30	Married	Senior 3	Business/trader	SVD, Persistent LAP, Pain and discomfort during sex	Negative
P9	X		31 - 50	Widow	Primary 3	Business/trader	SVD, Persistent LAP	Negative
P10	X		18 - 30	Married	Primary 7	Unemployed	Lumps in armpits and both breasts	Negative
P11	X		51 - 70	Married	Primary 6	Unemployed	Pain and lumps in both breasts	Unknown
P12		X	51 - 70	Married	No formal education	Unemployed	Lumps in armpits and both breasts	Negative
P13		X	31 - 50	Married	Primary 2	Unemployed	SVD, Persistent LAP, Pain and discomfort during sex	Positive
P14		X	18 - 30	Married	Primary 5	Unemployed	Pain in nipple, lump in breast	Negative
P15		X	31 - 50	Married	Primary 2	Unemployed	Lump and pain in breast, dimpling of breast skin like orange peel	Negative
P16	X		18 - 30	Separated	Primary 6	Unemployed	SVD, Persistent LAP	Negative
P17		X	31 - 50	Married	Primary 6	Business/trader	Persistent LAP, discomfort during sex	Negative
P18		X	31 - 50	Married	No formal education	Unemployed	Persistent LAP, discomfort during sex	Negative

For peer review only - http://bmjopen.bmj.com/site/about/guidelines.xhtml

P19		X	51 - 70	Married	Primary 1	Unemployed	SVD, Persistent LAP, Pain and discomfort during sex	Negative
P20		X	18 - 30	Married	Primary 3	Unemployed	SVD, Vaginal bleeding between periods, Persistent LAP	Negative
P21		X	31 - 50	Married	Primary 3	Unemployed	SVD, Vaginal bleeding between periods, Persistent LAP	Negative
P22		X	18 - 30	Married	Primary 7	Unemployed	Persistent LAP, discomfort during sex	Negative
P23	X		18 - 30	Married	Tertiary education	Accountant	SVD, Persistent LAP, Itching in the vagina	Negative

*LAP = Lower abdominal pain, SVD = Smelly vaginal discharge

For peer review only - http://bmjopen.bmj.com/site/about/guidelines.xhtml

Main themes

Themes regarding symptom appraisal and help-seeking intervals included the; (i) detection and interpretations of abnormal bodily sensations; (ii) lay consultations regarding bodily changes; (iii) iterative process of inferring and attributing illnesses to the bodily changes; (iv) nondisclosure of symptoms to lay people due to concerns about privacy and fear of stigmatization; (v) help-seeking from multiple sources including both traditional and biomedical health practitioners, and (vi) multiple perceived barriers to biomedical care. Supporting quotations are presented followed by participant characteristics (age group and symptoms of cancer type).

(i) Detection and interpretation of abnormal bodily sensations

Detection and initial interpretation of bodily changes as normal or abnormal punctuated participants' thoughts and course of actions regarding their symptoms. The circumstances when bodily changes were first realised were critical to initial interpretations.

Expected bodily changes within a person's lifespan

The age of the woman was critical in deciding whether a certain bodily change could be due to an illness or normal changes. Young women could readily associate bodily changes with menstrual cycles and possibilities of pregnancies, while older women were more concerned with menopausal changes and age-related ailments.

> "That problem, I thought it was my state of menopause that I reached and I stopped giving birth. I thought that way because I wasn't conceiving or giving birth anymore", (**Rural_51-70_Cervical**).

For peer review only - http://bmjopen.bmj.com/site/about/guidelines.xhtml

Participants who had ever breastfed or been pregnant initially perceived their breast symptoms to be due to usual bodily changes, and paid little attention until symptoms persisted or became painful

> "When this lump came up, I thought that probably it is as usual when it would happen when I'm breast feeding. But now I'm not even breast feeding, but the lump on my right armpit is still there and painful", (**Rural_51-70_Breast**).

Chronology with recent events

Bodily changes were interpreted based on circumstances around their onset. For example, a participant who started to experience abnormal bodily changes following a medical procedure or surgery would infer the changes to the procedure.

> "I thought that was an effect from the contraceptive I had inserted in my arm. Before I started using that implant, those symptoms were not there; it was not happening", (**Urban_18-30_Breast**).

Some participants interpreted their bodily changes in respect to their medical histories including post-abortion complications and effect of birth trauma following deliveries.

> "I don't really know [. . .]. I also had a miscarriage with him but my uterus wasn't "cleaned". The doctor said after such an incident one has to undergo "cleaning" of the uterus in order to be healthy once again. So that is why I feel it was that problem causing

For peer review only

For peer review only - http://bmjopen.bmj.com/site/about/guidelines.xhtml

the symptoms [. . .]. I feel that probably there are some remaining dirt in my womb", (**Rural_31-50_Cervical**).

Poor personal hygiene, crowded living environment and risk of infections

Living in crowded unhygienic dwellings especially rented units influenced the initial interpretations of bodily changes experienced. Women considered personal hygiene as critical determinants of gynaecologic symptoms. Women who thought their personal hygiene was compromised were quick to think of hygiene-related causes or infections, especially if they did not have a sexual partner who they could suspect for transmitting a sexually transmitted disease. This view was predominantly held among younger urban women with gynaecologic symptoms.

"I first thought it could be candida . . . because it also causes itching in the vagina. I thought of the fact that there are many tenants here, so the issue of sharing the bathroom could be the problem; you could easily get it", (**Urban_18-30_Cervical**).

"May be poor hygiene, because from school you bathe in a crowded place. You bathe where everybody shares things; you share the buckets. So I think poor hygiene was one of the factors, yeah", (**Urban_18-30_Cervical**).

(ii) Lay consultations regarding bodily changes

Several factors and concerns influenced women to share their symptoms with other people, including husbands, siblings, trusted friends and sometimes parents. Participants engaged in lay consultations for various reasons.

For peer review only - http://bmjopen.bmj.com/site/about/guidelines.xhtml

Seeking financial and practical support

Most participants shared their symptoms with significant others to solicit financial and practical support. These women needed to reach the healthcare facilities for care, but lacked money or means of transport.

> "I thought that being my husband, if I told him he would take me to the health facility. He told me that he would have taken me . . . but he doesn't have the money. I didn't share it with anyone else because nobody else could help me with the money", (**Rural_31-50_Cervical**).

Learn and benefit from experiences of others

Many participants shared their symptoms with other people either because they trusted them or wanted to know more from them to help them decide the course of actions for their symptoms. Some of the confidants were healthcare professionals related to the participants or had had similar symptoms in the past and or were considered to know more about the symptoms.

> "I tried to tell my Mother in-law, and also my sister in-law who had recently returned home, then later I told my husband. I shared with her because I felt she was a mature person and could be having some ideas about it. So she just responded by saying; that definitely needed me to go to the hospital and was not to be tampered with [. . .]. I chose her because I felt she was close to me and we were relating so well", (**Rural_18-30_Breast**).

For peer review only

> "They looked to be more informed that is why I also went to them. One of them told me that she also experienced those symptoms when she was pregnant but now it's not there. That she went to the health facility and was treated, so she was saying that if I also go and get treatment I will feel better", (**Urban_18-30_Cervical**).

Social accountability and avoidance of suspicion from family

A few participants shared with family, especially when the woman found it difficult to accomplish her roles in the family because of her illness.

> "The reason as to why I decided to tell them is because when I have the pain sometimes I need to lie down for a while to rest and calm the pain. But if you don't tell them (in-laws) they may think you are just a lazy woman who doesn't want to do work, so I had to tell them so that they can be aware", (**Urban_18-30_Breast**).

A few participants shared their gynaecologic symptoms with their husbands not just to get financial support to seek care, but rather because they wanted to avoid their husbands suspecting them of engaging in sexual intercourse with other men.

> "I decided to share with him (husband) because I didn't want him to suspect me of having cheated on him and got the infection. So I told him that I had never cheated on him or had these kind of symptoms before; so it was him who had infected me with it", (**Rural_18-30_Cervical**).

(iii) Nondisclosure of symptoms to lay people

For peer review only

While most participants shared their symptoms, most were keen not to share with people they did not trust.

Privacy concerns

Majority of participants considered symptoms of breast and cervical symptoms as private and not appropriate to share with just anyone.

> "By the way sharing your personal issues anyhow with anyone makes you become a fool. For me, once you are mature enough, don't go out sharing your issues anyhow", (**Rural_31-50_Cervical**).

Stigma and gossiping

Some participants who did not share their symptoms were concerned about gossiping and becoming objects of public discussions.

> "Some people like rumour mongering. For example, they can say such a person said this, they could now be having HIV/AIDS. They can talk; that you know, this person said they are in this state and that is already rumour mongering done. So they will be circulating your name. It is because I fear lies of people [. . .]. That is why it is very hard to share with people any secrets, because once you do, news go all over. This is why I no longer like sharing anyhow", (**Rural_51-70_Cervical**).

Self-perceived stigma could be an issue among symptomatic women, especially those with cervical symptoms.

For peer review only - http://bmjopen.bmj.com/site/about/guidelines.xhtml

"As for other people, if you start to share your issues with them, they may judge you without assessing; they will end up airing your issues to people with other funny added lies", (**Rural_31-50_Cervical**).

Low expectations from lay consultations

Some participants had low expectations from lay consultations and therefore preferred formal healthcare seeking as the only and best response to illness symptoms.

"I felt I should go straight to the hospital so that the doctor himself examines me; to me sharing with someone else here wouldn't yield much because they may mention something that will never be real compared with what the doctors will examine and find out", (**Urban_31-50_Cervical**).

(iv) Symptoms attributions: inferring illness

Participants attributed their symptoms to various disorders including infections and cancers. Symptom attributions were guided by the nature of onset, evolution and persistence of symptoms, participants' own previous experiences, family history of illnesses, response to attempted treatments, sanctioning/disapprovals from lay consultations, and messages from the radio or healthcare professionals.

Symptoms could be due to cancer

Gynaecologic symptoms that were unfamiliar and which persisted were often attributed to cervical cancer. These women expressed a lot of worries as they were inferring illness, mostly related to death and orphanhood.

For peer review only - http://bmjopen.bmj.com/site/about/guidelines.xhtml

"I have been asking myself what this could be! It kept me worried a lot. It was worrying me; I used to say that the symptoms I'm experiencing could be cancer and if it is, then that would be too bad. That is what was moving in my mind [. . .]. I just felt that cancer disease is one that can definitely lead to things such as that. I thought so because cancer is a very bad disease, it is really a bad disease . . . it hurries you and you will be short lived compared to other diseases", (**Urban_**31-50**_Cervical**).

Participants who ever screened for cervical cancer considered cancer as possible explanation for their symptoms but were unsure as they had tested negative on earlier screening tests.

"I have been worried about these symptoms and signs. I first thought it was cancer, but I went for cancer screening two years ago, so am still waiting for one more year to go and test again. I thought it could be cancer because cancer is the most dangerous infection, so people fear it most", (**Urban_**18-30**_Cervical**).

"I also thought about cancer but I had already tested and it was negative so they had told me to test again after three months - oh no! They said three years. I gave birth in 2013, so I tested in 2014. They had told me to go back in the previous year, 2018, but I haven't gone back again . . . I thought that probably it is the one starting. Because I heard that when you start experiencing smelly vaginal discharges that could be a sign of cancer. All I thought about was that it could be syphilis, if not, then cancer", (**Rural_**31-50**_Cervical**).

Severity and persistence of symptoms as well as messages from healthcare professionals informed and or reinforced participants' attributions of symptoms to possible cervical cancer.

For peer review only - http://bmjopen.bmj.com/site/about/guidelines.xhtml

For peer review only

> "[My] lower back/abdominal pain made me think it was cervical cancer causing these symptoms. Because the lower abdominal pain was too much. But also I heard from the health facility. They were educating those who had gone to test for that cancer. The healthcare provider said that you can get this cancer from a man who has had sex with a woman who has the virus then he can transmit it to you through sex", (**Rural_18-30_Cervical**).

Persistence of bodily changes beyond ordinary expectations and severity of symptoms caused participants to review their attributions, often considering more serious illnesses including cancers.

> "I thought probably it could be cancer because of the much pain I was experiencing on my breast, or that it was just normal pain resulting from the back pain I sometimes experience. But then sometimes I wonder why one side of my breast has a lump and the other doesn't; so all these leave me in wonder of what could be the problem", (**Urban_31-50_Breast**).

Participants who were aware of breast cancer symptoms immediately attributed the changes experienced in their breasts to possible breast cancer. Most reported that they commonly learn about symptoms of illness from radio programs.

> "The reason as to why I thought it was breast cancer is because I always listen to the radio and these doctors always talk about cancer and how cancer can start and also the

For peer review only - http://bmjopen.bmj.com/site/about/guidelines.xhtml

signs and symptoms of cancer on people. So they tell you when you see such signs, please go to hospital", **(Urban_31-50_Breast)**.

Persistent and relentless pain not responding to commonly used medications or home-based remedies often led participants to think of more serious illnesses including cancers.

"I thought it could be cancerous because of the pain. The pain used not to cool before I went to the hospital; so I thought it was the toughest disease, cancer [. . .]. At least HIV/AIDS, it is easy to get drugs for it, but this cancer has no drug, till you die and get buried; and that is its medicine", (**Urban_51-70_Breast**).

Sexually transmitted infections and other gynaecologic infections

Participants who had symptoms of vaginal itching and discharge readily attributed the cause to candidiasis, believed to be transmitted both through sexual intercourse and poor personal hygiene. Some attributed their bodily changes to sexually transmitted infections, and blamed symptoms on their husbands who were often considered promiscuous.

"I thought that it could be some sexually transmitted infection. I was thinking that it could be gonorrhoea because if it were syphilis, I would have known it because it comes with a lot of pain, and I don't have the smelly vaginal discharge", (**Urban_18-30_Cervical**).

"I thought that probably my husband cheated on me with an infected person because I had never experienced these symptoms. […] ever since he was away for four months from October to February without coming home even one single day, then when he

For peer review only - http://bmjopen.bmj.com/site/about/guidelines.xhtml

comes back after sometimes, I start experiencing these symptoms! So I had every reasons to be suspicious", (**Rural_31-50_Cervical**).

(v) Help-seeking for symptoms

Women sought help from both biomedical and traditional health practitioners including faith-based healers. Participants chose a particular help-seeking route depending on perceived causes, knowledge of previous similar illnesses that responded to a particular treatment approach, advice from social networks, and messages from the media especially local radio health programs.

Healthcare help-seeking

Most participants sought care in healthcare facilities because they needed tests to confirm the causes of their health problems; they felt these would direct them to the appropriate remedies.

"I went to the doctor because I know he is the only person who can help with my condition; they have testing kits and they can confirm without doubts. They have machines that test and confirm the truth. Those things that are just guessed are not helpful at all", (**Urban_31-50_Cervical**).

Most sought care in healthcare facilities whenever symptoms worsened and or caused worries, or when home-based remedies did not lead to improvement in symptoms. Persistent pain was a very common trigger for healthcare seeking. The media, especially radio talk shows, encouraged a significant proportion of participants to seek care at health facilities.

For peer review only - http://bmjopen.bmj.com/site/about/guidelines.xhtml

> "For me the first signs I saw was the swelling of my breast and I warmed water and started massaging on the swollen part but it didn't help, so I decided to go to hospital and told the doctor. Another reason as to why I visited the hospital with the symptoms of my sickness is because of the constant teaching and reminders by doctors over the radio during their radio talk shows that whenever you have any signs and symptoms which is unusual, you should go to hospital. […] I believe if you go to the hospital early enough with any sickness the doctors will find the name of the sickness and cure you if possible", (**Urban_31-50_Breast**).

> "I went to the clinic, bought medicine and took it. But there was no improvement that's why I decided to go to the hospital. It was in March this year (2018) that I went to the clinic; but this pain started after giving birth. What made me go to the hospital was because I was worried. I was worried that I could be having a sexually transmitted disease or cervical cancer", (**Urban_18-30_Cervical**).

Advice from healthcare professionals when women go for antenatal and postnatal care, and immunization for their children motivated them to seek care whenever they experienced symptoms of illnesses.

> "They say it; doctors always talk when we take our children for immunization. Those responsible for the hygiene and sanitation of the hospital once you take your children for immunization or antenatal care, they often tell us that once we get to see any funny signs of any sickness, we need to rush to the nearest health facility early enough. Otherwise the symptoms will worsen, because if you feel pain in your body, you need

For peer review only - http://bmjopen.bmj.com/site/about/guidelines.xhtml

to go to the hospital. So I took that up and rushed to the hospital because I was also feeling that in my body", (**Rural_31-50_Cervical**).

Participants were also motivated to seek biomedical care for concerns regarding the welfare of their children. They would want to remain alive and nurture their children their own way.

"The reason why I decided to go to the hospital that time when I was feeling the symptoms of my lower abdomen and the waist, is because I decided that I should stay healthy . . . to care for my children. I should surely remain healthy and keep them", (**Rural_31-50_Cervical**).

Perceived inconveniences to others and personal discomfort due to symptoms was another factor that triggered healthcare seeking among participants. Symptoms of cervical cancer including smelly vaginal discharge and excessive vaginal bleeding can be very inconveniencing and restrictive to the movement of a woman.

"I went because I was not comfortable among my fellow girls; you could see others are happy and for me I have my hidden problems, so I had to go and solve it with the doctor", (**Urban_18-30_Cervical**).

Traditional and complementary medicines

A few participants sought care from traditional health practitioners including herbalists and faith-based healers who used prayers, sometimes combined with herbal preparations to treat different ailments. Some participants thought traditional and complementary medicines (T&CMs) could be useful while others contended that T&CMs are fake, and that the

For peer review only - http://bmjopen.bmj.com/site/about/guidelines.xhtml

practitioners are dishonest. Most participants thought that T&CMs do not work. They said the medicines are given based on guess work, as there are no tests done to identify cause of illness. These negative views about T&CMs were held by participants from both urban and rural sites, and with both breast and cervical symptoms.

> "I don't even think about it because some usually walk past here hawking herbal drugs claiming they have what treats ulcers, blood pressure, cancer and many more diseases, but I always tell them I don't have money to buy and that I don't use herbs. There is nothing that can attract me in buying local herbs. My opinion is that traditional herbs nowadays don't work or not effective", (**Urban_31-50_Breast**).

> "With traditional medicines, you can be given just anything that will not even help you. So I will never make a commitment to use traditional medicine. They may even just go and pluck the leaves of black jack then they brand it that it is medicine for such and such a disease and yet in actual sense, it will not be the cure for that disease. So most often I stick to my decision of going to the hospital because their medicines will have been tested", (**Urban_31-50_Cervical**).

> "I haven't got any medicine from any traditional healer and I personally don't want. To tell you, these traditional medicines even if you are given in regards to these symptoms in the stomach, they will not work . . . So I really wonder whether the traditional medicines really treat those diseases", (**Rural_31-50_Cervical**).

A few of the participants who admitted to using T&CMs testified that T&CMs, the ones they used, did not work.

For peer review only - http://bmjopen.bmj.com/site/about/guidelines.xhtml

> “I don’t support that, and . . . I will stop T&CM use because I personally tried and it failed to work; it didn’t help me with anything. It is true . . . I was instructed to go get the traditional medicine . . . I first went to that other T&CM practitioner, then I went to the nurse”, (**Urban_18-30_Breast**).

> “I tried taking traditional medicine in vain, and when I tried to use that without success, then my husband told me that its better I try to go to the hospital instead so that it is the doctor to feed me with the right information. I used to dig out the roots then I would drink the juice. No improvement was realized”, (**Rural_51-70_Cervical**).

On the other hand, a substantial minority of participants who had ever used a T&CM reported that they are effective and can be used alongside modern medicine. They felt that when the cause of an illness is due to malevolent spirits or breaching cultural norms, then traditional health practitioners are helpful. Participants from the rural site were more likely to report use of T&CM than those from the urban site.

> “When I used to pass sticky stool and also felt pain in the lower abdomen, the thing that helped me the most was one traditional medicine . . . it was called *tusua* (not real name). That is a type of tree . . . I would dig out the roots then pound it until fine, then I boil for long, then I filter it into the cup, and afterwards when it cools I take it, then it would end up the stickiness of my stool immediately. I also used to have tiny itchy rashes in between my thighs. […] I used to break the leaves of *okala* tree (not real name). You know that tree has some whitish sap, so I would harvest that and place on the spot of

For peer review only - http://bmjopen.bmj.com/site/about/guidelines.xhtml

> the blisters and the sap from *okala* ended up curing, till to date there are no rashes", (**Rural_31-50_Cervical**).

(vi) Perceived barriers to healthcare help-seeking

Participants discussed several barriers to prompt healthcare help-seeking. These included health system challenges including lack of medicines, long waiting time to see healthcare professionals, lack of money, and competing social obligations.

Lack of medicines in the public health facilities

Most participants who contemplated healthcare help-seeking for their symptoms lamented the sorry state of the public healthcare facilities. They reported that there were often no medicines available.

> "I just get discouraged with the thought of going there and being told to buy the recommended drugs from outside", (**Urban_51-70_Breast**).

> "They kept telling me that the drugs they need to inject me with is out of stock! I went back two times and each time the same story, so I never went back again", (**Urban_18-30_Cervical**).

Long waiting times

Due to the long queues and extended waiting times to see the healthcare professionals, especially in the public health facilities, participants often said they would consider alternative avenues to healthcare help-seeking. This view was more common among participants with breast symptoms especially from the urban site.

For peer review only - http://bmjopen.bmj.com/site/about/guidelines.xhtml

> "The main referral hospital also has its own problems; you can take like two days while still on the line to see the doctor. It is equivalent to someone coming all the way from Sudan and going back; that is the line to see the doctor. Whenever you are almost at the front of the line, the doctor leaves. So when you combine all the waiting with hunger and ulcers, you end up giving up", (**Urban_51-70_Breast**).

> "I'm just planning to go in this coming February. [The] hospital is usually very crowded; if you go there you can spend a long time without getting treatment", (**Rural_18-30_Cervical**).

Absence of healthcare professionals at the healthcare facilities

Participants from both urban and rural sites reported visiting healthcare facilities several times but not finding the appropriate healthcare professionals to address their concerns.

> "As you know these government hospitals, services are not like for other private hospitals. So the experience I had was - the first time I went to the hospital, they recommended that I first have a scan done, but the person who was supposed to do it wasn't around so they told me to go back the next day, which I did and they told me the person supposed to do it was still not there so I should go back on Monday, (the following week)", (**Urban_31-50_Breast**).

> "I went and the healthcare provider picked the referral note and put it in the file where they had written my complaints. Then I was told that the doctor isn't there and that I should just go back on Friday, when the doctor will be around and also the ultra-scan

For peer review only - http://bmjopen.bmj.com/site/about/guidelines.xhtml

For peer review only

machine will also be working, so that they can also do an ultra-scan the next Friday", (**Rural_18-30_Cervical**).

Lack of money for healthcare help-seeking

Most participants reported financial constraints to seeking healthcare in the private facilities where there would be no crowding, and medicines available, and described unaffordable fees for consultations, tests and treatments.

"There is really pain, but the main reason why I didn't go is the lack of finances that opens every door", (**Rural_31-50_Breast**).

Participants reported that patients sometimes had to sell whatever they have to get some money to seek care in the private health facilities where they expect reasonable care.

"There is nothing else, because for the three times I visited [the private] hospital, and two times in the government hospital I have been selling goats of which they are now finished. There is nothing more to sell, and yet I have not yet received any improvement", (**Rural_31-50_Breast**).

Rural participants also mentioned competing demands for the little money they got from selling off their belongings to support their medical expenses.

"So when I get money I will seek for help in the health facility but for now it's tricky because I have grown up children and grandchildren who are under my care. So getting the money to go to the health facility is a problem. And as you also know *Lexam* (not real name) hospital, all the services there require money. […] I plan to go to *Lexam*

For peer review only - http://bmjopen.bmj.com/site/about/guidelines.xhtml

hospital because *Takila* regional referral hospital (not real name) is quite far away, *Lexam* is nearer to our home here", (**Rural_51-70_Breast**).

"It is the issue of lack of money that prevented me from going. […] I am struggling so that I raise some money to buy books for my children and enrol them in school then I will think of going to the hospital. I do want to seek help but the means to access that is what is still defeating me. I need, because I also love my body. It is only the issue of money and if I had it today, I would go straight to the hospital", (**Rural_31-50_Cervical**).

Transport money to the public health facilities was another challenge for women, especially those from the rural areas.

"I'm still looking for money to go with to the hospital. I will go when I get the money. The money is for transport. It is 12,000= (USD 3.3) one way, so I need 24,000= (USD 6.6) for transport on a boda boda (*hired motor cycle*)", (**Rural_18-30_Cervical**).

For peer review only - http://bmjopen.bmj.com/site/about/guidelines.xhtml

Discussions

Main findings

This study is one of the first community-based inquiries into symptom recognition, interpretation, appraisal, illness attribution, and consequent help-seeking among women with possible breast and cervical cancer symptoms in urban and rural Uganda. We found that women undergo a complex process of sense-making and help-seeking for their symptoms. The detection and interpretation of abnormal bodily sensations were interpreted based on expected bodily changes within a person's lifespan, perceived poor personal hygiene, and crowded living environments with risk of infections. Upon realising abnormal bodily changes, most participants engaged in extensive lay consultations with relatives, friends and acquaintances mainly because they wanted to solicit financial and practical support, learn and benefit from experiences of others regarding the symptoms, and for social accountability and avoidance of suspicion from husbands/families especially when symptoms were disabling and restricting participations in their daily chores. On the other hand, a substantial minority of participants did not disclose their symptoms to lay colleagues mainly because of low expectations of support, concerns with privacy, avoidance of possibility of gossiping, and concern with stigmatization from the community. Medical pluralism was common, with participants often seeking help for symptoms from both biomedical healthcare facilities and traditional and complementary medicine providers including faith-based healers, despite many being sceptical about the effectiveness of traditional and complementary medicines.

Our findings regarding recognition of abnormal bodily changes, interpretation and symptom attributions, as well as disclosure/nondisclosure of symptoms to lay people, are similar to findings from studies in other SSA countries such as Malawi and Ghana among breast cancer patients.(13, 38) The patients appraised their symptoms within the context of their life events

For peer review only - http://bmjopen.bmj.com/site/about/guidelines.xhtml

and situations, and were guided by expected bodily changes, family history of cancer and suggestions from lay consultations. The attribution of symptoms changed over time depending on characteristics of the symptoms, including their nature, evolution, response/nonresponse to common medications, and advice from lay people. Understanding the life context of people could inform the content, delivery and timing of health interventions to promote prompt and appropriate symptom recognition and help-seeking.

Our findings suggest that Ugandan women with vaginal symptoms often first attribute their symptoms to gynaecologic infections such as candidiasis and sexually transmitted infections. Only if symptoms persisted or did not respond to medications did women sometime attribute their symptoms to cancer. Similar symptoms for cancers and infectious diseases can offer great challenges to women especially if the illnesses have different pathways to care and treatment. In our earlier studies among women with an established diagnosis of cervical cancer in a hospital in the same region, most women attributed their vaginal symptoms of bleeding and discharge to cervical cancer and sexually transmitted infections. Most of these women sought care after long time periods and presented with advanced stage cancer.(4) In contrast, we found that women with breast symptoms who had heard of breast cancer mainly from radios and healthcare professionals during earlier visits to health facilities quickly attributed their symptoms to breast cancer and sought care accordingly. Similar findings were reported in an earlier Ugandan study about symptoms of cervical cancer.(12) This concurs with evidence from the UK, showing that awareness and knowledge of cancer symptoms are key in shaping people's attributions of symptoms and often prompt healthcare help-seeking.(39) We also found that pain, persistence of symptoms, or symptoms interfering with daily routines could trigger decisions to seek help. Mild and intermittent symptoms were either ignored or

For peer review only - http://bmjopen.bmj.com/site/about/guidelines.xhtml

For peer review only

interpreted as normal bodily changes, for example, due to the menopause or pregnancy, concurring with findings from a Malawi study of women with breast cancer.(13)

Women with potential breast and cervical cancer symptoms often engaged in lay consultations with their husbands, relatives and friends regarding their symptoms. These lay consultations were found to not only influence symptom attributions and illness inference, but also women's' help-seeking intentions and behaviour. Participants consulted with other people to obtain financial and practical support to seek help, as well as to gain insights into the causes and best effective remedies for their illnesses. Sanctioning of initial symptom interpretations by significant others has been shown in other studies set in the UK and Australia to influence help-seeking decisions among people with possible cancer symptoms or recently diagnosed with cancer.(40, 41) To avoid misleading advice when symptomatic women seek help from peers and significant others, the community as a whole need to be aware of cancer risk factors, symptoms and treatment. Therefore, public awareness campaigns to improve general awareness is important so that when women share their symptoms with other lay people, they may get appropriate help.

We found surprising negativity about traditional and complementary medicines, although some continued to consult traditional healers. This contrasts with earlier Ugandan qualitative findings from asymptomatic men and women, revealing that traditional and complementary medicines occupied a reasonable place in their lives mainly because the medicines were believed to work as they worked for their grandparents and ancestors, and could be useful to fill the void created by multiple barriers to biomedical care.(42) The participants in this study perceived the research assistants as healthcare providers; indeed, one was a nurse. Therefore,

For peer review only - http://bmjopen.bmj.com/site/about/guidelines.xhtml

it is possible that the reported low opinion towards use of traditional and complementary medicines could be due to social desirability bias.

Participants in this study reported several individual, interpersonal, sociocultural and health system structural barriers that delayed or prevented them from seeking healthcare. In public health facilities these barriers included lack of medicines, long waiting times and queues, and absenteeism among healthcare professionals. At a personal level, women commonly reported lack of money for transport and medical bills as their main barrier, especially in private healthcare facilities where patients pay directly for care. Most women also reported competing priorities and obligations that would hinder them from seeking healthcare. These obligations not only competed for their time but also for financial resources which were often diverted to school fees and other priorities instead of transport to health facilities and medical bills. Similar challenges to breast and cervical cancer healthcare help-seeking have been reported in other studies in SSA.(13, 14, 42) Insights into the perceived barriers to, and contextual factors that influence healthcare seeking from perspectives of the women can potentially inform targeted interventions to promote prompt healthcare seeking. Imposing health programs on women without clearly understanding their perspectives and contextual factors may not have the desired impact and would be an inefficient use of resources. Health planners and policy makers therefore need to make research findings to inform their decisions.

Limitations

Our study has some limitations. The study was preceded by a cross-sectional survey in which women were asked about potential breast and cervical cancer symptoms. It is possible that this could have influenced how women perceived and interpreted their symptoms.

For peer review only - http://bmjopen.bmj.com/site/about/guidelines.xhtml

Second, conducting interviews in women's homes may have influenced how some participants responded particularly in terms of the influence of family and social networks. However, we ensured that there were no non-participants at the interview venues.

Third, self-report of symptoms and help-seeking is subject to social desirability bias especially when participants may consider that their perceptions, beliefs and conduct could attract unfavourable comments from the research team or other people. However, as the research participants were assured of confidentiality, we contend that our findings accurately represent the perceptions and help-seeking behaviour of the symptomatic women included in this study.

Conclusion

Symptom attributions and consequent help-seeking may be influenced by information from radios or healthcare professionals, previous experiences will similar illnesses, approval or sanctioning by lay colleagues, and the onset, nature, persistence and severity of their symptoms as well as their response to medications. We found several interrelated sociocultural and structural barriers to healthcare help-seeking. In order to promote timely help-seeking and early detection of symptomatic breast and cervical cancer, targeted public health messages and campaigns to increase knowledge of women on breast and cervical cancer symptoms and services available need to be coupled with economic empowerment of women and improvement in access to health systems.

Acknowledgement

The authors wish to appreciate the research assistants and field coordinator for their thoroughness in conducting interviews in the midst of challenges including impassable roads and traversing difficult terrains on motorcycles. We are equally indebted to the research participants who freely discussed their ideas on the research questions, thereby providing us

For peer review only - http://bmjopen.bmj.com/site/about/guidelines.xhtml

with detailed information. We also thank the local leaders for their cooperation and participation in mobilizations and guidance for security and safety of research assistants during field visits.

Contributors Study concept and design: JM, FMW and ADM; acquisition of data: ADM and HW; analysis of data: ADM, JH and SS; interpretation of data: all authors; drafting of the manuscript: ADM; critical revision of the manuscript for important intellectual content: all authors; study supervision: JM, FMW, ADM and HW. Final approval of the submitted version of the manuscript and where to submit: all authors.

Funding Research reported in this article was jointly supported by the Cancer Association of South Africa (CANSA), the University of Cape Town and the South African Medical Research Council with funds received from the South African National Department of Health, GlaxoSmithKline (GSK) Africa Non-Communicable Disease Open Lab (via a supporting grant Project Number: 023), the United Kingdom Medical Research Council, MRC (via the Newton Fund). GSK provided in-kind scientific and statistical support as part of capacity strengthening. Award/Grant number is not applicable. The funders had no role in study design, data collection and analysis, preparation of the manuscript, decision to publish and where to publish. FMW is Director and SES is co-investigator of the multi-institutional CanTest Collaborative, which is funded by Cancer Research UK (C8640/A23385).

Competing Interests The authors declare that they have no competing interests.

Patient consent Not applicable.

For peer review only

Ethical approval This study protocol was approved by the Lacor Hospital Institutional Research Ethics Committee (**LHIREC 027/11/2016**). The protocol was thereafter registered and cleared by the Uganda National Council for Science and Technology (UNCST), registration number **HS60ES**. Additional permission to enter into the study communities was obtained from the local leaders at the district and sub-county levels. Written informed consents were obtained form study participants before interviews. Participants received a token of 25,000= (USD 6.8) for light snacks and soft drinks during interviews.

Disclaimers None.

Data sharing statement No additional data are available. Study dataset can be made available to third party following reasonable request to corresponding author.

For peer review only

References

1. Bray F, Ferlay J, Soerjomataram I, Siegel RL, Torre LA, Jemal A. Global cancer statistics 2018: GLOBOCAN estimates of incidence and mortality worldwide for 36 cancers in 185 countries. CA: a cancer journal for clinicians. 2018.
2. Mabula JB, Mchembe MD, Chalya PL, Giiti G, Chandika AB, Rambau PF, et al. Stage at diagnosis, clinicopathological and treatment patterns of breast cancer at Bugando Medical Centre in north-western Tanzania. Tanzania Journal of Health Research. 2012;14(4).
3. Pace LE, Mpunga T, Hategekimana V, Dusengimana J-MV, Habineza H, Bigirimana JB, et al. Delays in Breast Cancer Presentation and Diagnosis at Two Rural Cancer Referral Centers in Rwanda. The Oncologist. 2015;20(7):780-8.
4. Mwaka AD, Garimoi CO, Were EM, Roland M, Wabinga H, Lyratzopoulos G. Social, demographic and healthcare factors associated with stage at diagnosis of cervical cancer: cross-sectional study in a tertiary hospital in Northern Uganda. BMJ Open. 2016;6(1).
5. Dunyo P, Effah K, Udofia EA. Factors associated with late presentation of cervical cancer cases at a district hospital: a retrospective study. BMC Public Health. 2018;18(1):1156.
6. Joko-Fru WY, Miranda-Filho A, Soerjomataram I, Egue M, Akele-Akpo M-T, N'da G, et al. Breast cancer survival in sub-Saharan Africa by age, stage at diagnosis and human development index: A population-based registry study. Int Journal of Cancer. 2019.
7. Wabinga H, Ramanakumar AV, Banura C, Luwaga A, Nambooze S, Parkin DM. Survival of cervix cancer patients in Kampala, Uganda: 1995-1997. Brit Journal of Cancer. 2003;89(1):65-9.
8. Gondos A, Chokunonga E, Brenner H, Parkin DM, Sankila R, Borok MZ, et al. Cancer survival in a southern African urban population. Int Journal of Cancer. 2004;112(5):860-4.
9. Gondos A, Brenner H, Wabinga H, Parkin DM. Cancer survival in Kampala, Uganda. Brit Journal of Cancer. 2005;92(9):1808-12.
10. Sankaranarayanan R, Swaminathan R, Lucas E. Cancer survival in Africa, Asia, the Caribbean and Central America: International Agency for Research on Cancer Lyon; 2011.
11. van Schalkwyk SL, Maree JE, Wright SC. Cervical cancer: the route from signs and symptoms to treatment in South Africa. Reproductive health matters. 2008;16(32):9-17.
12. Mwaka AD, Okello ES, Wabinga H, Walter FM. Symptomatic presentation with cervical cancer in Uganda: a qualitative study assessing the pathways to diagnosis in a low-income country. BMC Women's Health. 2015;15(1):15.
13. Kohler RE, Gopal S, Miller AR, Lee CN, Reeve BB, Weiner BJ, et al. A framework for improving early detection of breast cancer in sub-Saharan Africa: A qualitative study of help-seeking behaviors among Malawian women. Patient education and counseling. 2017;100(1):167-73.
14. Mwaka AD, Wabinga HR, Mayanja-Kizza H. Mind the gaps: a qualitative study of perceptions of healthcare professionals on challenges and proposed remedies for cervical cancer help-seeking in post conflict northern Uganda. BMC Family Practice. 2013;14:193.
15. Gebremariam A, Addissie A, Worku A, Assefa M, Pace LE, Kantelhardt EJ, et al. Time intervals experienced between first symptom recognition and pathologic diagnosis of breast cancer in Addis Ababa, Ethiopia: a cross-sectional study. BMJ Open. 2019;9(11):e032228.
16. Ibrahim NA, Oludara MA. Socio-demographic factors and reasons associated with delay in breast cancer presentation: A study in Nigerian women. The Breast. 2012;21(3):416-8.

For peer review only - http://bmjopen.bmj.com/site/about/guidelines.xhtml

17. Brinton L, Figueroa J, Adjei E, Ansong D, Biritwum R, Edusei L, et al. Factors contributing to delays in diagnosis of breast cancers in Ghana, West Africa. Breast Cancer Res Tr. 2017;162(1):105-14.

18. Asoogo C, Duma SE. Factors contributing to late breast cancer presentation for health care amongst women in Kumasi, Ghana. Curationis. 2015;38:1-7.

19. Walter F, Webster A, Scott S, Emery J. The Andersen Model of Total Patient Delay: a systematic review of its application in cancer diagnosis. Journal of Health Services Research & Policy. 2012;17.

20. Scott SE, Walter FM, Webster A, Sutton S, Emery J. The model of pathways to treatment: conceptualization and integration with existing theory. Br J Health Psychol. 2013;18(1):45-65.

21. Niksic M, Rachet B, Duffy SW, Quaresma M, Møller H, Forbes LJL. Is cancer survival associated with cancer symptom awareness and barriers to seeking medical help in England? An ecological study. Brit Journal of Cancer. 2016;115(7):876-86.

22. Joffe M, Ayeni O, Norris SA, McCormack VA, Ruff P, Das I, et al. Barriers to early presentation of breast cancer among women in Soweto, South Africa. PloS One. 2018;13(2):e0192071.

23. Were EO, Buziba NG. Presentation and health care seeking behaviour of patients with cervical cancer seen at Moi Teaching and Referral Hospital, Eldoret, Kenya. East African medical journal. 2001;78(2):55-9.

24. Kazaura MR, Kombe D, Yuma S, Mtiro H, Mlawa G. Health seeking behavior among cancer patients attending Ocean Road Cancer Institute, Tanzania. East African journal of public health. 2007;4(1):19-22.

25. Birhanu Z, Abdissa A, Belachew T, Deribew A, Segni H, Tsu V, et al. Health seeking behavior for cervical cancer in Ethiopia: a qualitative study. International journal for equity in health. 2012;11:83.

26. Powe BD. Fatalism among elderly African Americans: Effects on colorectal cancer screening. Cancer nursing. 1995;18(5):385-92.

27. Beeken RJ, Simon AE, von Wagner C, Whitaker KL, Wardle J. Cancer fatalism: deterring early presentation and increasing social inequalities? Cancer Epidemiology Biomarkers & Prevention. 2011;20(10):2127-31.

28. Asoogo C, Duma SE. Factors contributing to late breast cancer presentation for health care amongst women in Kumasi, Ghana. Curationis. 2015;38(1):1287.

29. Drews CD, Greeland S. The Impact of Differential Recall on the Results of Case-Control Studies. International journal of epidemiology. 1990;19(4):1107-12.

30. Kopec JA, Esdaile JM. Bias in case-control studies. A review. Journal of epidemiology and community health. 1990;44(3):179-86.

31. MoFPED. POVERTY STATUS REPORT 2014. Structural change and poverty reduction in Uganda. Available at: https://wwwtralacorg/images/docs/6878/uganda-poverty-status-report-november-2014pdf. 2014;Accessed on 2nd June 2020.

32. MOH. Ministry of Health (MOH) [Uganda] and ORC Macro. 2006. Uganda HIV/AIDS Sero-behavioural Survey 2004-2005. Calverton, Maryland, USA: Ministry of Health and ORC Macro. Available at: https://dhsprogramcom/pubs/pdf/AIS2/AIS2pdf Accessed 2nd June 2020. 2006.

33. Patel S, Schechter MT, Sewankambo NK, Atim S, Kiwanuka N, Spittal PM. Lost in Transition: HIV Prevalence and Correlates of Infection among Young People Living in Post-Emergency Phase Transit Camps in Gulu District, Northern Uganda. PloS One. 2014;9(2):e89786.

34. Moodley J, Scott SE, Mwaka AD, Constant D. Development and validation of the African Women Awareness of CANcer (AWACAN) tool for breast and cervical cancer. 2019;14(8):e0220545.

For peer review only - http://bmjopen.bmj.com/site/about/guidelines.xhtml

35. Moodley J, Constant D, Mwaka AD, Scott SE, Walter FM. Mapping awareness of breast and cervical cancer risk factors, symptoms and lay beliefs in Uganda and South Africa. PLoS One. 2020;15(10):e0240788.

36. Francis JJ, Johnston M, Robertson C, Glidewell L, Entwistle V, Eccles MP, et al. What is an adequate sample size? Operationalising data saturation for theory-based interview studies. Psychology & health. 2010;25(10):1229-45.

37. Guest G, Bunce A, Johnson L. How Many Interviews Are Enough?: An Experiment with Data Saturation and Variability. Field Methods. 2006;18(1):59-82.

38. Bonsu AB, Ncama BP. Recognizing and appraising symptoms of breast cancer as a reason for delayed presentation in Ghanaian women: A qualitative study. PloS One. 2019;14(1):e0208773.

39. Simon A, Waller J, Robb K, Wardle J. Patient Delay in Presentation of Possible Cancer Symptoms: The Contribution of Knowledge and Attitudes in a Population Sample from the United Kingdom. Cancer Epidemiol Biomarkers Prev. 2010;19.

40. Macleod U, Mitchell ED, Burgess C, Macdonald S, Ramirez AJ. Risk factors for delayed presentation and referral of symptomatic cancer: evidence for common cancers. Br J Cancer. 2009;101 Suppl 2:S92-s101.

41. Emery JD, Walter FM, Gray V, Sinclair C, Howting D, Bulsara M, et al. Diagnosing cancer in the bush: a mixed-methods study of symptom appraisal and help-seeking behaviour in people with cancer from rural Western Australia. Family Practice. 2013.

42. Mwaka AD, Okello ES, Orach CG. Barriers to biomedical care and use of traditional medicines for treatment of cervical cancer: an exploratory qualitative study in northern Uganda. Eur Journal of Cancer Care. 2015;24(4):503-13.

For peer review only

For peer review only - http://bmjopen.bmj.com/site/about/guidelines.xhtml

In-depth Interview Guide

Understanding symptoms appraisal, help seeking and perceived barriers to healthcare seeking among women with potential symptoms of breast and cervical cancer in Uganda

Introduction

Thank you for accepting to participate in this study. The study is about understanding the way women with symptoms that could be due to breast or cervical cancer explain their symptoms and seek help for the them. In particular, we would like to understand the things that you consider when you are want to explain such symptoms as breast pain, swelling in the breast and discharge from the genitals o a woman. We also need to understand from you the factors that influence the way you seek for help.

To achieve our objective, we shall conduct an interview with you based on questions in our study guide. This interview will take about 45 minutes to one and half hours. Please keep in mind that there are no right or wrong answers to these questions. If I raise an issue or ask a question you don't want to talk about, just say so and we will move on to something else. Plase be free to ask any questions you will like to ask to help you undertand our interview. If you have any questions about the study, you can ask them below starting he interview or afterwards.

Note that you have been invited to participate in this interview because you have indicated that you have had some symptoms including pain and welling in the breasts, and discharge from your genitals during our earlier study. We are very grateful to have participate in this segment of the study.

Guiding questions

A: Detection and appraisal of bodily changes

1. **Thank you for agreeing to be interviewed today. I would like to start by asking you some questions around the changes/symptoms you mention related to your … (here relate to specific symptom mentioned e.g. breast lump, vaginal discharge).**
   Could you tell me more about this change?
   Probes:
   - What was the first thing that you noticed?
   - When did you notice the symptom? Use calendar prompt to assist, based on important landmarks e.g. Christmas, Easter, public holidays etc.
   - Has the symptom changed since you first noticed it? If yes, how has this changed?

2. **Could you describe how you felt when you noticed this symptom (refer to change/symptom e.g. finding a breast lump, vaginal bleeding)?**
   Probe:
   - Emotional response to symptom? E.g. Afraid, anxious, embarrassed, not concerned, denial, concerned that it was serious
   - Why did you feel that way?

For peer review only - http://bmjopen.bmj.com/site/about/guidelines.xhtml

3. **When you first noticed the symptom(s), what did you think was causing the symptom? What made you think that?**
   Probe:
   - Did you think it meant you had any particular illness? If so what illness and what made you think this way?
   - At any point did you think it is/was an infection? Why did you think this?
   - Did you think it was something minor/nothing to worry about? If so, what made you think this?
   - Were you unsure about what it meant? If so what made you unsure?

4. **Since then, have you had any other thoughts about what it might be? (if changed thoughts of what the symptoms could be caused by then, probe reasons for change. E.g. what made you think that?)**

5. **Have you had a symptom like this before? If yes Probe when? What did you do? What was the outcome?**

6. **Do you know of anyone who had a similar symptom? [say the symptom]**

   If Yes, what was the cause of their symptom? What did they do about it? What was the outcome?

7. **Have you ever heard about this symptom/change anywhere? If yes where?**
   Probe: at the clinic, discussed with my family/friends, pamphlets, social media/ posters taxis or trains/ radio/television? What information did each of these sources provide about such a symptom?

8. **Did you discuss this current change/symptom with anyone?**
   Probe:
   If Yes
   - Whom?
   - What did this person/s say? Was it helpful?
   - Did it influence your views on the change/symptom? If Yes how?
   - Why did you discuss with that person (name/relation)?

   If No
   - Why did you not discuss with anyone? Probes feelings of anxiety, not worried about symptom etc.

9. **How do you think people in your community view someone with this symptom (refer to symptoms participant named)**
   Probe:
   - Is there any stigma attached to having this symptom?
   - Are people supportive/empathetic with people with these kinds of symptoms in your community?

For peer review only - http://bmjopen.bmj.com/site/about/guidelines.xhtml

- Is this symptom generally discussed by family and friends?

10. Has this symptom affected your life in anyway?

Probe:
If yes, how has it affected your life?

11. Have you tried to manage the symptoms yourself? Did you try anything to help relieve / resolve the symptoms?

B: Help-seeking behaviour and estimated dates of symptoms

12. Have you gone to have this change/symptom (name symptom) checked by anyone?
If YES
Probes:
- Who did you visit?
- Why did you choose to go there? Could you tell me **what** made you decide to seek care? Was there anything in particular that made you decide to seek assistance?
- When did you go to have it checked? *Use calendar prompt*
- Can you tell me about your experience seeking advice or assistance from …

Have you had the symptom checked by anyone else? (Repeat prompts above)

- Have you visited a traditional healer? If visited traditional healer, was it after or before visiting the health facilities and why before or after (rationale)? What did the traditional healer say about the symptom? *Note participant might discuss this in earlier response to question 12.*

- Have you visited a health care facility or health care provider? If yes when did you visit health care provider? Probe only if person waited to see health care provider - Is there anything that made you wait before getting checked by a health care provider?

Note participant might discuss this in earlier response to question 12.

If NO (woman has not gone to seek care)
- Have you thought about getting checked?
- Do you intend to seek care? If so, from whom? When?
- Are there any reasons for not seeking care from a healthcare provider?
- Is there anything that has stopped you from seeking care?
  e.g. lack of pain, past history of this symptom, belief that it might go away, think/thought it is/was minor, partner, family or friends
- Access - Are there any problems getting to the clinic?
  - Is the cost of getting to a clinic or health care provider a problem?

For peer review only - http://bmjopen.bmj.com/site/about/guidelines.xhtml

For peer review only

- If person working is it a problem to take time off work?
- If person looking after children is it a problem to get to the clinic because you are looking after children

Is there anything that would make you get checked by a healthcare provider?

We are now coming to the end of our discussion. Can I take a minute to pause to see if there are any questions I might have missed?

13. Do you have any other comments, or questions that you would like to ask?

For peer review only

Thank you for participating in this study. Your views are very important to us.

For peer review only - http://bmjopen.bmj.com/site/about/guidelines.xhtml

Checklist: **Symptom appraisal, help seeking and perceived barriers to healthcare seeking in Uganda: An exploratory study among women with potential symptoms of breast and cervical cancer**

		Reporting Item	Page Number
**Title**			
	#1	Concise description of the nature and topic of the study identifying the study as qualitative or indicating the approach (e.g. ethnography, grounded theory) or data collection methods (e.g. interview, focus group) is recommended	1
**1Abstract**			
	#2	Summary of the key elements of the study using the abstract format of the intended publication; typically includes background, purpose, methods, results and conclusions	2
**Introduction**			
Problem formulation	#3	Description and significance of the problem / phenomenon studied: review of relevant theory and empirical work; problem statement	4 - 5
Purpose or research question	#4	Purpose of the study and specific objectives or questions	6
**Methods**			
Qualitative approach and research paradigm	#5	Qualitative approach (e.g. ethnography, grounded theory, case study, phenomenology, narrative research) and guiding theory if appropriate; identifying the research paradigm (e.g. postpositivist, constructivist / interpretivist) is also recommended; rationale. The rationale should briefly discuss the justification for choosing that theory, approach, method or technique rather than other options available; the assumptions and limitations implicit in those choices and how those choices influence study conclusions and transferability. As appropriate the rationale for several items might be discussed together.	6

For peer review only

Researcher characteristics and reflexivity	#6	Researchers' characteristics that may influence the research, including personal attributes, qualifications / experience, relationship with participants, assumptions and / or presuppositions; potential or actual interaction between researchers' characteristics and the research questions, approach, methods, results and / or transferability	N/A
Context	#7	Setting / site and salient contextual factors; rationale	6
Sampling strategy	#8	How and why research participants, documents, or events were selected; criteria for deciding when no further sampling was necessary (e.g. sampling saturation); rationale	7
Ethical issues pertaining to human subjects	#9	Documentation of approval by an appropriate ethics review board and participant consent, or explanation for lack thereof; other confidentiality and data security issues	35
Data collection methods	#10	Types of data collected; details of data collection procedures including (as appropriate) start and stop dates of data collection and analysis, iterative process, triangulation of sources / methods, and modification of procedures in response to evolving study findings; rationale	7
Data collection instruments and technologies	#11	Description of instruments (e.g. interview guides, questionnaires) and devices (e.g. audio recorders) used for data collection; if / how the instruments(s) changed over the course of the study	7
Units of study	#12	Number and relevant characteristics of participants, documents, or events included in the study; level of participation (could be reported in results)	7
Data processing	#13	Methods for processing data prior to and during analysis, including transcription, data entry, data management and security, verification of data integrity, data coding, and anonymisation / deidentification of excerpts	8
Data analysis	#14	Process by which inferences, themes, etc. were identified and developed, including the	8

For peer review only

		researchers involved in data analysis; usually references a specific paradigm or approach; rationale	
Techniques to enhance trustworthiness	#15	Techniques to enhance trustworthiness and credibility of data analysis (e.g. member checking, audit trail, triangulation); rationale	8
**Results/findings**			
Syntheses and interpretation	#16	Main findings (e.g. interpretations, inferences, and themes); might include development of a theory or model, or integration with prior research or theory	9
Links to empirical data	#17	Evidence (e.g. quotes, field notes, text excerpts, photographs) to substantiate analytic findings	9 - 28
**Discussion**			
Integration with prior work, implications, transferability and contribution(s) to the field	#18	Short summary of main findings; explanation of how findings and conclusions connect to, support, elaborate on, or challenge conclusions of earlier scholarship; discussion of scope of application / generalizability; identification of unique contributions(s) to scholarship in a discipline or field	29 - 32
Limitations	#19	Trustworthiness and limitations of findings	32 - 33
**Other**			
Conflicts of interest	#20	Potential sources of influence of perceived influence on study conduct and conclusions; how these were managed	34
Funding	#21	Sources of funding and other support; role of funders in data collection, interpretation and reporting	34

For peer review only

BMJ Open

Symptom appraisal, help seeking and perceived barriers to healthcare seeking in Uganda: An exploratory study among women with potential symptoms of breast and cervical cancer

Journal:	*BMJ Open*
Manuscript ID	bmjopen-2020-041365.R2
Article Type:	Original research
Date Submitted by the Author:	30-Dec-2020

Complete List of Authors:	Mwaka, Amos; Makerere University College of Health Sciences, Medicine<br>Walter, Fiona; Cambridge University, Dept of Public Health and Primary Care; University of Melbourne VCCC<br>Scott, Suzanne; King's College London, Unit of Social & Behavioural Sciences<br>Harries, Jane; University of Cape Town Faculty of Health Sciences, Women's Health Research Unit<br>Wabinga, Henry; Makerere University College of Health Sciences, Medicine<br>Moodley, Jennifer; University of Cape Town Faculty of Health Sciences, Faculty of Health Sciences

**Primary Subject Heading**:	Global health
Secondary Subject Heading:	Oncology
Keywords:	Breast tumours < ONCOLOGY, Gynaecological oncology < ONCOLOGY, PRIMARY CARE

SCHOLARONE™
Manuscripts

I, the Submitting Author has the right to grant and does grant on behalf of all authors of the Work (as defined in the below author licence), an exclusive licence and/or a non-exclusive licence for contributions from authors who are: i) UK Crown employees; ii) where BMJ has agreed a CC-BY licence shall apply, and/or iii) in accordance with the terms applicable for US Federal Government officers or employees acting as part of their official duties; on a worldwide, perpetual, irrevocable, royalty-free basis to BMJ Publishing Group Ltd ("BMJ") its licensees and where the relevant Journal is co-owned by BMJ to the co-owners of the Journal, to publish the Work in this journal and any other BMJ products and to exploit all rights, as set out in our licence.

The Submitting Author accepts and understands that any supply made under these terms is made by BMJ to the Submitting Author unless you are acting as an employee on behalf of your employer or a postgraduate student of an affiliated institution which is paying any applicable article publishing charge ("APC") for Open Access articles. Where the Submitting Author wishes to make the Work available on an Open Access basis (and intends to pay the relevant APC), the terms of reuse of such Open Access shall be governed by a Creative Commons licence – details of these licences and which Creative Commons licence will apply to this Work are set out in our licence referred to above.

Other than as permitted in any relevant BMJ Author's Self Archiving Policies, I confirm this Work has not been accepted for publication elsewhere, is not being considered for publication elsewhere and does not duplicate material already published. I confirm all authors consent to publication of this Work and authorise the granting of this licence.

For peer review only

Symptom appraisal, help seeking and perceived barriers to healthcare seeking in Uganda: An exploratory study among women with potential symptoms of breast and cervical cancer

Amos Deogratius Mwaka[1], Fiona M. Walter[2]*, Suzanne E. Scott[3], Jane Harries[4], Henry Wabinga[5], Jennifer Moodley[4, 6, 7]

Authors' affiliations

1. Department of Medicine, School of Medicine, College of Health Sciences, Makerere University. P.O Box 7072, Kampala, Uganda.
2. The Primary Care Unit, Department of Public Health & Primary Care, University of Cambridge, UK.
3. Faculty of Dentistry, Oral & Craniofacial Sciences, King's College London, UK.
4. Women's Health Research Unit, School of Public Health and Family Medicine, Faculty of Health Sciences, University of Cape Town; Anzio Road. Observatory, 7925, Cape, Town, South Africa.
5. Department of Pathology, School of Biomedical Sciences, College of Health Sciences, Makerere University. P.O Box 7072, Kampala, Uganda.
6. Cancer Research Initiative, Faculty of Health Sciences, University of Cape Town; Anzio Road, Observatory, 7925, Cape, Town, South Africa.
7. SAMRC Gynaecology Cancer Research Centre, Faculty of Health Sciences, University of Cape Town, Anzio Road. Observatory, 7925, Cape Town, South Africa. ORCID 0000-0002-9398-5202

Emails:

Amos Deogratius Mwaka: mgratius@gmail.com

Fiona M. Walter: fmw22@medschl.cam.ac.uk

Suzanne Scott: suzanne.scott@kcl.ac.uk

Jane Harries: jane.harries@uct.ac.za

Henry Wabinga: hwabinga@gmail.com

Jennifer Moodley: jennifer.moodley@uct.ac.za

*Corresponding author: Fiona M. Walter. The Primary Care Unit, Department of Public Health & Primary Care, University of Cambridge, UK. Email: fmw22@medschl.cam.ac.uk

Word count: 7, 598.

For peer review only - http://bmjopen.bmj.com/site/about/guidelines.xhtml

Abstract

Objective: We assessed the process of recognising abnormal bodily changes, interpretations and attributions, and help-seeking behaviour among community-based Ugandan women with possible symptoms of breast and cervical cancer, in order to inform health interventions aiming to promote timely detection and diagnosis of cancer.

Design: Qualitative in-depth interviews.

Setting: Rural and urban communities in Uganda

Participants: Women who participated in the African Women Awareness of CANcer (AWACAN) cross-sectional survey who disclosed potential breast and cervical cancer symptoms were eligible; recruitment was purposive. Interviews were conducted in women's homes, lasted between 40 and 90 minutes, were audio-recorded, transcribed verbatim, and translated to English. Thematic analysis was used to identify themes and subthemes, underpinned by the conceptual framework of the Model of Pathways to Treatment.

Results: 23 women were interviewed: 10 had potential symptoms of breast cancer and 13 of cervical cancer. Themes regarding symptom appraisal and help-seeking included the; (i) detection and interpretation of abnormal bodily sensations; (ii) lay consultations regarding bodily changes; (iii) iterative process of inferring and attributing illnesses to the bodily changes; (iv) restricted disclosure of symptoms to lay people due to concerns about privacy and fear of stigmatization; (v) help-seeking from multiple sources including both traditional and biomedical health practitioners, and (vi) multiple perceived barriers to help-seeking including long waiting times, lack of medicines, absenteeism of healthcare professionals, and lack of money for transport and medical bills.

Conclusion: Women with potential symptoms of breast and cervical cancer undergo complex processes of symptom interpretation, attributing symptoms or inferring illness, and lay consultations before undertaking help-seeking and management. Increasing community

For peer review only - http://bmjopen.bmj.com/site/about/guidelines.xhtml

understanding of breast and cervical cancer symptoms, and tackling perceived barriers to health-seeking, could lead to prompt and appropriate symptom appraisal and help-seeking, and contribute to improving cancer outcomes.

Key words: Breast cancer, cervical cancer, Symptoms appraisal and attributions, help-seeking

Strengths and limitations of this study

- To our knowledge, this is the first study set in Uganda to explore the process of recognition of abnormal bodily changes, interpretations and attributions, and help-seeking behaviour among community-based women with possible symptoms of breast or cervical cancer.
- The qualitative nature of the inquiry and strong theoretical underpinning with the Model of Pathways to Treatment enabled in-depth exploration of the research question.
- Conducting interviews by female research assistants in the home of the participants allowed for freedom of expression and in-depth responses by the majority of participants.
- Self-report of symptoms and help-seeking is subject to social desirability bias especially when participants consider that their actual perceptions, beliefs and conducts could attract unfavourable comments from the research team or other people.

For peer review only - http://bmjopen.bmj.com/site/about/guidelines.xhtml

Background

In low- and middle-income countries (LMICs), including most sub Saharan African (SSA) countries, breast and cervical cancers are the most common cancers among women. The incidence of cervical cancer in Eastern and Southern Africa are 40.1 and 43.1 per 100,000 women respectively, while that of breast cancer are 29.9 and 46.2 per 100,000 women respectively.(1) In these regions, patients with breast and cervical cancer are often diagnosed in advanced stages.(2-4) In Uganda and Ghana, 66% of cervical cancer patients were diagnosed in advanced stages.(4, 5) In Rwanda, 76% of breast cancer patients were diagnosed in advanced stages.(3) A recent registry-based study in SSA showed that more than half (64.9%) of patients with breast cancer are diagnosed in advanced stages and experience low survival.(6) Advanced stage cancer at diagnosis is associated with poorer survival.(7-10) In SSA, breast cancer overall relative survival at one year and five years were 86.1% (84.4–87.6), and 59.0% (56.3–61.6) respectively. Relative survival varied between registries but ranged from 21.6% (8.2 – 39.8) at three years in Malawi to 84.5% (70.6 – 93.5) in Namibia.(6)

Poor recognition of cancer symptoms by both patients and primary healthcare professionals may contribute to the advanced stage at diagnosis and poor survival of breast and cervical cancer in SSA.(11-14) Presentation at the healthcare facilities and eventual diagnosis of cancer is often long. In Ethiopia and Nigeria, 36% and 81.6% of patients with breast cancer spent more than 90 days with symptoms before seeking care with a healthcare professionals.(15, 16) In Ghana, use of traditional and complementary medicines were associated with prolonged time to help seeking.(17, 18) Several patient factors including individual, socio-cultural and health system factors interact to influence the patients' pathway to treatment for their cancer symptoms.(19, 20) In general, patients' awareness of cancer symptoms has been shown to be associated with cancer survival.(21) Similarly, in South Africa, breast cancer patients with

For peer review only - http://bmjopen.bmj.com/site/about/guidelines.xhtml

higher education attainment and with greater awareness and knowledge of breast cancer symptoms were statistically significantly more likely to be diagnosed with early stage disease.(22) In addition, self-management, use of traditional medicines and perceived multiple barriers to medical care have been associated with longer time to healthcare seeking for breast and cervical cancer symptoms in SSA.(3, 23-25) In the USA, cancer fatalism among African Americans and Hispanics was associated with advanced stage at diagnosis, inadequate uptake of treatment, and less prompt healthcare seeking for cancer symptoms.(26, 27) However, most of these studies have involved hypothetical help-seeking or retrospective accounts of those already diagnosed with cancer.(12, 13, 28) Hypothetical help-seeking reported by people without real symptoms may not be similar to real help-seeking decisions because of somatic and emotional experiences. On the other hand retrospective recall of symptoms and decision making processes could be enhanced or minimized because of the cancer diagnosis.(29, 30) In addition, help-seeking accounts of patients already diagnosed with cancers are likely to be influenced by their cancer diagnoses and multiple interactions with healthcare professionals. This may not be the case in symptomatic women without a cancer diagnosis and in the community.

Understanding factors influencing appraisal and help-seeking decisions for women with possible symptoms of breast and cervical cancer is a critical step to underpin the development of interventions to promote prompt recognition of symptoms and healthcare help-seeking. In this study set in Uganda, we sought to understand the processes of symptom recognition, appraisal and attribution, and perceived barriers to healthcare seeking among community women with recent experiences of possible symptoms of breast and cervical cancer, regardless of whether they had sought help or been diagnosed with cancer, in order to inform the design

For peer review only

For peer review only - http://bmjopen.bmj.com/site/about/guidelines.xhtml

of interventions to promote prompt symptom appraisal and healthcare help-seeking for women in SSA.

Methods

Design

A qualitative in-depth interview study.

Study setting

The study was conducted across two sites in northern Uganda: an urban site in Gulu district and a rural site in Nwoya district. Most people in northern Uganda earn their livelihood from subsistence agricultural practices; there are low levels of education and chronic intergenerational poverty in the region. Although the level of poverty has decreased since the return of peace in 2006, the region still remains the poorest in Uganda. Greater proportions of households in the region are larger in size and less likely to have two meals a day than in the rest of Uganda.(31) HIV infection has consistently remained higher in this region compared to other parts of Uganda.(32, 33)

Study population and recruitment

This interview study was part of a larger study involving a cross-sectional survey using the AWACAN tool.(34, 35) Eligibility criteria for this study included: women aged 18 years and over, self-reported experience of symptoms suggestive of possible breast or cervical cancer within the past three months, who had participated in the survey, spoke English or the Luo/Acholi language, and were willing to provide written consent to participate in an interview. Participants also needed to report two or more possible symptoms of breast or cervical cancer, but not have had a breast or cervical cancer diagnoses.

For peer review only

For peer review only - http://bmjopen.bmj.com/site/about/guidelines.xhtml

In the urban site, 41 potentially eligible women were identified via the survey, but 15 did not meet inclusion criteria. 14 women were purposively selected to include a range of symptoms and age; these and the remaining 12 women were advised to seek help for their symptoms. In the rural site, 25 AWACAN survey participants reported symptoms suggestive of breast or cervical cancer, but 11 did not meet inclusion criteria. All the remaining 14 were considered for interviews. Interviews were conducted during October 2018 in the urban site and January - February 2019 in the rural site; they continued until data saturation was reached in both localities.(36, 37)

Data collection

Two female research assistants conducted in-depth interviews in both sites. One research assistant led the interviewing process while the other operated the digital audio-recorder and took field notes, especially of non-verbal communication and events during interviews. It was necessary for the research assistants to work in pairs to ensure their personal security as they travelled in the villages. Data collection was mainly conducted in the women's homes, usually in quiet rooms without others present, and occasionally under trees in the family compounds. The research assistants used piloted interview guides, underpinned by the concepts of the Model of Pathways to Treatment.(19, 20) The interview guide included how bodily changes were first recognised, what happened immediately after detection of a symptom, who were consulted about the symptoms, and the steps undertaken to seek help (see Supplementary File 1). Socio demographic data including age, marital status, number of biological children, highest educational attainment, and HIV status were also collected. Each interview lasted between 45 and 90 minutes, and all interviews were audio-recorded.

Data analysis

For peer review only - http://bmjopen.bmj.com/site/about/guidelines.xhtml

Audio-recordings were transcribed verbatim by the research assistants. ADM read transcripts and conducted preliminary analyses during data collection in order to determine data saturation. Before full analyses, ADM reviewed ten transcripts (five from each site) against the recordings to ensure appropriate details were captured and transcripts labelled appropriately. ADM read three transcripts in detail, developed codes, and shared the codebook with the wider study team. Codes were redefined and refined as necessary. Atlas.ti version 6.1 was used to support data analysis. Coded data segments from all transcripts were aggregated and retrieved for detailed analyses. These underpinned the inductive development of themes and subthemes, focussing on the research objective and the concepts in the Model of Pathways to Treatment.

Patient and Public Involvement

Patients were not involved in the planning and implementation of this study. However, the public was involved through the district and community leaders. The district health officers and district women representatives on the district councils of the two study districts were involved in the study. These local leaders were part of our Project Advisory Committee (PAC) which we formed to provide advice and guidance to the study team to ensure that the study is conducted in a culturally acceptable manner, and the findings are relevant to the local context in order to inform local and national health policies on targeted interventions to promote cancer awareness and early detection. The study objectives and tools were discussed with these stakeholders. They provided guidance on how our study could refined to best fit the local context. We therefore refined the study tools to capture aspects of women's lives that relate to information seeking and access, healthcare seeking, and decision making in the families. In addition, the local leaders will be involved in disseminations of study findings. Their presence during dissemination is expected to make the population more confident in the results. The

For peer review only - http://bmjopen.bmj.com/site/about/guidelines.xhtml

involvement of the local decision makers potentially increases the chance that this study findings could be acceptable and put into use.

Results

Characteristics of the participants

23 women were interviewed: 10 had possible symptoms of breast cancer and 13 had possible symptoms of cervical cancer. Two self-reported being HIV positive and on antiretroviral therapies. Overall, the median age was 31 years (range: 18 – 66 years; urban site 27 years (range: 22 – 66 years), rural site 41 years (range: 18 – 64 years)), (see **Table 1).**

For peer review only

Table 1: Characteristics of respondents and reported symptoms

	Study site		Age group (Years)	Marital status	Education attainment	Occupation	Symptoms	HIV status
**Participants**	Urban	Rural						
P1	X		31 - 50	Widow	Primary 5	Unemployed	Pain and lump in breast	Negative
P2	X		31 - 50	Married	Primary 4	Business/trader	Pain and lumps in both breasts	Positive
P3	X		51 - 70	Widow	Primary 6	Business/trader	Pain and lump in breast	Negative
P4	X		18 - 30	Separated	Primary 3	Waitress in restaurant	SVD*, Vaginal bleeding between periods, Persistent LAP*	Negative
P5	X		18 - 30	Married	Senior 4	Business/trader	Pain and lump in breast	Negative
P6	X		18 - 30	Married	Senior 4	Business/trader	Vaginal bleeding during/after sex, Persistent LAP	Negative
P7	X		18 - 30	Married	Tertiary education	Business/trader	Pain and lump in breast	Negative
P8	X		18 - 30	Married	Senior 3	Business/trader	SVD, Persistent LAP, Pain and discomfort during sex	Negative
P9	X		31 - 50	Widow	Primary 3	Business/trader	SVD, Persistent LAP	Negative
P10	X		18 - 30	Married	Primary 7	Unemployed	Lumps in armpits and both breasts	Negative
P11	X		51 - 70	Married	Primary 6	Unemployed	Pain and lumps in both breasts	Unknown
P12		X	51 - 70	Married	No formal education	Unemployed	Lumps in armpits and both breasts	Negative
P13		X	31 - 50	Married	Primary 2	Unemployed	SVD, Persistent LAP, Pain and discomfort during sex	Positive
P14		X	18 - 30	Married	Primary 5	Unemployed	Pain in nipple, lump in breast	Negative
P15		X	31 - 50	Married	Primary 2	Unemployed	Lump and pain in breast, dimpling of breast skin like orange peel	Negative
P16	X		18 - 30	Separated	Primary 6	Unemployed	SVD, Persistent LAP	Negative
P17		X	31 - 50	Married	Primary 6	Business/trader	Persistent LAP, discomfort during sex	Negative
P18		X	31 - 50	Married	No formal education	Unemployed	Persistent LAP, discomfort during sex	Negative

For peer review only

P19		X	51 - 70	Married	Primary 1	Unemployed	SVD, Persistent LAP, Pain and discomfort during sex	Negative
P20		X	18 - 30	Married	Primary 3	Unemployed	SVD, Vaginal bleeding between periods, Persistent LAP	Negative
P21		X	31 - 50	Married	Primary 3	Unemployed	SVD, Vaginal bleeding between periods, Persistent LAP	Negative
P22		X	18 - 30	Married	Primary 7	Unemployed	Persistent LAP, discomfort during sex	Negative
P23	X		18 - 30	Married	Tertiary education	Accountant	SVD, Persistent LAP, Itching in the vagina	Negative

*LAP = Lower abdominal pain, SVD = Smelly vaginal discharge

For peer review only

Main themes

Themes regarding symptom appraisal and help-seeking intervals included the; (i) detection and interpretations of abnormal bodily sensations; (ii) lay consultations regarding bodily changes; (iii) iterative process of inferring and attributing illnesses to the bodily changes; (iv) nondisclosure of symptoms to lay people due to concerns about privacy and fear of stigmatization; (v) help-seeking from multiple sources including both traditional and biomedical health practitioners, and (vi) multiple perceived barriers to biomedical care. Supporting quotations are presented followed by participant characteristics (age group and symptoms of cancer type).

(i) Detection and interpretation of abnormal bodily sensations

Detection and initial interpretation of bodily changes as normal or abnormal punctuated participants' thoughts and course of actions regarding their symptoms. The circumstances when bodily changes were first realised were critical to initial interpretations.

Expected bodily changes within a person's lifespan

The age of the woman was critical in deciding whether a certain bodily change could be due to an illness or normal changes. Young women could readily associate bodily changes with menstrual cycles and possibilities of pregnancies, while older women were more concerned with menopausal changes and age-related ailments.

> "That problem, I thought it was my state of menopause that I reached and I stopped giving birth. I thought that way because I wasn't conceiving or giving birth anymore", (**Rural_51-70_Cervical**).

Participants who had ever breastfed or been pregnant initially perceived their breast symptoms to be due to usual bodily changes, and paid little attention until symptoms persisted or became painful

> "When this lump came up, I thought that probably it is as usual when it would happen when I'm breast feeding. But now I'm not even breast feeding, but the lump on my right armpit is still there and painful", (**Rural_51-70_Breast**).

Chronology with recent events

Bodily changes were interpreted based on circumstances around their onset. For example, a participant who started to experience abnormal bodily changes following a medical procedure or surgery would infer the changes to the procedure.

> "I thought that was an effect from the contraceptive I had inserted in my arm. Before I started using that implant, those symptoms were not there; it was not happening", (**Urban_18-30_Breast**).

Some participants interpreted their bodily changes in respect to their medical histories including post-abortion complications and effect of birth trauma following deliveries.

> "I don't really know [. . .]. I also had a miscarriage with him but my uterus wasn't "cleaned". The doctor said after such an incident one has to undergo "cleaning" of the uterus in order to be healthy once again. So that is why I feel it was that problem causing

For peer review only - http://bmjopen.bmj.com/site/about/guidelines.xhtml

> the symptoms [. . .]. I feel that probably there are some remaining dirt in my womb", (**Rural_31-50_Cervical**).

Poor personal hygiene, crowded living environment and risk of infections

Living in crowded unhygienic dwellings especially rented units influenced the initial interpretations of bodily changes experienced. Women considered personal hygiene as critical determinants of gynaecologic symptoms. Women who thought their personal hygiene was compromised were quick to think of hygiene-related causes or infections, especially if they did not have a sexual partner who they could suspect for transmitting a sexually transmitted disease. This view was predominantly held among younger urban women with gynaecologic symptoms.

> "I first thought it could be candida . . . because it also causes itching in the vagina. I thought of the fact that there are many tenants here, so the issue of sharing the bathroom could be the problem; you could easily get it", (**Urban_18-30_Cervical**).

> "May be poor hygiene, because from school you bathe in a crowded place. You bathe where everybody shares things; you share the buckets. So I think poor hygiene was one of the factors, yeah", (**Urban_18-30_Cervical**).

(ii) Lay consultations regarding bodily changes

Several factors and concerns influenced women to share their symptoms with other people, including husbands, siblings, trusted friends and sometimes parents. Participants engaged in lay consultations for various reasons.

For peer review only - http://bmjopen.bmj.com/site/about/guidelines.xhtml

Seeking financial and practical support

Most participants shared their symptoms with significant others to solicit financial and practical support. These women needed to reach the healthcare facilities for care, but lacked money or means of transport.

> "I thought that being my husband, if I told him he would take me to the health facility. He told me that he would have taken me . . . but he doesn't have the money. I didn't share it with anyone else because nobody else could help me with the money", (**Rural_31-50_Cervical**).

Learn and benefit from experiences of others

Many participants shared their symptoms with other people either because they trusted them or wanted to know more from them to help them decide the course of actions for their symptoms. Some of the confidants were healthcare professionals related to the participants or had had similar symptoms in the past and or were considered to know more about the symptoms.

> "I tried to tell my Mother in-law, and also my sister in-law who had recently returned home, then later I told my husband. I shared with her because I felt she was a mature person and could be having some ideas about it. So she just responded by saying; that definitely needed me to go to the hospital and was not to be tampered with [. . .]. I chose her because I felt she was close to me and we were relating so well", (**Rural_18-30_Breast**).

For peer review only

"They looked to be more informed that is why I also went to them. One of them told me that she also experienced those symptoms when she was pregnant but now it's not there. That she went to the health facility and was treated, so she was saying that if I also go and get treatment I will feel better", (**Urban_18-30_Cervical**).

Social accountability and avoidance of suspicion from family

A few participants shared with family, especially when the woman found it difficult to accomplish her roles in the family because of her illness.

"The reason as to why I decided to tell them is because when I have the pain sometimes I need to lie down for a while to rest and calm the pain. But if you don't tell them (in-laws) they may think you are just a lazy woman who doesn't want to do work, so I had to tell them so that they can be aware", (**Urban_18-30_Breast**).

A few participants shared their gynaecologic symptoms with their husbands not just to get financial support to seek care, but rather because they wanted to avoid their husbands suspecting them of engaging in sexual intercourse with other men.

"I decided to share with him (husband) because I didn't want him to suspect me of having cheated on him and got the infection. So I told him that I had never cheated on him or had these kind of symptoms before; so it was him who had infected me with it", (**Rural_18-30_Cervical**).

(iii) Nondisclosure of symptoms to lay people

For peer review only - http://bmjopen.bmj.com/site/about/guidelines.xhtml

While most participants shared their symptoms, most were keen not to share with people they did not trust.

Privacy concerns

Majority of participants considered symptoms of breast and cervical symptoms as private and not appropriate to share with just anyone.

> "By the way sharing your personal issues anyhow with anyone makes you become a fool. For me, once you are mature enough, don't go out sharing your issues anyhow", (**Rural_31-50_Cervical**).

Stigma and gossiping

Some participants who did not share their symptoms were concerned about gossiping and becoming objects of public discussions.

> "Some people like rumour mongering. For example, they can say such a person said this, they could now be having HIV/AIDS. They can talk; that you know, this person said they are in this state and that is already rumour mongering done. So they will be circulating your name. It is because I fear lies of people [. . .]. That is why it is very hard to share with people any secrets, because once you do, news go all over. This is why I no longer like sharing anyhow", (**Rural_51-70_Cervical**).

Self-perceived stigma could be an issue among symptomatic women, especially those with cervical symptoms.

For peer review only - http://bmjopen.bmj.com/site/about/guidelines.xhtml

> "As for other people, if you start to share your issues with them, they may judge you without assessing; they will end up airing your issues to people with other funny added lies", (**Rural_31-50_Cervical**).

Low expectations from lay consultations

Some participants had low expectations from lay consultations and therefore preferred formal healthcare seeking as the only and best response to illness symptoms.

> "I felt I should go straight to the hospital so that the doctor himself examines me; to me sharing with someone else here wouldn't yield much because they may mention something that will never be real compared with what the doctors will examine and find out", (**Urban_31-50_Cervical**).

(iv) Symptoms attributions: inferring illness

Participants attributed their symptoms to various disorders including infections and cancers. Symptom attributions were guided by the nature of onset, evolution and persistence of symptoms, participants' own previous experiences, family history of illnesses, response to attempted treatments, sanctioning/disapprovals from lay consultations, and messages from the radio or healthcare professionals.

Symptoms could be due to cancer

Gynaecologic symptoms that were unfamiliar and which persisted were often attributed to cervical cancer. These women expressed a lot of worries as they were inferring illness, mostly related to death and orphanhood.

For peer review only - http://bmjopen.bmj.com/site/about/guidelines.xhtml

"I have been asking myself what this could be! It kept me worried a lot. It was worrying me; I used to say that the symptoms I'm experiencing could be cancer and if it is, then that would be too bad. That is what was moving in my mind [. . .]. I just felt that cancer disease is one that can definitely lead to things such as that. I thought so because cancer is a very bad disease, it is really a bad disease . . . it hurries you and you will be short lived compared to other diseases", (**Urban_**31-50**_Cervical**).

Participants who ever screened for cervical cancer considered cancer as possible explanation for their symptoms but were unsure as they had tested negative on earlier screening tests.

"I have been worried about these symptoms and signs. I first thought it was cancer, but I went for cancer screening two years ago, so am still waiting for one more year to go and test again. I thought it could be cancer because cancer is the most dangerous infection, so people fear it most", (**Urban_**18-30**_Cervical**).

"I also thought about cancer but I had already tested and it was negative so they had told me to test again after three months - oh no! They said three years. I gave birth in 2013, so I tested in 2014. They had told me to go back in the previous year, 2018, but I haven't gone back again . . . I thought that probably it is the one starting. Because I heard that when you start experiencing smelly vaginal discharges that could be a sign of cancer. All I thought about was that it could be syphilis, if not, then cancer", (**Rural_**31-50**_Cervical**).

Severity and persistence of symptoms as well as messages from healthcare professionals informed and or reinforced participants' attributions of symptoms to possible cervical cancer.

For peer review only - http://bmjopen.bmj.com/site/about/guidelines.xhtml

For peer review only

"[My] lower back/abdominal pain made me think it was cervical cancer causing these symptoms. Because the lower abdominal pain was too much. But also I heard from the health facility. They were educating those who had gone to test for that cancer. The healthcare provider said that you can get this cancer from a man who has had sex with a woman who has the virus then he can transmit it to you through sex", (**Rural_18-30_Cervical**).

Persistence of bodily changes beyond ordinary expectations and severity of symptoms caused participants to review their attributions, often considering more serious illnesses including cancers.

"I thought probably it could be cancer because of the much pain I was experiencing on my breast, or that it was just normal pain resulting from the back pain I sometimes experience. But then sometimes I wonder why one side of my breast has a lump and the other doesn't; so all these leave me in wonder of what could be the problem", (**Urban_31-50_Breast**).

Participants who were aware of breast cancer symptoms immediately attributed the changes experienced in their breasts to possible breast cancer. Most reported that they commonly learn about symptoms of illness from radio programs.

"The reason as to why I thought it was breast cancer is because I always listen to the radio and these doctors always talk about cancer and how cancer can start and also the

For peer review only - http://bmjopen.bmj.com/site/about/guidelines.xhtml

signs and symptoms of cancer on people. So they tell you when you see such signs, please go to hospital", **(Urban_31-50_Breast)**.

Persistent and relentless pain not responding to commonly used medications or home-based remedies often led participants to think of more serious illnesses including cancers.

"I thought it could be cancerous because of the pain. The pain used not to cool before I went to the hospital; so I thought it was the toughest disease, cancer [. . .]. At least HIV/AIDS, it is easy to get drugs for it, but this cancer has no drug, till you die and get buried; and that is its medicine", (**Urban_51-70_Breast**).

Sexually transmitted infections and other gynaecologic infections

Participants who had symptoms of vaginal itching and discharge readily attributed the cause to candidiasis, believed to be transmitted both through sexual intercourse and poor personal hygiene. Some attributed their bodily changes to sexually transmitted infections, and blamed symptoms on their husbands who were often considered promiscuous.

"I thought that it could be some sexually transmitted infection. I was thinking that it could be gonorrhoea because if it were syphilis, I would have known it because it comes with a lot of pain, and I don't have the smelly vaginal discharge", (**Urban_18-30_Cervical**).

"I thought that probably my husband cheated on me with an infected person because I had never experienced these symptoms. […] ever since he was away for four months from October to February without coming home even one single day, then when he

For peer review only - http://bmjopen.bmj.com/site/about/guidelines.xhtml

comes back after sometimes, I start experiencing these symptoms! So I had every reasons to be suspicious”, (**Rural_31-50_Cervical**).

(v) Help-seeking for symptoms

Women sought help from both biomedical and traditional health practitioners including faith-based healers. Participants chose a particular help-seeking route depending on perceived causes, knowledge of previous similar illnesses that responded to a particular treatment approach, advice from social networks, and messages from the media especially local radio health programs.

Healthcare help-seeking

Most participants sought care in healthcare facilities because they needed tests to confirm the causes of their health problems; they felt these would direct them to the appropriate remedies.

“I went to the doctor because I know he is the only person who can help with my condition; they have testing kits and they can confirm without doubts. They have machines that test and confirm the truth. Those things that are just guessed are not helpful at all”, (**Urban_31-50_Cervical**).

Most sought care in healthcare facilities whenever symptoms worsened and or caused worries, or when home-based remedies did not lead to improvement in symptoms. Persistent pain was a very common trigger for healthcare seeking. The media, especially radio talk shows, encouraged a significant proportion of participants to seek care at health facilities.

For peer review only - http://bmjopen.bmj.com/site/about/guidelines.xhtml

"For me the first signs I saw was the swelling of my breast and I warmed water and started massaging on the swollen part but it didn't help, so I decided to go to hospital and told the doctor. Another reason as to why I visited the hospital with the symptoms of my sickness is because of the constant teaching and reminders by doctors over the radio during their radio talk shows that whenever you have any signs and symptoms which is unusual, you should go to hospital. […] I believe if you go to the hospital early enough with any sickness the doctors will find the name of the sickness and cure you if possible", (**Urban_31-50_Breast**).

"I went to the clinic, bought medicine and took it. But there was no improvement that's why I decided to go to the hospital. It was in March this year (2018) that I went to the clinic; but this pain started after giving birth. What made me go to the hospital was because I was worried. I was worried that I could be having a sexually transmitted disease or cervical cancer", (**Urban_18-30_Cervical**).

Advice from healthcare professionals when women go for antenatal and postnatal care, and immunization for their children motivated them to seek care whenever they experienced symptoms of illnesses.

"They say it; doctors always talk when we take our children for immunization. Those responsible for the hygiene and sanitation of the hospital once you take your children for immunization or antenatal care, they often tell us that once we get to see any funny signs of any sickness, we need to rush to the nearest health facility early enough. Otherwise the symptoms will worsen, because if you feel pain in your body, you need

For peer review only - http://bmjopen.bmj.com/site/about/guidelines.xhtml

to go to the hospital. So I took that up and rushed to the hospital because I was also feeling that in my body", (**Rural_31-50_Cervical**).

Participants were also motivated to seek biomedical care for concerns regarding the welfare of their children. They would want to remain alive and nurture their children their own way.

"The reason why I decided to go to the hospital that time when I was feeling the symptoms of my lower abdomen and the waist, is because I decided that I should stay healthy . . . to care for my children. I should surely remain healthy and keep them", (**Rural_31-50_Cervical**).

Perceived inconveniences to others and personal discomfort due to symptoms was another factor that triggered healthcare seeking among participants. Symptoms of cervical cancer including smelly vaginal discharge and excessive vaginal bleeding can be very inconveniencing and restrictive to the movement of a woman.

"I went because I was not comfortable among my fellow girls; you could see others are happy and for me I have my hidden problems, so I had to go and solve it with the doctor", (**Urban_18-30_Cervical**).

Traditional and complementary medicines

A few participants sought care from traditional health practitioners including herbalists and faith-based healers who used prayers, sometimes combined with herbal preparations to treat different ailments. Some participants thought traditional and complementary medicines (T&CMs) could be useful while others contended that T&CMs are fake, and that the

For peer review only - http://bmjopen.bmj.com/site/about/guidelines.xhtml

practitioners are dishonest. Most participants thought that T&CMs do not work. They said the medicines are given based on guess work, as there are no tests done to identify cause of illness. These negative views about T&CMs were held by participants from both urban and rural sites, and with both breast and cervical symptoms.

> "I don't even think about it because some usually walk past here hawking herbal drugs claiming they have what treats ulcers, blood pressure, cancer and many more diseases, but I always tell them I don't have money to buy and that I don't use herbs. There is nothing that can attract me in buying local herbs. My opinion is that traditional herbs nowadays don't work or not effective", (**Urban_31-50_Breast**).

> "With traditional medicines, you can be given just anything that will not even help you. So I will never make a commitment to use traditional medicine. They may even just go and pluck the leaves of black jack then they brand it that it is medicine for such and such a disease and yet in actual sense, it will not be the cure for that disease. So most often I stick to my decision of going to the hospital because their medicines will have been tested", (**Urban_31-50_Cervical**).

> "I haven't got any medicine from any traditional healer and I personally don't want. To tell you, these traditional medicines even if you are given in regards to these symptoms in the stomach, they will not work . . . So I really wonder whether the traditional medicines really treat those diseases", (**Rural_31-50_Cervical**).

A few of the participants who admitted to using T&CMs testified that T&CMs, the ones they used, did not work.

For peer review only

"I don't support that, and . . . I will stop T&CM use because I personally tried and it failed to work; it didn't help me with anything. It is true . . . I was instructed to go get the traditional medicine . . . I first went to that other T&CM practitioner, then I went to the nurse", (**Urban_18-30_Breast**).

"I tried taking traditional medicine in vain, and when I tried to use that without success, then my husband told me that its better I try to go to the hospital instead so that it is the doctor to feed me with the right information. I used to dig out the roots then I would drink the juice. No improvement was realized", (**Rural_51-70_Cervical**).

On the other hand, a substantial minority of participants who had ever used a T&CM reported that they are effective and can be used alongside modern medicine. They felt that when the cause of an illness is due to malevolent spirits or breaching cultural norms, then traditional health practitioners are helpful. Participants from the rural site were more likely to report use of T&CM than those from the urban site.

"When I used to pass sticky stool and also felt pain in the lower abdomen, the thing that helped me the most was one traditional medicine . . . it was called *tusua* (not real name). That is a type of tree . . . I would dig out the roots then pound it until fine, then I boil for long, then I filter it into the cup, and afterwards when it cools I take it, then it would end up the stickiness of my stool immediately. I also used to have tiny itchy rashes in between my thighs. […] I used to break the leaves of *okala* tree (not real name). You know that tree has some whitish sap, so I would harvest that and place on the spot of

For peer review only - http://bmjopen.bmj.com/site/about/guidelines.xhtml

> the blisters and the sap from *okala* ended up curing, till to date there are no rashes", (**Rural_31-50_Cervical**).

(vi) Perceived barriers to healthcare help-seeking

Participants discussed several barriers to prompt healthcare help-seeking. These included health system challenges including lack of medicines, long waiting time to see healthcare professionals, lack of money, and competing social obligations.

Lack of medicines in the public health facilities

Most participants who contemplated healthcare help-seeking for their symptoms lamented the sorry state of the public healthcare facilities. They reported that there were often no medicines available.

> "I just get discouraged with the thought of going there and being told to buy the recommended drugs from outside", (**Urban_51-70_Breast**).

> "They kept telling me that the drugs they need to inject me with is out of stock! I went back two times and each time the same story, so I never went back again", (**Urban_18-30_Cervical**).

Long waiting times

Due to the long queues and extended waiting times to see the healthcare professionals, especially in the public health facilities, participants often said they would consider alternative avenues to healthcare help-seeking. This view was more common among participants with breast symptoms especially from the urban site.

For peer review only - http://bmjopen.bmj.com/site/about/guidelines.xhtml

"The main referral hospital also has its own problems; you can take like two days while still on the line to see the doctor. It is equivalent to someone coming all the way from Sudan and going back; that is the line to see the doctor. Whenever you are almost at the front of the line, the doctor leaves. So when you combine all the waiting with hunger and ulcers, you end up giving up", (**Urban_51-70_Breast**).

"I'm just planning to go in this coming February. [The] hospital is usually very crowded; if you go there you can spend a long time without getting treatment", (**Rural_18-30_Cervical**).

Absence of healthcare professionals at the healthcare facilities

Participants from both urban and rural sites reported visiting healthcare facilities several times but not finding the appropriate healthcare professionals to address their concerns.

"As you know these government hospitals, services are not like for other private hospitals. So the experience I had was - the first time I went to the hospital, they recommended that I first have a scan done, but the person who was supposed to do it wasn't around so they told me to go back the next day, which I did and they told me the person supposed to do it was still not there so I should go back on Monday, (the following week)", (**Urban_31-50_Breast**).

"I went and the healthcare provider picked the referral note and put it in the file where they had written my complaints. Then I was told that the doctor isn't there and that I should just go back on Friday, when the doctor will be around and also the ultra-scan

For peer review only - http://bmjopen.bmj.com/site/about/guidelines.xhtml

machine will also be working, so that they can also do an ultra-scan the next Friday", (**Rural_18-30_Cervical**).

Lack of money for healthcare help-seeking

Most participants reported financial constraints to seeking healthcare in the private facilities where there would be no crowding, and medicines available, and described unaffordable fees for consultations, tests and treatments.

"There is really pain, but the main reason why I didn't go is the lack of finances that opens every door", (**Rural_31-50_Breast**).

Participants reported that patients sometimes had to sell whatever they have to get some money to seek care in the private health facilities where they expect reasonable care.

"There is nothing else, because for the three times I visited [the private] hospital, and two times in the government hospital I have been selling goats of which they are now finished. There is nothing more to sell, and yet I have not yet received any improvement", (**Rural_31-50_Breast**).

Rural participants also mentioned competing demands for the little money they got from selling off their belongings to support their medical expenses.

"So when I get money I will seek for help in the health facility but for now it's tricky because I have grown up children and grandchildren who are under my care. So getting the money to go to the health facility is a problem. And as you also know *Lexam* (not real name) hospital, all the services there require money. […] I plan to go to *Lexam*

For peer review only - http://bmjopen.bmj.com/site/about/guidelines.xhtml

hospital because *Takila* regional referral hospital (not real name) is quite far away, *Lexam* is nearer to our home here", (**Rural_51-70_Breast**).

"It is the issue of lack of money that prevented me from going. […] I am struggling so that I raise some money to buy books for my children and enrol them in school then I will think of going to the hospital. I do want to seek help but the means to access that is what is still defeating me. I need, because I also love my body. It is only the issue of money and if I had it today, I would go straight to the hospital", (**Rural_31-50_Cervical**).

Transport money to the public health facilities was another challenge for women, especially those from the rural areas.

"I'm still looking for money to go with to the hospital. I will go when I get the money. The money is for transport. It is 12,000= (USD 3.3) one way, so I need 24,000= (USD 6.6) for transport on a boda boda (*hired motor cycle*)", (**Rural_18-30_Cervical**).

For peer review only - http://bmjopen.bmj.com/site/about/guidelines.xhtml

Discussions

Main findings

This study is one of the first community-based inquiries into symptom recognition, interpretation, appraisal, illness attribution, and consequent help-seeking among women with possible breast and cervical cancer symptoms in urban and rural Uganda. We found that women undergo a complex process of sense-making and help-seeking for their symptoms. The detection and interpretation of abnormal bodily sensations were interpreted based on expected bodily changes within a person's lifespan, perceived poor personal hygiene, and crowded living environments with risk of infections. Upon realising abnormal bodily changes, most participants engaged in extensive lay consultations with relatives, friends and acquaintances mainly because they wanted to solicit financial and practical support, learn and benefit from experiences of others regarding the symptoms, and for social accountability and avoidance of suspicion from husbands/families especially when symptoms were disabling and restricting participations in their daily chores. On the other hand, a substantial minority of participants did not disclose their symptoms to lay colleagues mainly because of low expectations of support, concerns with privacy, avoidance of possibility of gossiping, and concern with stigmatization from the community. Medical pluralism was common, with participants often seeking help for symptoms from both biomedical healthcare facilities and traditional and complementary medicine providers including faith-based healers, despite many being sceptical about the effectiveness of traditional and complementary medicines.

Our findings regarding recognition of abnormal bodily changes, interpretation and symptom attributions, as well as disclosure/nondisclosure of symptoms to lay people, are similar to findings from studies in other SSA countries such as Malawi and Ghana among breast cancer patients.(13, 38) The patients appraised their symptoms within the context of their life events

For peer review only - http://bmjopen.bmj.com/site/about/guidelines.xhtml

and situations, and were guided by expected bodily changes, family history of cancer and suggestions from lay consultations. The attribution of symptoms changed over time depending on characteristics of the symptoms, including their nature, evolution, response/nonresponse to common medications, and advice from lay people. Understanding the life context of people could inform the content, delivery and timing of health interventions to promote prompt and appropriate symptom recognition and help-seeking.

Our findings suggest that Ugandan women with vaginal symptoms often first attribute their symptoms to gynaecologic infections such as candidiasis and sexually transmitted infections. Only if symptoms persisted or did not respond to medications did women sometime attribute their symptoms to cancer. Similar symptoms for cancers and infectious diseases can offer great challenges to women especially if the illnesses have different pathways to care and treatment. In our earlier studies among women with an established diagnosis of cervical cancer in a hospital in the same region, most women attributed their vaginal symptoms of bleeding and discharge to cervical cancer and sexually transmitted infections. Most of these women sought care after long time periods and presented with advanced stage cancer.(4) In contrast, we found that women with breast symptoms who had heard of breast cancer mainly from radios and healthcare professionals during earlier visits to health facilities quickly attributed their symptoms to breast cancer and sought care accordingly. Similar findings were reported in an earlier Ugandan study about symptoms of cervical cancer.(12) This concurs with evidence from the UK, showing that awareness and knowledge of cancer symptoms are key in shaping people's attributions of symptoms and often prompt healthcare help-seeking.(39) We also found that pain, persistence of symptoms, or symptoms interfering with daily routines could trigger decisions to seek help. Mild and intermittent symptoms were either ignored or

For peer review only - http://bmjopen.bmj.com/site/about/guidelines.xhtml

interpreted as normal bodily changes, for example, due to the menopause or pregnancy, concurring with findings from a Malawi study of women with breast cancer.(13)

Women with potential breast and cervical cancer symptoms often engaged in lay consultations with their husbands, relatives and friends regarding their symptoms. These lay consultations were found to not only influence symptom attributions and illness inference, but also women's' help-seeking intentions and behaviour. Participants consulted with other people to obtain financial and practical support to seek help, as well as to gain insights into the causes and best effective remedies for their illnesses. Sanctioning of initial symptom interpretations by significant others has been shown in other studies set in the UK and Australia to influence help-seeking decisions among people with possible cancer symptoms or recently diagnosed with cancer.(40, 41) To avoid misleading advice when symptomatic women seek help from peers and significant others, the community as a whole need to be aware of cancer risk factors, symptoms and treatment. Therefore, public awareness campaigns to improve general awareness is important so that when women share their symptoms with other lay people, they may get appropriate help.

We found surprising negativity about traditional and complementary medicines, although some continued to consult traditional healers. This contrasts with earlier Ugandan qualitative findings from asymptomatic men and women, revealing that traditional and complementary medicines occupied a reasonable place in their lives mainly because the medicines were believed to work as they worked for their grandparents and ancestors, and could be useful to fill the void created by multiple barriers to biomedical care.(42) The participants in this study perceived the research assistants as healthcare providers; indeed, one was a nurse. Therefore,

For peer review only - http://bmjopen.bmj.com/site/about/guidelines.xhtml

it is possible that the reported low opinion towards use of traditional and complementary medicines could be due to social desirability bias.

Participants in this study reported several individual, interpersonal, sociocultural and health system structural barriers that delayed or prevented them from seeking healthcare. In public health facilities these barriers included lack of medicines, long waiting times and queues, and absenteeism among healthcare professionals. At a personal level, women commonly reported lack of money for transport and medical bills as their main barrier, especially in private healthcare facilities where patients pay directly for care. Most women also reported competing priorities and obligations that would hinder them from seeking healthcare. These obligations not only competed for their time but also for financial resources which were often diverted to school fees and other priorities instead of transport to health facilities and medical bills. Similar challenges to breast and cervical cancer healthcare help-seeking have been reported in other studies in SSA.(13, 14, 42) Insights into the perceived barriers to, and contextual factors that influence healthcare seeking from perspectives of the women can potentially inform targeted interventions to promote prompt healthcare seeking. Imposing health programs on women without clearly understanding their perspectives and contextual factors may not have the desired impact and would be an inefficient use of resources. Health planners and policy makers therefore need to make research findings to inform their decisions.

Limitations

Our study has some limitations. The study was preceded by a cross-sectional survey in which women were asked about potential breast and cervical cancer symptoms. It is possible that this could have influenced how women perceived and interpreted their symptoms.

For peer review only - http://bmjopen.bmj.com/site/about/guidelines.xhtml

Second, conducting interviews in women's homes may have influenced how some participants responded particularly in terms of the influence of family and social networks. However, we ensured that there were no non-participants at the interview venues.

Third, self-report of symptoms and help-seeking is subject to social desirability bias especially when participants may consider that their perceptions, beliefs and conduct could attract unfavourable comments from the research team or other people. However, as the research participants were assured of confidentiality, we contend that our findings accurately represent the perceptions and help-seeking behaviour of the symptomatic women included in this study.

Conclusion

Symptom attributions and consequent help-seeking may be influenced by information from radios or healthcare professionals, previous experiences will similar illnesses, approval or sanctioning by lay colleagues, and the onset, nature, persistence and severity of their symptoms as well as their response to medications. We found several interrelated sociocultural and structural barriers to healthcare help-seeking. In order to promote timely help-seeking and early detection of symptomatic breast and cervical cancer, targeted public health messages and campaigns to increase knowledge of women on breast and cervical cancer symptoms and services available need to be coupled with economic empowerment of women and improvement in access to health systems.

Acknowledgement

The authors wish to appreciate the research assistants and field coordinator for their thoroughness in conducting interviews in the midst of challenges including impassable roads and traversing difficult terrains on motorcycles. We are equally indebted to the research participants who freely discussed their ideas on the research questions, thereby providing us

For peer review only - http://bmjopen.bmj.com/site/about/guidelines.xhtml

with detailed information. We also thank the local leaders for their cooperation and participation in mobilizations and guidance for security and safety of research assistants during field visits.

Contributors Study concept and design: JM, FMW and ADM; acquisition of data: ADM and HW; analysis of data: ADM, JH and SS; interpretation of data: all authors; drafting of the manuscript: ADM; critical revision of the manuscript for important intellectual content: all authors; study supervision: JM, FMW, ADM and HW. Final approval of the submitted version of the manuscript and where to submit: all authors.

Funding Research reported in this article was jointly supported by the Cancer Association of South Africa (CANSA), the University of Cape Town and the South African Medical Research Council with funds received from the South African National Department of Health, GlaxoSmithKline (GSK) Africa Non-Communicable Disease Open Lab (via a supporting grant Project Number: 023), the United Kingdom Medical Research Council, MRC (via the Newton Fund). GSK provided in-kind scientific and statistical support as part of capacity strengthening. Award/Grant number is not applicable. The funders had no role in study design, data collection and analysis, preparation of the manuscript, decision to publish and where to publish. FMW is Director and SES is co-investigator of the multi-institutional CanTest Collaborative, which is funded by Cancer Research UK (C8640/A23385).

Competing Interests The authors declare that they have no competing interests.

Patient consent Not applicable.

For peer review only - http://bmjopen.bmj.com/site/about/guidelines.xhtml

Ethical approval This study protocol was approved by the Lacor Hospital Institutional Research Ethics Committee (**LHIREC 027/11/2016**). The protocol was thereafter registered and cleared by the Uganda National Council for Science and Technology (UNCST), registration number **HS60ES**. Additional permission to enter into the study communities was obtained from the local leaders at the district and sub-county levels. Written informed consents were obtained form study participants before interviews. Participants received a token of 25,000= (USD 6.8) for light snacks and soft drinks during interviews.

Disclaimers None.

Data sharing statement No additional data are available. Study dataset can be made available to third party following reasonable request to corresponding author.

For peer review only

References

1. Bray F, Ferlay J, Soerjomataram I, Siegel RL, Torre LA, Jemal A. Global cancer statistics 2018: GLOBOCAN estimates of incidence and mortality worldwide for 36 cancers in 185 countries. CA: a cancer journal for clinicians. 2018.
2. Mabula JB, Mchembe MD, Chalya PL, Giiti G, Chandika AB, Rambau PF, et al. Stage at diagnosis, clinicopathological and treatment patterns of breast cancer at Bugando Medical Centre in north-western Tanzania. Tanzania Journal of Health Research. 2012;14(4).
3. Pace LE, Mpunga T, Hategekimana V, Dusengimana J-MV, Habineza H, Bigirimana JB, et al. Delays in Breast Cancer Presentation and Diagnosis at Two Rural Cancer Referral Centers in Rwanda. The Oncologist. 2015;20(7):780-8.
4. Mwaka AD, Garimoi CO, Were EM, Roland M, Wabinga H, Lyratzopoulos G. Social, demographic and healthcare factors associated with stage at diagnosis of cervical cancer: cross-sectional study in a tertiary hospital in Northern Uganda. BMJ Open. 2016;6(1).
5. Dunyo P, Effah K, Udofia EA. Factors associated with late presentation of cervical cancer cases at a district hospital: a retrospective study. BMC Public Health. 2018;18(1):1156.
6. Joko-Fru WY, Miranda-Filho A, Soerjomataram I, Egue M, Akele-Akpo M-T, N'da G, et al. Breast cancer survival in sub-Saharan Africa by age, stage at diagnosis and human development index: A population-based registry study. Int Journal of Cancer. 2019.
7. Wabinga H, Ramanakumar AV, Banura C, Luwaga A, Nambooze S, Parkin DM. Survival of cervix cancer patients in Kampala, Uganda: 1995-1997. Brit Journal of Cancer. 2003;89(1):65-9.
8. Gondos A, Chokunonga E, Brenner H, Parkin DM, Sankila R, Borok MZ, et al. Cancer survival in a southern African urban population. Int Journal of Cancer. 2004;112(5):860-4.
9. Gondos A, Brenner H, Wabinga H, Parkin DM. Cancer survival in Kampala, Uganda. Brit Journal of Cancer. 2005;92(9):1808-12.
10. Sankaranarayanan R, Swaminathan R, Lucas E. Cancer survival in Africa, Asia, the Caribbean and Central America: International Agency for Research on Cancer Lyon; 2011.
11. van Schalkwyk SL, Maree JE, Wright SC. Cervical cancer: the route from signs and symptoms to treatment in South Africa. Reproductive health matters. 2008;16(32):9-17.
12. Mwaka AD, Okello ES, Wabinga H, Walter FM. Symptomatic presentation with cervical cancer in Uganda: a qualitative study assessing the pathways to diagnosis in a low-income country. BMC Women's Health. 2015;15(1):15.
13. Kohler RE, Gopal S, Miller AR, Lee CN, Reeve BB, Weiner BJ, et al. A framework for improving early detection of breast cancer in sub-Saharan Africa: A qualitative study of help-seeking behaviors among Malawian women. Patient education and counseling. 2017;100(1):167-73.
14. Mwaka AD, Wabinga HR, Mayanja-Kizza H. Mind the gaps: a qualitative study of perceptions of healthcare professionals on challenges and proposed remedies for cervical cancer help-seeking in post conflict northern Uganda. BMC Family Practice. 2013;14:193.
15. Gebremariam A, Addissie A, Worku A, Assefa M, Pace LE, Kantelhardt EJ, et al. Time intervals experienced between first symptom recognition and pathologic diagnosis of breast cancer in Addis Ababa, Ethiopia: a cross-sectional study. BMJ Open. 2019;9(11):e032228.
16. Ibrahim NA, Oludara MA. Socio-demographic factors and reasons associated with delay in breast cancer presentation: A study in Nigerian women. The Breast. 2012;21(3):416-8.

For peer review only - http://bmjopen.bmj.com/site/about/guidelines.xhtml

17. Brinton L, Figueroa J, Adjei E, Ansong D, Biritwum R, Edusei L, et al. Factors contributing to delays in diagnosis of breast cancers in Ghana, West Africa. Breast Cancer Res Tr. 2017;162(1):105-14.

18. Asoogo C, Duma SE. Factors contributing to late breast cancer presentation for health care amongst women in Kumasi, Ghana. Curationis. 2015;38:1-7.

19. Walter FM, Webster A, Scott S, Emery J. The Andersen Model of Total Patient Delay: a systematic review of its application in cancer diagnosis. Journal of Health Services Research & Policy. 2012;17.

20. Scott SE, Walter FM, Webster A, Sutton S, Emery J. The model of pathways to treatment: conceptualization and integration with existing theory. Br J Health Psychol. 2013;18(1):45-65.

21. Niksic M, Rachet B, Duffy SW, Quaresma M, Møller H, Forbes LJL. Is cancer survival associated with cancer symptom awareness and barriers to seeking medical help in England? An ecological study. Brit Journal of Cancer. 2016;115(7):876-86.

22. Joffe M, Ayeni O, Norris SA, McCormack VA, Ruff P, Das I, et al. Barriers to early presentation of breast cancer among women in Soweto, South Africa. PloS One. 2018;13(2):e0192071.

23. Were EO, Buziba NG. Presentation and health care seeking behaviour of patients with cervical cancer seen at Moi Teaching and Referral Hospital, Eldoret, Kenya. East African medical journal. 2001;78(2):55-9.

24. Kazaura MR, Kombe D, Yuma S, Mtiro H, Mlawa G. Health seeking behavior among cancer patients attending Ocean Road Cancer Institute, Tanzania. East African journal of public health. 2007;4(1):19-22.

25. Birhanu Z, Abdissa A, Belachew T, Deribew A, Segni H, Tsu V, et al. Health seeking behavior for cervical cancer in Ethiopia: a qualitative study. International journal for equity in health. 2012;11:83.

26. Powe BD. Fatalism among elderly African Americans: Effects on colorectal cancer screening. Cancer nursing. 1995;18(5):385-92.

27. Beeken RJ, Simon AE, von Wagner C, Whitaker KL, Wardle J. Cancer fatalism: deterring early presentation and increasing social inequalities? Cancer Epidemiology Biomarkers & Prevention. 2011;20(10):2127-31.

28. Asoogo C, Duma SE. Factors contributing to late breast cancer presentation for health care amongst women in Kumasi, Ghana. Curationis. 2015;38(1):1287.

29. Drews CD, Greeland S. The Impact of Differential Recall on the Results of Case-Control Studies. International journal of epidemiology. 1990;19(4):1107-12.

30. Kopec JA, Esdaile JM. Bias in case-control studies. A review. Journal of epidemiology and community health. 1990;44(3):179-86.

31. MoFPED. POVERTY STATUS REPORT 2014. Structural change and poverty reduction in Uganda. Available at: https://wwwtralacorg/images/docs/6878/uganda-poverty-status-report-november-2014pdf. 2014;Accessed on 2nd June 2020.

32. MOH. Ministry of Health (MOH) [Uganda] and ORC Macro. 2006. Uganda HIV/AIDS Sero-behavioural Survey 2004-2005. Calverton, Maryland, USA: Ministry of Health and ORC Macro. Available at: https://dhsprogramcom/pubs/pdf/AIS2/AIS2pdf Accessed 2nd June 2020. 2006.

33. Patel S, Schechter MT, Sewankambo NK, Atim S, Kiwanuka N, Spittal PM. Lost in Transition: HIV Prevalence and Correlates of Infection among Young People Living in Post-Emergency Phase Transit Camps in Gulu District, Northern Uganda. PloS One. 2014;9(2):e89786.

34. Moodley J, Scott SE, Mwaka AD, Constant D. Development and validation of the African Women Awareness of CANcer (AWACAN) tool for breast and cervical cancer. 2019;14(8):e0220545.

For peer review only - http://bmjopen.bmj.com/site/about/guidelines.xhtml

35. Moodley J, Constant D, Mwaka AD, Scott SE, Walter FM. Mapping awareness of breast and cervical cancer risk factors, symptoms and lay beliefs in Uganda and South Africa. PLoS One. 2020;15(10):e0240788.

36. Francis JJ, Johnston M, Robertson C, Glidewell L, Entwistle V, Eccles MP, et al. What is an adequate sample size? Operationalising data saturation for theory-based interview studies. Psychology & health. 2010;25(10):1229-45.

37. Guest G, Bunce A, Johnson L. How Many Interviews Are Enough?: An Experiment with Data Saturation and Variability. Field Methods. 2006;18(1):59-82.

38. Bonsu AB, Ncama BP. Recognizing and appraising symptoms of breast cancer as a reason for delayed presentation in Ghanaian women: A qualitative study. PloS One. 2019;14(1):e0208773.

39. Simon A, Waller J, Robb K, Wardle J. Patient Delay in Presentation of Possible Cancer Symptoms: The Contribution of Knowledge and Attitudes in a Population Sample from the United Kingdom. Cancer Epidemiol Biomarkers Prev. 2010;19.

40. Macleod U, Mitchell ED, Burgess C, Macdonald S, Ramirez AJ. Risk factors for delayed presentation and referral of symptomatic cancer: evidence for common cancers. Br J Cancer. 2009;101 Suppl 2:S92-s101.

41. Emery JD, Walter FM, Gray V, Sinclair C, Howting D, Bulsara M, et al. Diagnosing cancer in the bush: a mixed-methods study of symptom appraisal and help-seeking behaviour in people with cancer from rural Western Australia. Family Practice. 2013.

42. Mwaka AD, Okello ES, Orach CG. Barriers to biomedical care and use of traditional medicines for treatment of cervical cancer: an exploratory qualitative study in northern Uganda. Eur Journal of Cancer Care. 2015;24(4):503-13.

For peer review only

In-depth Interview Guide

Understanding symptoms appraisal, help seeking and perceived barriers to healthcare seeking among women with potential symptoms of breast and cervical cancer in Uganda

Introduction

Thank you for accepting to participate in this study. The study is about understanding the way women with symptoms that could be due to breast or cervical cancer explain their symptoms and seek help for the them. In particular, we would like to understand the things that you consider when you are want to explain such symptoms as breast pain, swelling in the breast and discharge from the genitals o a woman. We also need to understand from you the factors that influence the way you seek for help.

To achieve our objective, we shall conduct an interview with you based on questions in our study guide. This interview will take about 45 minutes to one and half hours. Please keep in mind that there are no right or wrong answers to these questions. If I raise an issue or ask a question you don't want to talk about, just say so and we will move on to something else. Plase be free to ask any questions you will like to ask to help you undertand our interview. If you have any questions about the study, you can ask them below starting he interview or afterwards.

Note that you have been invited to participate in this interview because you have indicated that you have had some symptoms including pain and welling in the breasts, and discharge from your genitals during our earlier study. We are very grateful to have participate in this segment of the study.

For peer review only

Guiding questions

A: Detection and appraisal of bodily changes

1. **Thank you for agreeing to be interviewed today. I would like to start by asking you some questions around the changes/symptoms you mention related to your … (here relate to specific symptom mentioned e.g. breast lump, vaginal discharge).**
   Could you tell me more about this change?
   Probes:
   - What was the first thing that you noticed?
   - When did you notice the symptom? Use calendar prompt to assist, based on important landmarks e.g. Christmas, Easter, public holidays etc.
   - Has the symptom changed since you first noticed it? If yes, how has this changed?

2. **Could you describe how you felt when you noticed this symptom (refer to change/symptom e.g. finding a breast lump, vaginal bleeding)?**
   Probe:
   - Emotional response to symptom? E.g. Afraid, anxious, embarrassed, not concerned, denial, concerned that it was serious
   - Why did you feel that way?

For peer review only - http://bmjopen.bmj.com/site/about/guidelines.xhtml

3. **When you first noticed the symptom(s), what did you think was causing the symptom? What made you think that?**
   Probe:
   - Did you think it meant you had any particular illness? If so what illness and what made you think this way?
   - At any point did you think it is/was an infection? Why did you think this?
   - Did you think it was something minor/nothing to worry about? If so, what made you think this?
   - Were you unsure about what it meant? If so what made you unsure?

4. **Since then, have you had any other thoughts about what it might be? (if changed thoughts of what the symptoms could be caused by then, probe reasons for change. E.g. what made you think that?)**

5. **Have you had a symptom like this before? If yes Probe when? What did you do? What was the outcome?**

6. **Do you know of anyone who had a similar symptom? [say the symptom]**

   If Yes, what was the cause of their symptom? What did they do about it? What was the outcome?

7. **Have you ever heard about this symptom/change anywhere? If yes where?**
   Probe: at the clinic, discussed with my family/friends, pamphlets, social media/ posters taxis or trains/ radio/television? What information did each of these sources provide about such a symptom?

8. **Did you discuss this current change/symptom with anyone?**
   Probe:
   If Yes
   - Whom?
   - What did this person/s say? Was it helpful?
   - Did it influence your views on the change/symptom? If Yes how?
   - Why did you discuss with that person (name/relation)?

   If No
   - Why did you not discuss with anyone? Probes feelings of anxiety, not worried about symptom etc.

9. **How do you think people in your community view someone with this symptom (refer to symptoms participant named)**
   Probe:
   - Is there any stigma attached to having this symptom?
   - Are people supportive/empathetic with people with these kinds of symptoms in your community?

For peer review only - http://bmjopen.bmj.com/site/about/guidelines.xhtml

- Is this symptom generally discussed by family and friends?

10. Has this symptom affected your life in anyway?

Probe:
If yes, how has it affected your life?

11. Have you tried to manage the symptoms yourself? Did you try anything to help relieve / resolve the symptoms?

B: Help-seeking behaviour and estimated dates of symptoms

12. Have you gone to have this change/symptom (name symptom) checked by anyone?
If YES
Probes:

- Who did you visit?
- Why did you choose to go there? Could you tell me **what** made you decide to seek care? Was there anything in particular that made you decide to seek assistance?
- When did you go to have it checked? *Use calendar prompt*
- Can you tell me about your experience seeking advice or assistance from …

Have you had the symptom checked by anyone else? (Repeat prompts above)

- Have you visited a traditional healer? If visited traditional healer, was it after or before visiting the health facilities and why before or after (rationale)? What did the traditional healer say about the symptom? *Note participant might discuss this in earlier response to question 12.*

- Have you visited a health care facility or health care provider? If yes when did you visit health care provider? Probe only if person waited to see health care provider - Is there anything that made you wait before getting checked by a health care provider?

Note participant might discuss this in earlier response to question 12.

If NO (woman has not gone to seek care)

- Have you thought about getting checked?
- Do you intend to seek care? If so, from whom? When?
- Are there any reasons for not seeking care from a healthcare provider?
- Is there anything that has stopped you from seeking care?
  e.g. lack of pain, past history of this symptom, belief that it might go away, think/thought it is/was minor, partner, family or friends
- Access - Are there any problems getting to the clinic?
  - Is the cost of getting to a clinic or health care provider a problem?

For peer review only - http://bmjopen.bmj.com/site/about/guidelines.xhtml

- If person working is it a problem to take time off work?
- If person looking after children is it a problem to get to the clinic because you are looking after children

Is there anything that would make you get checked by a healthcare provider?

We are now coming to the end of our discussion. Can I take a minute to pause to see if there are any questions I might have missed?

13. Do you have any other comments, or questions that you would like to ask?

Thank you for participating in this study. Your views are very important to us.

For peer review only

For peer review only - http://bmjopen.bmj.com/site/about/guidelines.xhtml

Checklist: **Symptom appraisal, help seeking and perceived barriers to healthcare seeking in Uganda: An exploratory study among women with potential symptoms of breast and cervical cancer**

		Reporting Item	Page Number
**Title**			
	#1	Concise description of the nature and topic of the study identifying the study as qualitative or indicating the approach (e.g. ethnography, grounded theory) or data collection methods (e.g. interview, focus group) is recommended	1
**1Abstract**			
	#2	Summary of the key elements of the study using the abstract format of the intended publication; typically includes background, purpose, methods, results and conclusions	2
**Introduction**			
Problem formulation	#3	Description and significance of the problem / phenomenon studied: review of relevant theory and empirical work; problem statement	4 - 5
Purpose or research question	#4	Purpose of the study and specific objectives or questions	6
**Methods**			
Qualitative approach and research paradigm	#5	Qualitative approach (e.g. ethnography, grounded theory, case study, phenomenology, narrative research) and guiding theory if appropriate; identifying the research paradigm (e.g. postpositivist, constructivist / interpretivist) is also recommended; rationale. The rationale should briefly discuss the justification for choosing that theory, approach, method or technique rather than other options available; the assumptions and limitations implicit in those choices and how those choices influence study conclusions and transferability. As appropriate the rationale for several items might be discussed together.	6

For peer review only

Researcher characteristics and reflexivity	#6	Researchers' characteristics that may influence the research, including personal attributes, qualifications / experience, relationship with participants, assumptions and / or presuppositions; potential or actual interaction between researchers' characteristics and the research questions, approach, methods, results and / or transferability	N/A
Context	#7	Setting / site and salient contextual factors; rationale	6
Sampling strategy	#8	How and why research participants, documents, or events were selected; criteria for deciding when no further sampling was necessary (e.g. sampling saturation); rationale	7
Ethical issues pertaining to human subjects	#9	Documentation of approval by an appropriate ethics review board and participant consent, or explanation for lack thereof; other confidentiality and data security issues	35
Data collection methods	#10	Types of data collected; details of data collection procedures including (as appropriate) start and stop dates of data collection and analysis, iterative process, triangulation of sources / methods, and modification of procedures in response to evolving study findings; rationale	7
Data collection instruments and technologies	#11	Description of instruments (e.g. interview guides, questionnaires) and devices (e.g. audio recorders) used for data collection; if / how the instruments(s) changed over the course of the study	7
Units of study	#12	Number and relevant characteristics of participants, documents, or events included in the study; level of participation (could be reported in results)	7
Data processing	#13	Methods for processing data prior to and during analysis, including transcription, data entry, data management and security, verification of data integrity, data coding, and anonymisation / deidentification of excerpts	8
Data analysis	#14	Process by which inferences, themes, etc. were identified and developed, including the	8

For peer review only

		researchers involved in data analysis; usually references a specific paradigm or approach; rationale	
Techniques to enhance trustworthiness	#15	Techniques to enhance trustworthiness and credibility of data analysis (e.g. member checking, audit trail, triangulation); rationale	8
**Results/findings**			
Syntheses and interpretation	#16	Main findings (e.g. interpretations, inferences, and themes); might include development of a theory or model, or integration with prior research or theory	9
Links to empirical data	#17	Evidence (e.g. quotes, field notes, text excerpts, photographs) to substantiate analytic findings	9 - 28
**Discussion**			
Integration with prior work, implications, transferability and contribution(s) to the field	#18	Short summary of main findings; explanation of how findings and conclusions connect to, support, elaborate on, or challenge conclusions of earlier scholarship; discussion of scope of application / generalizability; identification of unique contributions(s) to scholarship in a discipline or field	29 - 32
Limitations	#19	Trustworthiness and limitations of findings	32 - 33
**Other**			
Conflicts of interest	#20	Potential sources of influence of perceived influence on study conduct and conclusions; how these were managed	34
Funding	#21	Sources of funding and other support; role of funders in data collection, interpretation and reporting	34

For peer review only
